# Analysis of Stemness and Prognosis of Subtypes in Breast Cancer Using the Transcriptome Sequencing Data

**DOI:** 10.1155/2022/5694033

**Published:** 2022-03-09

**Authors:** Wei Chen, Zhipeng Hong, Shaohong Kang, Xinying Lv, Chuangui Song

**Affiliations:** ^1^Department of Breast Surgery, Fujian Medical University Union Hospital, Fuzhou, Fujian Province 350001, China; ^2^Department of General Surgery, Fujian Medical University Union Hospital, Fuzhou, Fujian Province 350001, China; ^3^Breast Cancer Institute, Fujian Medical University, Fuzhou, Fujian Province 350001, China; ^4^Affiliated Quanzhou First Hospital of Fujian Medical University, Quanzhou, Fujian Province 362000, China

## Abstract

The stem characteristics of tumor cells have been proposed in theory very early, and we can use the signature of gene expression to speculate the stemness of tumor cells. However, systematic studies on the stemness of breast cancer as well as breast cancer subtypes, and the relationship between stemness and metastasis and prognosis, are still lacking. In the present research, using the transcriptome data of patients with breast cancer in the TCGA database, a stemness prediction model was utilized to derive the stemness of the patients' tumors. We compared the stemness values among different subtypes and the differences with metastasis. COX regression was employed to evaluate the correlation between stemness value as well as prognosis. Using the Lasso-penalized Cox regression machine learning model, we obtained the gene signature of the basal subtype that is related to stemness and can also predict the prognosis of the patient. Patients can be stratified into two groups of high and low stemness, corresponding to good and poor prognosis. Based on further prediction of tumor infiltration by CIBERSORT and prediction of drug response by a connectivity map, we found that the difference in stemness between these two groups is associated with the activation of tumor-killing immune cells and drug response. Our findings can promote the understanding and research of subtypes of basal breast cancer and provide corresponding molecular markers for clinical detection and therapy.

## 1. Introduction

According to statistics from global tumor data in 2018, breast cancer has the highest incidence and mortality among women [1]. With the development of science and technology, the ability to identify and diagnose breast cancer has significantly improved, and the past anatomy-based treatment is shifting to diagnosis and treatment through different biological mechanisms. Gene array technology divides breast cancer into different biological subtypes. New systemic drugs have significantly improved survival rates and are expected to enable patients with metastatic tumors to survive longer.

There are five subtypes of breast cancer, LumA, LumB, Basal, HER2, and normal. Among them, LumA and LumB subtypes have many types of mutations in the key genes, and the overall gene mutation rate of Basal and HER2 subtypes is higher than others. Different subtypes have different molecular expression profiles. According to the gene expression in cells, we can diagnose and treat patients accordingly. In terms of relationship with prognosis, Luminal has a better overall prognosis, and HER2 subtype has a worse overall prognosis and will relapse early. The Basal subtype has the worst prognosis. Moreover, as it is triple-negative, the only treatment option is chemotherapy. The normal subtype has a prognosis between Luminal and Basal and is not sensitive to chemotherapy [2].

Stemness, considered as the capacity to self-renew and differentiate from the precursor cells, was initially explored in normal stem cell-related studies, which has the capability to produce all cell types in adult organisms [3]. A significant proportion of genomic, proteomic, epigenomic, and transcriptomic markers have been shown to be related to cancer stemness in recent years. Over the last ten years, The Cancer Genome Atlas (TCGA) has shed light on the molecular environments of primary tumors by delivering thorough molecular profiles that include epigenomic, transcriptomic, genomic, and (post-translational) proteomic properties [4, 5], as well as clinical and histopathological annotations. The materials generated on the basis of the TCGA data enable us to thoroughly examine the cancer stem in a large sample of breast tumors and their subtypes.

The objective of this study was to perform cell stemness calculations using TCGA breast cancer data and to explore the relationship between cell stemness and prognosis. After subtyping the highly relevant factors, the signatures that can predict prognosis are finally calculated. The biological significance and clinical value of these predicted signatures are analyzed to provide a theoretical basis and reference basis for further research.

## 2. Materials and Methods

### 2.1. Collection and Processing of Data

#### 2.1.1. Breast Cancer Data

We acquired breast cancer clinical data through the GDC Data Portal with a total of 1097 valid patients and collected clinical source data as follows: clinical.tsv containing the latest updated prognostic information and the more detailed information was in https://nationwidechildrens.org_clinical_patient_brca.txt. In addition, molecular subtypes of breast cancer samples were obtained through TCGAbiolinks, and those with complete clinical and subtype information (*n* = 1095) were selected for subsequent integration and analysis (Table 1). Through TCGAbiolinks, the expression data of breast cancer samples including mRNA and lncRNA were obtained.

#### 2.1.2. Stem Cell Data

The Synapse is the portal for the Progenitor Cell Biology Consortium (PCBC), an NHLBI sponsored endeavor to discover and define progenitor cell lineages, to govern the development of stem and progenitor cells into ideal cell fates, and to create innovative ways to resolve certain problems when these cells are transplanted. Stem cell gene expression, methylation, SNV (copy-number variation), and other information are stored on the Synapse database. Synapse provides a variety of interfaces, and researchers can obtain them on the platform through R, Python, and other software and share these data.

### 2.2. Calculate Sample Stemness Index According to mRNA Expression

Malta et al. [3] developed a prediction model by means of the OCLR algorithm on pluripotent stem cell samples from the PCBC dataset [6, 7] to develop a stemness signature, which was then used to determine the mRNAsi value. There are 11 774 genes in the expression profiles derived from the mRNA expression-based signature. More information on the stemness indices and the flowchart that was used in the present research to produce the aforementioned indices are available on the following website: https://bioinformaticsfmrp.github.io/PanCanStem-Web. The OCLR algorithm was used to calculate the eigenvector weights for the RNA expression matrix, respectively. The mRNAsi stem cell index of breast cancer samples was calculated based on the obtained RNA expression data of breast cancer samples and the weights of eigenvectors calculated before. The stemness indices were utilized to stratify the breast cancer samples, which were then employed for the integrative analysis.

### 2.3. Calculating the Relationship between Breast Cancer Stemness Index and Clinical Features

In order to clarify the correlation between the stem index of breast cancer and the types and metastasis status, we compared mRNAsi of different subtypes (LumA, LumB, Basal, Normal, Her2 subtypes) and metastasis (non-metastasis) status. Assessing mRNAsi differences between groups to see if our calculated breast cancer stem cell index correlates with clinical traits of the disease.

The stemness index was treated as an independent continuous covariate in the present research. Using a three-phase approach, we investigated the correlation between stemness indices and OS in breast cancer. Specifically, we utilized the univariate Cox proportional hazard regression to compute hazard ratios (HRs) for overall survival (OS). Some of the parameters included mRNAsi gender, metastatic status, tumor histology, age, and subgroup. The results of Cox univariate regression showed the clinical indicators of breast cancer associated with the patients' prognosis. In addition, patients were divided into high- and low-risk groups according to their mRNAsi levels, which were obtained utilizing the “cutp” module of the R package “survMisc” (https://cran.r-project.org/web/packages/survMisc) with default settings, and the differences in survival among subjects with elevated mRNAsi and those with reduced mRNAsi were assessed utilizing Kaplan–Meier (K–M) survival plots. Finally, only patients in the Basal subgroup were shown to have a statistically significant survival difference between those with higher mRNAsi and those with lower mRNAsi.

With the aid of the “createDataPartition” module of the R package “caret” (https://cran.r-project.org/web/packages/caret), we were able to divide the Basal subgroup dataset at random into two parts, namely, the 70 percent training set and the 30 percent validation set. We then utilized nondefault parameters for the “createDataPartition” module as follows: *P*=0.7 and list = FALSE. The Chi-square test for categorical variables and Kruskal–Wallis test for continuous variables were utilized to examine the distributions of clinical features across the training set as well as the validation set. In the training set, we divided the gene expression data into mRNA, lncRNA, and performed Cox univariate regression, respectively. Significantly related genes were selected, and then the correlation between their expression and the stem cell index of samples was calculated. Those with correlation coefficients cor >0.2 and correlation test *P*-value <0.05) were selected as candidate genes.

### 2.4. Lasso to Build the Best Multivariate COX Model

This step uses machine learning to further filter the candidate lncRNAs and mRNAs, to construct the best gene predicting panel in the Basal subtype. We calculated the lncRNA panel and mRNA panel risk score for each sample based on expression and multiple regression coefficients. The equation for determining risk scores is shown below:

(1) $$\begin{array}{c} \text{Riskscore}=\sum_{i=1}^{n}\beta i^{*}xi. \end{array}$$

The samples were divided into high index group and low index group according to the risk index (only 2 miRNAs were not screened by Lasso, they were divided into high expression group and low expression group according to their expression levels and drawn, respectively), Kaplan–Meier survival analysis was performed, and survival curves were drawn. Furthermore, based on the risk index of mRNA and lncRNA, ROC curves of three-year, five-year, and ten-year survival periods were drawn. We explored whether the models were accurate predictors based on the area under the curve (AUC) of a time-dependent receiver operating characteristic (ROC) study.

### 2.5. Assessment of Relationships between Stemness Indices and the Immune Landscape

By means of CIBERSORT (a deconvolution algorithm according to gene expression) (https://cibersort.stanford.edu/) [8], we calculated the relative abundance of the immune cells in the sample that was received. Using ESTIMATE [9], we calculated individual immunity scores to anticipate the level of infiltration of immune cells in each basal sample. The association between mRNAsi and immunological score was also analyzed.

### 2.6. CMap Predict mRNAsi-Related Drugs

The newly revised CMap (September 2017) [10] is a data-driven and systematic technique for uncovering associations among genes, chemicals, and biological circumstances, to screen for prospective substances that could target pathways associated with breast cancer stemness. Using the CMap database, a sum of 42080 perturbation factors were analyzed and 473647 reference signatures were generated. The CMap workflow consists of querying the CMap reference signature dataset (a LIST of DEGs associated with the biologic state of interest) using a pattern-matching algorithm. The scores fell within the range of −100 to 100. Molecular compounds are ordered on the basis of their proportion to produce the most similar as well as the most opposite compounds. The website https://clue.io provides the CMap data as well as relevant tools. The “lmFit” module of the R package “limma” was utilized to determine the DEGs between the Basal subgroup samples that had elevated mRNAsi and those with reduced mRNAsi on the basis of default settings [11]. A number of genes that had differential expression across Basal subgroup samples with elevated mRNAsi and reduced mRNAsi was compiled, and the topmost 300 genes (150 of which were upmodulated and 150 of which were downmodulated) were chosen for further investigation in the CMap database. Compounds having an enrichment score of ≤−95 were identified as promising chemotherapeutic drugs for the treatment of basal breast cancer.

### 2.7. Statistical Analysis

In the present research, all statistical analyses were conducted utilizing R (version: 3.4.1) (R Core Team, R Foundation for Statistical Computing, Vienna, Austria). In the case when using the default settings of the R package “gelnet,” the OCLR technique was applied successfully [12]. We calculated the *P* values for the correlations between stemness indices and the immune milieu utilizing Pearson's correlation coefficient tests, followed by multiple testing utilizing the BH technique. Statistical significance was reached when the value of *P* was less than 0.05.

## 3. Results

### 3.1. Breast Cancer Stemness Indices Predicated on mRNA Expression

**Figure 1:**
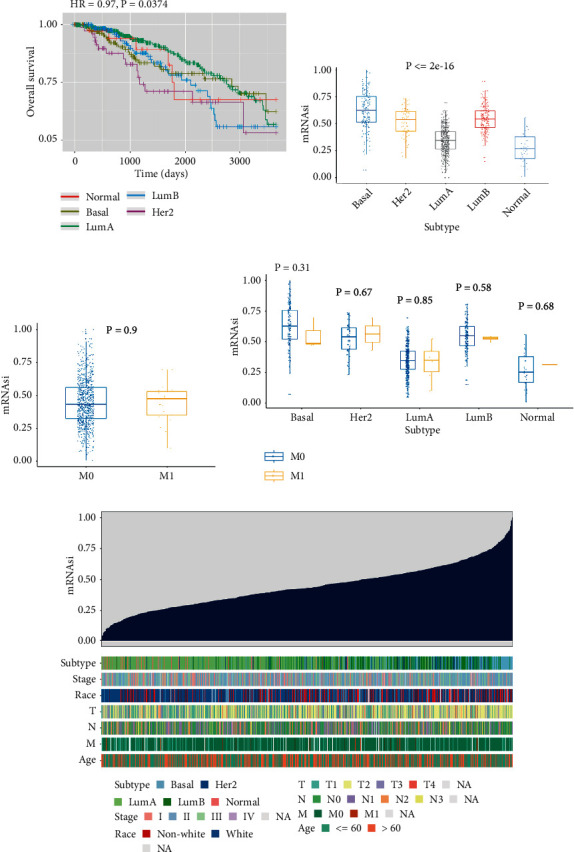
Survival for patients in the subtypes and clinical characteristics correlated with the mRNA expression‐based stemness index (mRNAsi) in breast cancer. (a) Patients' survival curves in distinct the subtypes for breast cancer. (b) Individual sample boxplots of mRNAsi classified by subtypes. (c) Individual mRNAsi boxplots classified by metastatic status. (d) Individual mRNAsi boxplots for each subtype classified by metastatic state. (e) Summary of the known associations between clinical and molecular characteristics (subtype, stage, race, pathologic TNM stage, and age) and mRNAsi in breast cancer.

On the basis of the analysis of the relationship between stemness index and survival of patients, there is an overall significant difference among each subtype (*P*=0.0374) (Figure 1(a)). We also found the Basal subtype has higher mRNAsi compared with others, and significant different mRNAsi among them (*P* < 0.05). Significant differences were not observed between metastatic (M0) and nonmetastatic (M1) samples, as well as in each subtype of M0 and M1 samples (Figures 1(b)–1(d)). Based on their specific mRNAsi values (ranging from low to high stemness index), we graded the breast cancer samples and searched for correlations with any demographic/molecular/clinical characteristics that were associated with either a higher or lower stemness index (Figure 1(e)).

### 3.2. Associations between Breast Cancer Stemness Indices and Clinical Outcome

**Figure 2:**
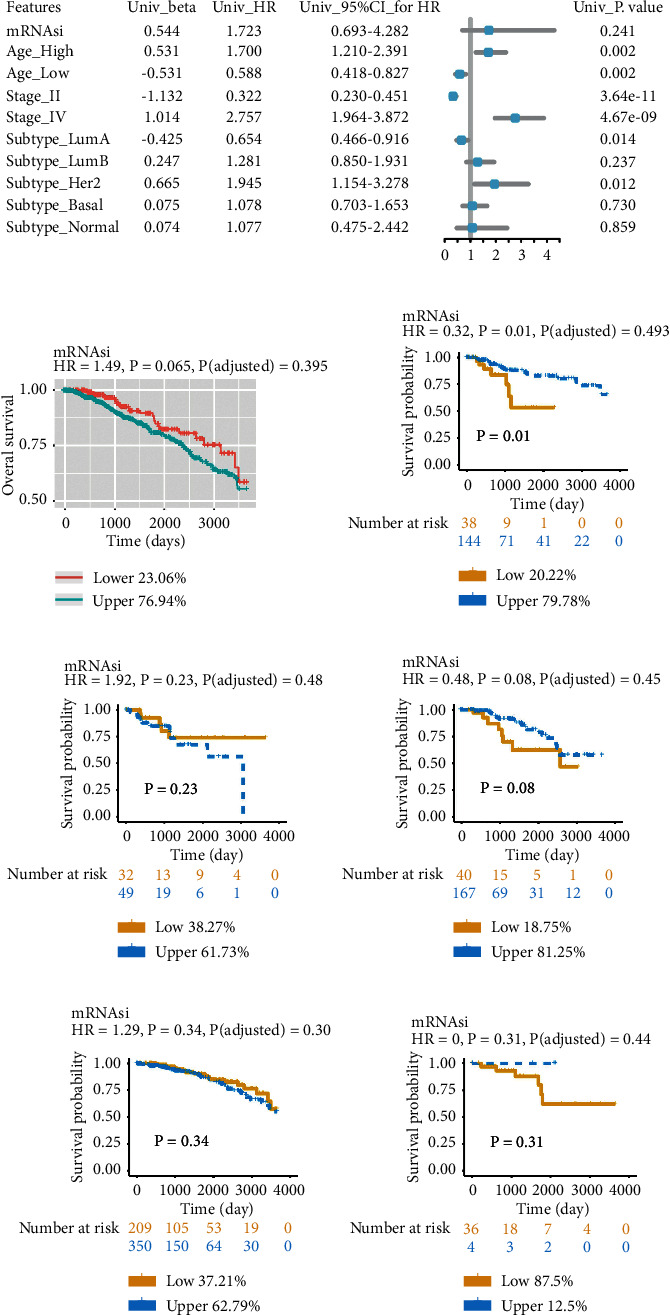
Association between OS of patients and mRNAsi. The K–M survival curves depict the OS rate for patients with low- and high mRNAsi, classified by the ideal threshold. (a) Cox regression studies of univariate data on clinical and molecular characteristics related to OS in MB patients. (b) K–M curves depicting the OS of all patients in breast cancer having a low or high mRNAsi. (c–g) K–M curves depicting the OS of patients respectively in subtype Basal (c), Her2 (d), LumB (e), LumA (f), and Normal (g).

Based on sample survival data, a univariate Cox regression analysis was conducted to test the association between clinical indicators and patients' overall survival (OS). The forest chart is displayed in Figure 2(a). To address the effect of mRNAsi on survival, we did the K–M plots by splitting all patients or each subtype sample into low and high mRNAsi groups (Figures 2(b)–2(g)). The results showed that mRNAsi exhibited a statistically significant impact on OS for Basal patients (HR, 0.32; *P*=0.01). Then, in basal patients, we conducted cox regression analysis for gene expression and survival. All genes with a significant survival relationship (*P* < 0.05) were subjected to correlation analysis with mRNAsi, and the genes with significant correlation (|cor| > 0.2, *P* < 0.05) were selected for statistical analyses. As a result, we got 2 miRNAs, 111 lncRNAs, and 389 mRNAs (Tables S1–S3).

**Figure 3:**
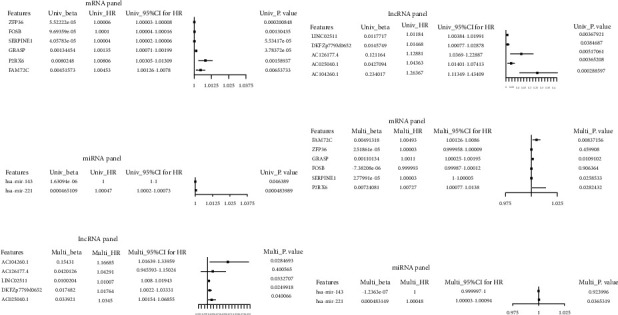
Univariate and multivariate cox regression beta index analysis in mRNA, lncRNA, and miRNA panel. (a–c) Predictive significance of each gene in mRNA, lncRNA, and miRNA panel using univariate cox regression beta index analysis. (d–f) Predictive significance of each gene in mRNA, lncRNA, and miRNA panel using Multivariate cox regression beta index analysis.

**Figure 4:**
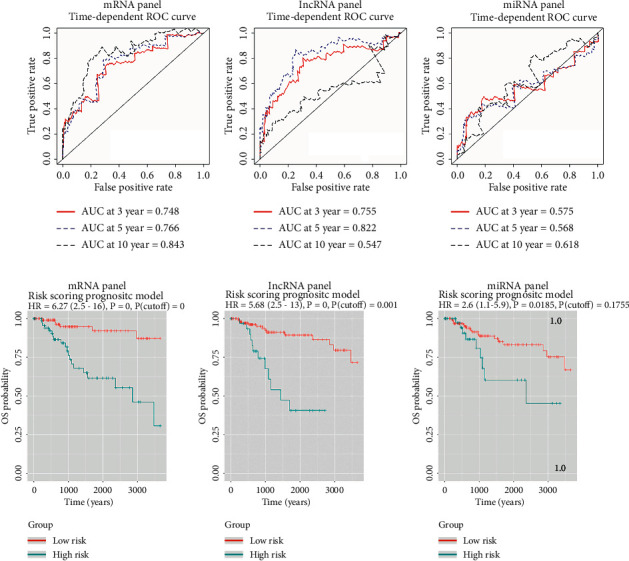
Predictive significance of the mRNA-, lncRNA-, and miRNA-based prognostic model in Basal subgroup patients. (a–c) Time‐dependent ROC curves illustrated the prediction power of the mRNA-, lncRNA-, and miRNA-based on the prognostic model in Basal patients. (d–f) K–M curves for Basal patients showed the prediction power of the mRNA-, lncRNA-, and miRNA-based on risk scoring prognostic model.

After the lasso machine learning on candidate 389 mRNA and 111 lncRNA, the mRNA panel (FAM72 C, ZFP36, GRASP, FOSB, SERPINE1, P2RX6), the lncRNA panel (AC104260.1, AC126177.4, LINC02511, DKFZp779M0652, AC025040.1), and the miRNA panel (hsa-mir-143 and hsa-mir-221) for Basal breast cancer were identified. Using univariate (Figures 3(a)–3(c)) and multivariate (Figures 3(d)–3(f)) cox regression beta index analysis, the expression of majority genes in each panel contributed to the prognosis of patients with Basal breast cancer (*P* < 0.05). Combined with gene expression and beta index of multivariate cox regression, we calculated the risk scores (RS) for each sample and then separated them into two groups in each panel, high RS and low RS. Based on the different RS level, three-year, five-year, and ten-year survival ROC curves were drawn for each panel (Figures 4(a)–4(c)). The prediction performance of the prognostic model was evaluated by computing the AUC of the ROC curves. With regard to the mRNA set, the AUCs concerning the 6‐mRNA biomarker prognostic model were 0.748, 0.766, and 0.843 for the 3‐, 5‐, and 10‐year survival times (Figure 4(a)). In the lncRNA set, AUCs concerning the 5‐lncRNA biomarker prognostic model were 0.755, 0.822 and 0.547 for the 3‐, 5‐, and 10‐year survival times (Figure 4(b)). For the 2 miRNA-based prognostic model, the AUCs were 0.575, 0.568, and 0.618 for the 3‐, 5‐, and 10‐year survival (Figure 4(c)). Then, the K–M plot was produced between low and high-risk score groups. The results showed that the risk model of mRNA, lncRNA, and miRNA panels were all significantly related to the survival of Basal patients (Figures 4(d)–4(f)). Taken together, mRNAsi-related genes were independent factors affecting the prognosis of Basal breast cancer.

### 3.3. Relationship between Stemness Indices and the Immune Milieu

**Figure 5:**
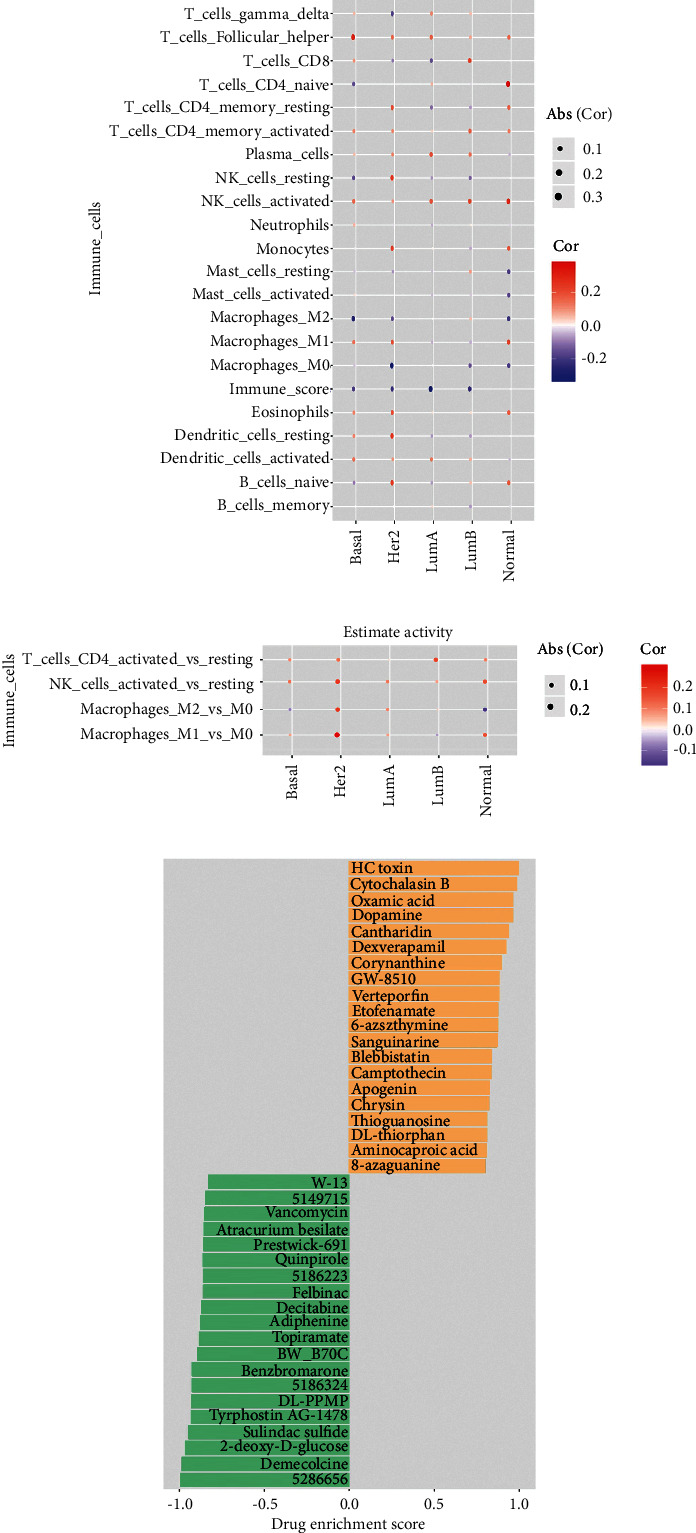
Relationships between stemness indices and the immune milieu in Basal patients, as well as the enrichment score for each compound (perturbagen) from the CMap for the profile of differently expressed genes in Basal patients with low- and high mRNAsi. (a) Associations between mRNAsi and CIBERSORT estimations of immune cell subgroup proportions are shown in the bar plot. (b) Associations between mRNAsi and estimated immune cell activity are shown in bar plots. Estimated immune cell activity is presented as differences between the proportions of active and resting groups. The correlations are included for macrophages, NK cells, and CD4 + T cells. (c) The top 40 enrichment scores for each chemical (perturbagen) obtained from the CMap.

We evaluated associations between specific kinds of immune cells and mRNAsi in order to better understand the relationships between stemness in Basal patients and the tumor immune milieu in the present study. CIBERSORT was used to calculate the relative abundance of immune cells in each sample based on the expression profile data of the sample (Supplementary Figure S1), and the corresponding immune index of each sample was obtained from ESTIMATE. Combining the relative abundance and immune score of the immune cells of each subtype sample, the relationships between mRNAsi index and immune cell, four immune cell activation status, and immune score in different subtypes were investigated. Out of all other subtypes of breast cancer, Her2 enrichment and Normal subtypes have higher immune activity, while the LumA subtype has lower immune activity. Among them, Basal subtype mRNAsi has a high positive correlation with the activation status of T cells and NK immune cells and a negative correlation with the resting or naive immune cells (Figures 5(a)–5(b)).

### 3.4. Analysis of the Connectivity Map Reveals New Potential Drugs that Target the Basal Stemness Signature

For the purpose of developing efficacious drugs that can target the pathways correlated with Basal stemness, we utilized mRNA expression signatures to query the connectivity map (CMap) database, followed by the analysis of differential expression in low or high mRNAsi values on breast cancer subgroups. A total of 1,308 potential drugs were obtained, of which the top ten most relevantly positive-regulated drugs were HC toxin, cytochalasin B, dopamine, oxamic acid, cantharidin, dexverapamil, corynanthine, GW-8510, verteporfin, and etofenamate. The top ten most relevantly negative-regulated drugs were 5286656, demecolcine, 2-deoxy-D-glucose, sulindac sulfide, tyrphostin AG-1478, DL-PPMP, 5186324, benzbromarone, BW-B70 C, and topiramate (Figure 5(c)).

## 4. Discussion

Using a stemness index model-based OCLR machine-learning algorithm, we calculated the stemness index of breast cancer samples in the TCGA database. With the aid of the stemness index, we compared the differences in stemness characteristics of distinct breast cancer subtypes and analyzed the association between breast cancer stemness and patient survival, as well as tumor immune invasion. We found that the stemness of different subtypes is significantly different. Although the survival time of patients within different subtypes is significantly different, we found no substantial association between the stemness index and the overall survival of patients in all breast cancer samples. Stemness also has no significant correlation with the patient's stage or metastasis. Then, we stratified the patients according to the subtype and found that the tumor stemness index in the Basal subtype is relatively high. At the same time, there are two groups of patients with low and high stemness only in the Basal subtype, which have significant differences in overall survival. Patients with high stemness have a longer overall survival, and patients with low stemness have a short overall survival. These suggest that the Basal subtype has higher internal heterogeneity and complexity in tumor stemness than other subtypes.

Stemness refers to cells with the capacity for self-renewal and differentiation, while tumor cells lose their original cellular characteristics during progression and alienate into poorly differentiated and highly proliferating cells, somehow similar to normal stem cells. It is generally believed that these stem cells with elevated stemness have a high likelihood of being migrated to distant organs due to high proliferation and invasion, which will result in an unfavorable prognosis of patients [13, 14]. However, our study found that higher stemness tumors are not as malignant. The breast stemness index of each subtype in breast cancer is not significantly correlated with the presence or absence of distant metastasis of tumors, and the prognosis of patients with high stemness in Basal is better. This is contrary to the oncogenic dedifferentiation in most malignant tumors, which tends to be stem-like. In fact, the association between high dryness and a good prognosis is not identified in all breast tumors. The stemness value of the Basal subtype is relatively high, although we know that the prognosis of patients with basal subtype is usually not good. To be noted, the two groups of high and low stemness we found here are stratified in Basal. This difference between high and low can only suggest that there are different types of Basal subtypes, and the stem cell genes signature could be applied in this classification. Then, we found that this stemness signature is actually related to tumor immune cell infiltration. The prognosis of the patient may be due to the difference in infiltrated immune cells.

With respect to the Basal subtype, we found that the stemness index was substantially correlated with the status of tumor immune infiltration. A strong positive association was observed between the ratio of stemness index and follicular helper T cells, also known as antigen-experienced CD4 + T cells. Moreover, the stemness index was significantly correlated with immune cells participating in tumor killing, such as CD8 + T cells, macrophage M1 type, activated NK, DC, and CD4 + memory T cells, while being negatively correlated with macrophage M2 type and naïve B and CD4 + T cells. This suggests that tumors with a high stemness index tend to trigger activated immune cells infiltration, which has the tumor-killing effect. This result also explains why we found that patients with higher stemness have a better prognosis than patients with lower stemness. Our findings show that the differentiation of M1 and M2 is significantly related to the stemness of the tumor. Patients with high tumor stemness have a high proportion of M1 infiltration, long overall survival, and patients with low tumor dryness have a high M2 infiltration ratio and poor prognosis. This is completely in line with the findings of earlier studies on tumor immunity [15–17].

Comparing the two groups of Basal subtypes with high and low stemness, we first identified the related mRNA and lncRNA and then screened and constructed a prognostic model based on 6 mRNA or 5 lncRNA, which can be a very good predictor for the patient's overall survival. It indicates that the expression of genes in this model is related to the tumor's stemness, and it can be associated with patients' prognoses. The clinical examination of these genes may be utilized to anticipate the patients' prognoses. Among these genes, ZFP36 is negatively correlated with drug resistance and proliferation [18]. FOSB is a transcription factor that affects tumor differentiation, proliferation, and metastasis in breast cancer [19]. SERPINE1 affects metastasis by affecting EGFR signaling [20]. To our knowledge, these lncRNAs have not been reported to be associated with breast cancer. The genes identified in these prognostic models can be used as new molecular markers for Basal subtyping of breast cancer.

We used the CMap database to analyze the differentially expressed genes of two groups of patients with high and low stemness whose prognosis is significantly different in the Basal subtype and obtained some drug compounds that can respond to these gene expression changes. Most of these compounds that are positively related to stemness are drugs that inhibit tumor metastasis and progression [21–25], indicating that these patients with high stemness are under the effective control of tumor metastasis and progression. Compounds that are negatively related to stemness are generally not used for tumor therapy, indicating that patients with low stemness need more effective tumor drug treatment to inhibit tumor progression.

Of course, this study also has some limitations. First, most of the samples in this study are Caucasian and African American (69.3%), so whether our results are also applicable to large sample data of other populations needs more data to support. Second, regarding the genes involved in our prognosis model, we only speculate that the function of these genes is associated with the occurrence and progression of breast cancer, and we need to add more experimental evidence to prove their molecular mechanism. Third, although we can explain why high-stemness tumor tissue has a better prognosis in the perspective of immune infiltration, more single-cell breast cancer data are still needed to confirm that stem-like cells in breast tumor tissues will induce or recruit more activated immune cells.

## 5. Conclusions

The present research is the first to refine the concept of tumor stem cell index into different subtypes of breast cancer. Among the subtypes, Basal is the one with the most closely related stem cell index and survival. We identified a stratification of Basal subtypes that are not only related to the stemness but also the prognosis and built a 6 mRNA-based or 5 lncRNA-based prognostic models for patients' overall survival. Further tumor immune infiltration and drug analysis confirmed that the two groups have different immune microenvironments and that different tumor drugs should be applied for their treatment. The classification signature in the present research might be used to improve individualized prediction of the prognosis of basal breast cancer and serve as a promising biomarker for basal breast cancer prognosis and responsiveness to differentiation treatments in clinical practice.

## Figures and Tables

**Table 1:** Clinicopathological characteristics in each subtype of breast cancer.

Items	Level	Subtype				
		Basal	Her2	LumA	LumB	Normal
N		183	81	559	208	40
Race (%)	Nonwhite	71 (40.3)	33 (46.5)	82 (15.8)	46 (25.8)	11 (28.2)
	White	105 (59.7)	38 (53.5)	438 (84.2)	132 (74.2)	28 (71.8)

Age (median (IQR))		54.00 (48.00, 62.50)	57.00 (50.00, 64.00)	61.00 (49.00, 69.00)	58.50 (50.00, 68.25)	53.00 (46.00, 62.50)
Pathologic_T (%)	T1	37 (20.3)	17 (21.0)	175 (31.4)	37 (17.8)	11 (27.5)
	T2	121 (66.5)	52 (64.2)	291 (52.2)	135 (64.9)	18 (45.0)
	T3	18 (9.9)	7 (8.6)	74 (13.3)	24 (11.5)	11 (27.5)
	T4	6 (3.3)	5 (6.2)	17 (3.1)	12 (5.8)	0 (0.0)

Pathologic_N (%)	N0	117 (63.9)	29 (37.7)	255 (46.5)	84 (41.2)	21 (53.8)
	N1	46 (25.1)	28 (36.4)	194 (35.4)	77 (37.7)	8 (20.5)
	N2	14 (7.7)	11 (14.3)	56 (10.2)	33 (16.2)	4 (10.3)
	N3	6 (3.3)	9 (11.7)	43 (7.8)	10 (4.9)	6 (15.4)

Pathologic_M (%)	M0	159 (98.1)	70 (95.9)	449 (98.0)	178 (97.3)	33 (97.1)
	M1	3 (1.9)	3 (4.1)	9 (2.0)	5 (2.7)	1 (2.9)

Pathologic_stage (%)	Stage I-II	151 (83.9)	56 (70.9)	410 (75.4)	142 (68.9)	28 (70.0)
	Stage III-IV	29 (16.1)	23 (29.1)	134 (24.6)	64 (31.1)	12 (30.0)

ER_status_by_IHC (%)	Negative	157 (89.2)	47 (62.7)	11 (2.0)	3 (1.5)	14 (37.8)
	Positive	19 (10.8)	28 (37.3)	526 (98.0)	194 (98.5)	23 (62.2)

PR_status_by_IHC (%)	Negative	163 (93.7)	62 (80.5)	54 (10.1)	39 (19.8)	17 (45.9)
	Positive	11 (6.3)	15 (19.5)	480 (89.9)	158 (80.2)	20 (54.1)

HER2_status_by_IHC (%)	Negative	114 (92.7)	13 (20.6)	304 (82.8)	99 (74.4)	20 (87.0)
	Positive	9 (7.3)	50 (79.4)	63 (17.2)	34 (25.6)	3 (13.0)

## Data Availability

The data used to support the findings of this study are included within the article.

## Supplementary Materials

Supplementary Materials Figure legend S1. The relative abundance of immune cells in each sample based on the expression profile data of the sample was calculated by CIBERSORT. Table S1. Correlation analysis with mRNAsi and miRNAs. Table S2. Correlation analysis with mRNAsi and lncRNAs. Table S3. Correlation analysis with mRNAsi and mRNAs.
