# Supplementary material for: Analysis of Stemness and Prognosis of Subtypes in Breast Cancer Using the Transcriptome Sequencing Data
Source: J Oncol. 2022 Mar 9;2022:5694033. doi: 10.1155/2022/5694033 (PMC8926471; doi:10.1155/2022/5694033)
Supplement: Supplementary Materials — Figure legend S1. The relative abundance of immune cells in each sample based on the expression profile data of the sample was calculated by CIBERSORT. Table S1. Correlation analysis with mRNAsi and miRNAs. Table S2. Correlation analysis with mRNAsi and lncRNAs. Table S3. Correlation analysis with mRNAsi and mRNAs. [file 5694033.f1.zip › 5694033.f1/Revised Supplementary Figure 1.pdf]

CIBERSORT

Input Sample	B cells naive	B cells memory	Plasma cells	T cells CD8	T cells CD4 naive	T cells CD4 memory resting	T cells CD4 memory activated	T cells follicular helper	T cells regulatory (Tregs)	T cells gamma delta	NK cells resting	NK cells activated	Monocytes	Macrophages M0	Macrophages M1	Macrophages M2	Dendritic cells resting	Dendritic cells activated	Mast cells resting	Mast cells activated	Eosinophils	Neutrophils	P-value	Pearson Correlation	RMSE
TCGA-3C-AAAU-01	0.08	0	0.042	0.015	0	0.153	0	0.03	0.089	0	0.009	0.01	0.021	0.11	0.033	0.365	0	0	0.043	0	0	0	0.910	-0.024	1.101
TCGA-3C-AALI-01	0.093	0	0.015	0.097	0	0.262	0	0.035	0.06	0	0	0.018	0.025	0.216	0.106	0.013	0	0	0.061	0	0	0	0.020	0.225	1.002
TCGA-3C-AALJ-01	0.07	0	0.017	0.044	0.001	0.193	0	0.001	0.053	0	0.109	0	0.04	0.367	0.058	0.047	0	0	0	0	0	0	0.010	0.373	0.936
TCGA-3C-AALK-01	0.077	0	0.103	0.045	0	0.284	0	0	0.048	0	0.029	0.004	0.024	0	0.081	0.182	0.027	0.009	0.087	0	0	0	0.240	0.067	1.048
TCGA-4H-AAAK-01	0.081	0	0.039	0.028	0	0.262	0	0.017	0.009	0	0.025	0	0.057	0.071	0.053	0.232	0	0.01	0.115	0	0	0	0.210	0.076	1.046
TCGA-5L-AAT0-01	0.105	0	0.007	0.097	0	0.29	0	0.007	0.058	0	0.04	0	0.015	0.049	0.077	0.192	0	0	0.063	0	0	0	0.040	0.173	1.019
TCGA-5L-AAT1-01	0.058	0	0.029	0.164	0	0.177	0	0.037	0.064	0	0.057	0	0.023	0	0.105	0.254	0.002	0	0.032	0	0	0	0.010	0.316	0.957
TCGA-5T-A9QA-01	0.117	0	0.115	0.046	0	0.099	0	0	0.067	0	0	0.059	0	0.247	0.011	0.214	0	0	0.024	0	0	0	0.720	-0.002	1.094
TCGA-A1-A0SB-01	0.045	0	0.004	0	0	0.34	0	0.107	0	0	0	0.085	0.04	0.002	0	0.201	0	0.085	0.082	0	0.009	0	1.000	-0.055	1.112
TCGA-A1-A0SD-01	0.091	0	0.055	0	0	0.31	0	0.022	0	0	0.025	0.007	0.008	0.074	0.126	0.2	0	0	0.082	0	0	0	0.580	0.018	1.078
TCGA-A1-A0SE-01	0.121	0	0.06	0.088	0	0.333	0	0	0.003	0	0.032	0	0.023	0	0.044	0.165	0.023	0.006	0.102	0	0	0	0.430	0.028	1.069
TCGA-A1-A0SF-01	0.09	0	0.139	0.131	0	0.359	0.01	0	0.002	0	0.036	0	0.043	0.033	0.048	0.04	0.003	0	0.065	0	0	0	0.050	0.136	1.033
TCGA-A1-A0SG-01	0.209	0.061	0.012	0.054	0	0.198	0	0	0	0	0.014	0	0.008	0.014	0.046	0.246	0.017	0	0.12	0	0	0	0.070	0.108	1.039
TCGA-A1-A0SH-01	0.06	0	0.039	0.01	0	0.236	0	0	0	0	0.022	0	0.006	0.107	0.03	0.374	0	0	0.113	0	0	0.003	0.220	0.073	1.063
TCGA-A1-A0SI-01	0.055	0	0.008	0	0	0.234	0	0.055	0.024	0	0	0.051	0.016	0.303	0.105	0.072	0	0	0.079	0	0	0	0.020	0.222	1.008
TCGA-A1-A0SJ-01	0.122	0	0.093	0.007	0	0.373	0	0	0	0	0.049	0	0.003	0.076	0.064	0.127	0.043	0	0.042	0	0	0	0.600	0.015	1.071
TCGA-A1-A0SK-01	0	0.016	0.033	0	0	0.477	0	0.222	0.002	0	0	0	0.044	0.198	0.008	0	0	0	0	0	0	0	0.720	-0.001	1.133
TCGA-A1-A0SM-01	0	0	0.117	0	0	0.18	0	0	0	0	0.011	0.049	0.016	0.05	0.005	0.44	0	0.017	0.11	0	0	0.006	0.310	0.053	1.071
TCGA-A1-A0SN-01	0.007	0	0.064	0.028	0	0.303	0.002	0	0.067	0	0.018	0.013	0.018	0.233	0.049	0.07	0.005	0	0.123	0	0	0	0.040	0.149	1.028
TCGA-A1-A0SO-01	0.095	0	0.072	0.015	0	0.286	0.006	0.025	0.003	0	0.043	0	0.009	0.178	0.091	0.177	0	0	0	0	0	0	0.660	0.006	1.088
TCGA-A1-A0SP-01	0.072	0	0.044	0.018	0	0.26	0	0.007	0	0	0.011	0.02	0.038	0.254	0.054	0.137	0.053	0.01	0.023	0	0	0	0.020	0.244	0.991
TCGA-A1-A0SQ-01	0.01	0	0.219	0.018	0	0.335	0	0.024	0	0	0.022	0.004	0.069	0.056	0	0.128	0	0	0.111	0	0.004	0	0.770	-0.010	1.070
TCGA-A2-A04N-01	0.156	0	0.062	0.027	0	0.177	0	0.024	0.002	0	0.02	0	0	0.108	0.033	0.289	0	0	0.102	0	0	0	0.240	0.065	1.054
TCGA-A2-A04P-01	0.11	0	0.058	0.044	0	0.321	0	0.041	0.031	0	0.037	0	0.016	0	0.116	0.15	0	0.067	0.009	0	0	0	0.040	0.190	1.016
TCGA-A2-A04Q-01	0.076	0	0.053	0.104	0	0.225	0.03	0.031	0.041	0	0.084	0	0.015	0.12	0.096	0.089	0.008	0.014	0.013	0	0	0	0.010	0.392	0.920
TCGA-A2-A04R-01	0.025	0	0.085	0	0	0.203	0	0.011	0.044	0	0.013	0.026	0.009	0.473	0	0	0	0.008	0.103	0	0	0	0.480	0.023	1.122
TCGA-A2-A04T-01	0.114	0	0.011	0.018	0	0.25	0	0.103	0.001	0	0	0.057	0.025	0.157	0.083	0.131	0.001	0.024	0.024	0	0	0	0.020	0.229	0.995
TCGA-A2-A04U-01	0.161	0	0.087	0.028	0	0.056	0	0.061	0.044	0	0	0.005	0	0.275	0.036	0.233	0	0.009	0.006	0	0	0	0.070	0.111	1.056
TCGA-A2-A04V-01	0.014	0	0	0.019	0	0.184	0	0.008	0.066	0	0	0.081	0.044	0.274	0.018	0.173	0	0	0.119	0	0	0	0.020	0.236	1.000
TCGA-A2-A04W-01	0.114	0	0.129	0.017	0	0.08	0	0.008	0.029	0	0	0.014	0	0.353	0.026	0.192	0	0	0.038	0	0	0	0.060	0.131	1.056
TCGA-A2-A04X-01	0.022	0	0.057	0.011	0	0.343	0	0.025	0.006	0	0.021	0.029	0.029	0.126	0.093	0.123	0	0.034	0.082	0	0	0	0.010	0.270	0.975
TCGA-A2-A04Y-01	0.055	0	0.083	0.048	0	0.325	0	0	0.002	0	0	0.015	0.034	0.048	0.094	0.277	0	0.004	0.005	0	0.01	0	0.040	0.177	1.016
TCGA-A2-A0CK-01	0.101	0	0.022	0.1	0	0.294	0	0	0.042	0	0.009	0	0.033	0	0.056	0.306	0	0	0.037	0	0	0	0.200	0.078	1.058
TCGA-A2-A0CL-01	0.082	0	0.015	0.053	0	0.457	0.002	0	0.001	0	0.093	0	0.053	0.029	0.131	0.066	0	0	0.018	0	0	0	0.010	0.435	0.902
TCGA-A2-A0CM-01	0.019	0	0	0.017	0	0.321	0.018	0.061	0.068	0	0.021	0.02	0.027	0.197	0.147	0.062	0	0	0.022	0	0	0	0.010	0.308	0.968
TCGA-A2-A0CO-01	0.171	0	0.001	0.101	0	0.386	0	0	0.023	0	0.053	0	0.035	0.016	0.085	0.124	0	0.001	0.003	0	0	0	0.050	0.145	1.045
TCGA-A2-A0CP-01	0.029	0	0.088	0.058	0	0.213	0	0	0	0	0.007	0.014	0.022	0.016	0.055	0.253	0	0	0.245	0	0	0	0.070	0.109	1.038
TCGA-A2-A0CQ-01	0.043	0	0	0.027	0	0.224	0	0.04	0.024	0	0	0.006	0.134	0	0.024	0.433	0	0.008	0.037	0	0	0	0.200	0.078	1.067
TCGA-A2-A0CR-01	0.081	0	0.009	0.194	0	0.251	0	0.022	0.077	0	0	0.027	0.026	0.098	0.081	0.1	0.003	0	0.031	0	0	0	0.000	0.526	0.851
TCGA-A2-A0CS-01	0.031	0	0	0.016	0	0.164	0	0.019	0.034	0	0.017	0	0.022	0	0	0.221	0.217	0.114	0.143	0	0	0.004	0.050	0.139	1.028
TCGA-A2-A0CT-01	0.124	0	0.054	0.002	0	0.149	0	0.023	0	0	0	0.024	0	0.014	0.045	0.341	0.042	0	0.182	0	0	0	0.350	0.044	1.064
TCGA-A2-A0CU-01	0.121	0	0.055	0.049	0	0.167	0	0.033	0.035	0	0.009	0.019	0.059	0	0.074	0.276	0	0	0.1	0	0	0	0.640	0.011	1.070
TCGA-A2-A0CV-01	0.097	0	0.019	0.084	0	0.325	0	0.016	0.01	0	0.004	0	0.068	0	0.075	0.263	0.018	0.008	0.014	0	0	0	0.220	0.069	1.062
TCGA-A2-A0CW-01	0.089	0	0.036	0.046	0	0.305	0.015	0.022	0.039	0	0.059	0	0.009	0.092	0.119	0.121	0	0.018	0.03	0	0	0	0.070	0.121	1.044
TCGA-A2-A0CY-01	0.111	0	0.016	0	0	0.189	0	0.034	0.062	0	0.07	0	0.024	0.461	0.008	0	0	0	0.025	0	0	0	0.060	0.127	1.072
TCGA-A2-A0CZ-01	0.052	0	0.062	0.015	0	0.163	0	0.004	0.012	0	0.001	0.015	0	0.406	0.058	0.089	0	0	0.124	0	0	0	0.000	0.545	0.839
TCGA-A2-A0D0-01	0.078	0	0	0.09	0	0.198	0.012	0.072	0.026	0	0.056	0.004	0.008	0.196	0.085	0.151	0.005	0	0.019	0	0	0	0.040	0.164	1.028
TCGA-A2-A0D1-01	0.102	0	0.139	0.067	0	0.142	0	0.04	0.044	0	0.026	0.001	0	0.268	0.045	0.125	0	0	0	0	0	0	0.430	0.030	1.078
TCGA-A2-A0D2-01	0.107	0	0	0.074	0	0.155	0	0	0.034	0	0.017	0	0.047	0.325	0.099	0.101	0	0	0.042	0	0	0	0.010	0.290	0.977

TCGA-A2-A0D3-01	0.097	0	0.052	0.115	0	0.409	0	0.014	0	0	0	0.01	0.014	0	0.061	0.187	0	0.032	0.009	0	0	0	0.280	0.060	1.076
TCGA-A2-A0D4-01	0.086	0	0.046	0	0	0.227	0	0	0.028	0	0	0.021	0.013	0.123	0.021	0.269	0	0	0.168	0	0	0	0.040	0.154	1.022
TCGA-A2-A0EM-01	0.017	0	0.023	0.046	0	0.204	0	0	0	0	0.011	0	0.095	0	0.012	0.353	0.055	0.089	0.094	0	0	0	0.630	0.012	1.081
TCGA-A2-A0EN-01	0.101	0	0	0.073	0	0.384	0	0.003	0.049	0	0.065	0	0.028	0.021	0.071	0.15	0.012	0	0.042	0	0	0	0.020	0.231	0.999
TCGA-A2-A0EO-01	0.075	0	0.042	0.056	0	0.315	0	0.019	0	0	0	0.019	0.024	0.082	0.083	0.202	0	0	0.083	0	0	0	0.070	0.116	1.038
TCGA-A2-A0EP-01	0.093	0	0	0.033	0	0.213	0.013	0.033	0.017	0	0.035	0	0.05	0.319	0.016	0.128	0.015	0.023	0.011	0	0	0	0.000	0.513	0.859
TCGA-A2-A0EQ-01	0.079	0	0.057	0.011	0	0.298	0.008	0.028	0.042	0	0.036	0.018	0	0.2	0.127	0.064	0	0	0.031	0	0	0	0.010	0.394	0.920
TCGA-A2-A0ER-01	0.035	0	0.145	0	0	0.361	0	0.014	0	0	0.009	0.019	0.018	0.032	0.03	0.209	0.007	0.015	0.107	0	0	0	0.490	0.022	1.062
TCGA-A2-A0ES-01	0.132	0	0.03	0	0	0.405	0	0	0	0	0.003	0.006	0.015	0	0.049	0.291	0.029	0.013	0.027	0	0	0	0.260	0.062	1.063
TCGA-A2-A0ET-01	0.106	0	0.018	0.038	0	0.347	0	0.019	0.016	0	0.024	0.008	0.044	0	0.046	0.277	0.056	0.002	0	0	0	0	0.770	-0.011	1.093
TCGA-A2-A0EU-01	0.063	0	0.008	0.008	0	0.309	0	0.025	0	0	0.002	0.014	0	0.146	0.08	0.104	0.016	0.038	0.186	0	0	0.001	0.370	0.040	1.072
TCGA-A2-A0EV-01	0.117	0	0.039	0.012	0	0.227	0	0.021	0.023	0	0.015	0	0	0.306	0.024	0.145	0	0.026	0.045	0	0	0	0.030	0.206	1.012
TCGA-A2-A0EW-01	0.155	0	0.022	0.058	0	0.301	0	0.026	0	0	0	0.024	0.012	0	0.053	0.257	0.019	0.002	0.07	0	0	0	0.290	0.059	1.060
TCGA-A2-A0EX-01	0.157	0	0.031	0.047	0	0.287	0	0	0.027	0	0.033	0	0.013	0.153	0.033	0.154	0	0	0.065	0	0	0	0.890	-0.020	1.091
TCGA-A2-A0EY-01	0.112	0	0.013	0.03	0	0.311	0	0.013	0	0	0.057	0.008	0.039	0.041	0.087	0.187	0.014	0	0.088	0	0	0	0.280	0.060	1.059
TCGA-A2-A0ST-01	0.069	0.002	0.051	0.097	0	0.239	0.001	0.049	0.056	0	0.02	0.005	0.012	0.123	0.153	0.036	0.03	0.024	0.031	0	0	0	0.000	0.524	0.853
TCGA-A2-A0SU-01	0.123	0	0.012	0.056	0	0.324	0	0	0.032	0	0.01	0.009	0.041	0.117	0.032	0.169	0.005	0.017	0.053	0	0	0	0.370	0.041	1.069
TCGA-A2-A0SV-01	0.046	0	0	0	0	0.242	0.001	0.023	0.031	0	0.038	0	0.004	0.16	0.064	0.376	0	0	0.012	0	0	0.003	0.620	0.012	1.106
TCGA-A2-A0SW-01	0.071	0	0.219	0.068	0	0.224	0.004	0.008	0.031	0	0.014	0.016	0.023	0.086	0.072	0.116	0	0	0.048	0	0	0	0.010	0.292	0.959
TCGA-A2-A0SX-01	0.03	0	0.06	0.012	0	0.297	0	0	0.059	0	0.009	0	0.029	0.34	0.103	0.055	0	0	0.005	0	0	0	0.000	0.517	0.856
TCGA-A2-A0SY-01	0.151	0	0.071	0.064	0	0.303	0.002	0	0.002	0	0.027	0	0.034	0.025	0.065	0.166	0.011	0	0.08	0	0	0	0.040	0.160	1.015
TCGA-A2-A0T0-01	0.077	0	0	0.012	0	0.253	0	0.014	0.061	0	0.037	0.008	0.006	0.277	0.086	0.161	0	0	0	0.008	0	0	0.040	0.169	1.035
TCGA-A2-A0T1-01	0.05	0	0.065	0.021	0	0.25	0.004	0.002	0.059	0	0.009	0.028	0.027	0.221	0.093	0.135	0	0	0.036	0	0	0	0.010	0.325	0.952
TCGA-A2-A0T2-01	0.081	0	0.01	0.112	0	0.172	0.008	0.063	0.036	0	0.003	0.015	0.014	0.103	0.094	0.271	0	0	0.012	0	0	0.005	0.210	0.076	1.063
TCGA-A2-A0T3-01	0.131	0	0.039	0.03	0	0.212	0	0	0.004	0	0.061	0	0.006	0.327	0.054	0.124	0	0	0.011	0	0	0	0.060	0.127	1.053
TCGA-A2-A0T4-01	0.064	0	0.014	0.053	0	0.177	0	0.032	0.03	0	0.004	0.009	0	0.185	0.059	0.185	0	0.012	0.176	0	0	0	0.020	0.235	0.991
TCGA-A2-A0T5-01	0.092	0	0.026	0.019	0	0.165	0	0.017	0.019	0	0.018	0.001	0.032	0.115	0.038	0.33	0	0.088	0.039	0	0	0	0.600	0.015	1.086
TCGA-A2-A0T6-01	0.135	0	0.021	0.036	0	0.324	0	0.023	0	0	0.026	0	0.012	0.007	0.053	0.213	0.003	0.005	0.133	0.008	0	0	0.050	0.143	1.025
TCGA-A2-A0T7-01	0.091	0	0.015	0.038	0	0.325	0	0	0	0	0.027	0	0.005	0.108	0.025	0.301	0	0.029	0.037	0	0	0	0.070	0.112	1.046
TCGA-A2-A0YC-01	0.113	0	0.039	0.017	0	0.183	0	0.008	0.025	0	0.02	0.003	0.019	0.099	0.033	0.236	0.07	0.001	0.117	0	0	0.016	0.220	0.071	1.048
TCGA-A2-A0YD-01	0.081	0	0.006	0.026	0	0.305	0	0.018	0.011	0	0.038	0	0.016	0.074	0.071	0.271	0	0	0.076	0	0	0.008	0.130	0.092	1.049
TCGA-A2-A0YF-01	0.008	0	0.034	0	0	0.205	0	0.031	0.033	0	0.027	0	0	0.422	0	0.154	0.001	0	0.075	0	0.01	0	0.040	0.193	1.042
TCGA-A2-A0YG-01	0.102	0	0.015	0	0	0.301	0	0.013	0.031	0	0.036	0	0.005	0.184	0.062	0.199	0	0.002	0.051	0	0	0	0.410	0.032	1.078
TCGA-A2-A0YH-01	0.005	0	0.076	0.025	0	0.203	0	0	0.004	0	0	0.02	0.024	0.096	0.06	0.307	0	0.033	0.148	0	0	0	0.010	0.254	0.981
TCGA-A2-A0YI-01	0.088	0	0.04	0.032	0	0.288	0.006	0.002	0.012	0	0.031	0	0	0.154	0.064	0.257	0.003	0	0.022	0	0	0	0.040	0.161	1.025
TCGA-A2-A0YK-01	0.146	0	0.009	0.08	0	0.457	0.004	0.01	0	0	0.015	0	0.024	0.009	0.098	0.121	0	0	0.028	0	0	0	0.010	0.294	0.974
TCGA-A2-A0YL-01	0.116	0	0.01	0.076	0	0.411	0	0	0	0	0.006	0.001	0.015	0	0.089	0.179	0.027	0	0.07	0	0	0	0.130	0.091	1.057
TCGA-A2-A0YM-01	0.027	0	0	0.02	0	0.169	0	0.001	0	0	0.036	0	0.025	0.493	0.063	0.041	0	0.126	0	0	0	0	0.010	0.387	0.948
TCGA-A2-A0YT-01	0.05	0	0.099	0	0	0.261	0	0	0	0	0	0.058	0.043	0.007	0	0.207	0	0.119	0.151	0	0.006	0	0.610	0.014	1.065
TCGA-A2-A1FV-01	0.201	0	0.004	0	0	0.259	0	0.035	0	0	0	0.036	0.004	0	0.016	0.266	0	0	0.179	0	0	0	0.960	-0.035	1.097
TCGA-A2-A1FW-01	0.099	0	0.056	0.02	0	0.413	0	0	0	0	0.013	0.004	0.016	0.005	0.072	0.255	0	0.001	0.045	0	0	0	0.900	-0.023	1.096
TCGA-A2-A1FX-01	0.033	0	0	0.033	0	0.253	0	0.012	0	0	0.008	0	0.109	0	0.07	0.358	0	0	0.125	0	0	0	0.340	0.046	1.072
TCGA-A2-A1FZ-01	0.082	0	0.004	0.048	0	0.26	0	0.003	0.001	0	0.019	0	0.061	0.047	0.055	0.334	0.031	0	0.055	0	0	0	0.220	0.072	1.059
TCGA-A2-A1G0-01	0.129	0	0.095	0.048	0	0.332	0.003	0	0	0	0.015	0	0.026	0	0.041	0.224	0.061	0	0.026	0	0	0	0.210	0.074	1.048
TCGA-A2-A1G1-01	0.072	0	0.025	0.046	0	0.243	0.049	0.02	0.013	0	0.034	0	0.013	0.087	0.167	0.167	0.007	0.03	0.019	0	0	0.009	0.040	0.187	1.021
TCGA-A2-A1G4-01	0.087	0	0.102	0.047	0	0.275	0	0.046	0	0	0.063	0	0.04	0.025	0.027	0.257	0	0	0.03	0	0	0	0.780	-0.011	1.080
TCGA-A2-A1G6-01	0.004	0	0.096	0.114	0	0.27	0	0.014	0	0	0	0.001	0.108	0.024	0	0.186	0.017	0.048	0.105	0	0.013	0	0.210	0.074	1.045
TCGA-A2-A259-01	0.133	0	0	0.014	0	0.27	0.019	0	0.018	0	0.04	0	0.052	0.026	0.045	0.113	0.004	0.04	0.228	0	0	0	0.060	0.132	1.029
TCGA-A2-A25A-01	0.069	0	0.017	0.048	0	0.375	0	0	0	0	0.021	0	0.017	0	0.085	0.236	0.005	0.003	0.124	0	0	0	0.070	0.117	1.041
TCGA-A2-A25B-01	0.011	0	0.018	0	0	0.369	0	0.012	0.02	0	0	0.007	0.017	0.098	0.102	0.226	0	0.03	0.09	0	0	0	0.120	0.095	1.055
TCGA-A2-A25C-01	0.061	0	0.064	0	0	0.371	0	0	0	0	0	0.009	0.05	0.064	0	0.063	0	0.164	0.154	0	0.001	0	0.410	0.036	1.066
TCGA-A2-A25D-01	0.105	0	0	0.012	0	0.373	0.009	0.013	0.008	0	0.099	0	0.041	0.013	0.13	0.115	0	0	0.082	0	0	0	0.010	0.304	0.965
TCGA-A2-A25E-01	0.131	0	0.053	0	0	0.291	0.001	0.053	0	0	0.044	0	0.028	0.027	0.1	0.199	0.013	0.003	0.057	0	0.001	0	0.780	-0.011	1.083
TCGA-A2-A25F-01	0.051	0	0	0.026	0	0.433	0.036	0	0.001	0	0.032	0	0.014	0.172	0.097	0.114	0	0.005	0.021	0	0	0	0.000	0.518	0.856
TCGA-A2-A3KC-01	0.136	0	0.069	0.002	0	0.28	0	0.007	0	0	0.045	0	0.005	0.079	0.042	0.289	0.013	0	0.032	0	0	0	0.070	0.114	1.036
TCGA-A2-A3KD-01	0.117	0	0.108	0.028	0	0.265	0	0	0	0	0	0.027	0.031	0.03	0.029	0.309	0	0	0.055	0	0	0	0.370	0.041	1.059

TCGA-A2-A3XS-01	0.118	0	0	0.075	0	0.276	0	0.03	0.088	0	0.058	0	0.077	0.082	0.059	0.056	0	0	0.079	0	0	0	0.190	0.081	1.056
TCGA-A2-A3XT-01	0	0	0	0.024	0	0.138	0.012	0.004	0.033	0	0.025	0	0.006	0.554	0.052	0.127	0	0	0.024	0	0	0	0.010	0.404	0.946
TCGA-A2-A3XU-01	0.123	0	0.164	0.029	0	0.027	0	0.054	0.057	0	0	0.016	0	0.257	0.002	0.271	0	0	0	0	0	0	0.950	-0.028	1.113
TCGA-A2-A3XV-01	0.071	0	0.013	0.028	0	0.121	0	0.03	0.025	0	0.012	0.01	0.001	0.205	0.033	0.436	0	0	0.014	0	0	0	0.070	0.115	1.073
TCGA-A2-A3XW-01	0.038	0	0.058	0.036	0	0.144	0	0.012	0.085	0	0	0.05	0.031	0.173	0.014	0.207	0.061	0.012	0.079	0	0	0	0.040	0.189	1.007
TCGA-A2-A3XX-01	0.099	0	0.093	0.053	0	0.122	0.011	0.025	0.012	0	0.058	0	0	0.294	0.07	0.137	0	0	0.026	0	0	0	0.030	0.200	1.011
TCGA-A2-A3XY-01	0.062	0	0	0	0	0.234	0	0.045	0.017	0	0.062	0	0	0.342	0.143	0.081	0	0	0.015	0	0	0	0.000	0.505	0.863
TCGA-A2-A3XZ-01	0.194	0.04	0	0.059	0	0.257	0.005	0.035	0.074	0	0.033	0.004	0.019	0.026	0.052	0.166	0.006	0.013	0.018	0	0	0	0.010	0.353	0.939
TCGA-A2-A3Y0-01	0.011	0	0.013	0.106	0	0.23	0.001	0.076	0.104	0	0.012	0.065	0.018	0.137	0.094	0.109	0	0.008	0.016	0	0	0	0.010	0.278	0.981
TCGA-A2-A4RW-01	0.077	0	0.029	0.098	0	0.211	0	0	0.039	0	0.054	0	0.035	0	0.055	0.143	0	0	0.258	0	0	0	0.060	0.134	1.033
TCGA-A2-A4RX-01	0.115	0	0	0.05	0.014	0.219	0	0	0.058	0	0.081	0	0.026	0.294	0.04	0.095	0	0	0.009	0	0	0	0.020	0.227	1.005
TCGA-A2-A4RY-01	0.146	0	0.046	0.034	0	0.301	0	0.001	0	0	0.059	0	0.077	0.043	0.05	0.181	0	0	0.062	0	0	0	0.210	0.076	1.047
TCGA-A2-A4S0-01	0.097	0	0.117	0	0	0.175	0	0.016	0.019	0	0	0.051	0.018	0	0.009	0.386	0	0	0.111	0	0	0	0.640	0.009	1.073
TCGA-A2-A4S1-01	0.004	0	0	0	0	0.085	0	0	0.008	0	0.025	0.019	0	0.183	0.025	0.617	0	0	0.033	0	0	0	0.000	0.478	0.888
TCGA-A2-A4S2-01	0.094	0	0.014	0.043	0	0.293	0	0.006	0.033	0	0.063	0	0.034	0.21	0.05	0.114	0	0.012	0.035	0	0	0	0.050	0.138	1.035
TCGA-A2-A4S3-01	0.004	0	0.008	0	0	0.195	0.003	0.046	0.093	0	0.032	0.015	0.022	0.368	0.072	0.093	0	0	0.05	0	0	0	0.030	0.221	1.016
TCGA-A7-A0CD-01	0.099	0	0.069	0.028	0	0.172	0	0.012	0.029	0	0.027	0.002	0.006	0	0.057	0.27	0.014	0.006	0.209	0	0	0	0.680	0.005	1.073
TCGA-A7-A0CE-01	0.115	0	0.087	0.004	0	0.167	0	0	0.039	0	0.006	0.016	0	0.224	0.102	0.189	0	0.029	0.021	0	0	0	0.090	0.103	1.053
TCGA-A7-A0CE-11	0.083	0	0.106	0.04	0	0.21	0	0	0	0	0.043	0.008	0.046	0	0.017	0.392	0	0	0.055	0	0	0	0.040	0.153	1.020
TCGA-A7-A0CG-01	0.098	0	0.055	0.062	0	0.444	0.011	0	0	0	0.021	0	0.021	0.002	0.058	0.137	0.01	0.01	0.069	0	0	0	0.040	0.177	1.018
TCGA-A7-A0CH-01	0.115	0	0.033	0.004	0	0.246	0	0.017	0	0	0	0.016	0.006	0.097	0.036	0.333	0.004	0.04	0.054	0	0	0	0.790	-0.012	1.092
TCGA-A7-A0CJ-01	0.095	0	0.014	0.025	0	0.244	0.001	0.034	0.028	0	0.037	0	0.009	0.273	0.062	0.148	0.001	0.005	0.024	0	0	0	0.340	0.045	1.083
TCGA-A7-A0D9-01	0.085	0	0.12	0	0	0.156	0	0	0.031	0	0	0.026	0	0.228	0	0.282	0	0.038	0.034	0	0	0	0.410	0.035	1.082
TCGA-A7-A0D9-11	0.003	0	0.005	0	0	0.323	0	0.002	0.001	0	0.057	0.011	0.094	0.115	0.02	0.361	0.001	0	0	0	0.007	0	0.210	0.074	1.076
TCGA-A7-A0DA-01	0.132	0	0.011	0	0	0.199	0	0.019	0.036	0	0.048	0.014	0.015	0.427	0	0.046	0	0.039	0.015	0	0	0	0.040	0.170	1.047
TCGA-A7-A0DB-01	0	0	0.006	0	0	0.203	0	0.005	0.042	0	0	0.015	0.024	0.444	0	0.138	0.001	0	0.111	0	0.011	0	0.040	0.187	1.052
TCGA-A7-A0DB-11	0.031	0	0.001	0	0	0.173	0	0.003	0.001	0	0	0.071	0.061	0	0	0.571	0	0	0.088	0	0	0	0.010	0.251	1.002
TCGA-A7-A0DC-01	0.171	0	0.12	0	0	0.199	0	0	0.003	0	0	0.056	0	0.201	0	0.149	0	0	0.099	0	0	0	0.990	-0.045	1.096
TCGA-A7-A0DC-11	0.011	0	0.052	0.046	0	0.299	0	0	0	0	0.004	0.057	0.077	0	0.02	0.293	0	0.01	0.131	0	0	0	0.410	0.032	1.070
TCGA-A7-A13D-01	0.084	0	0	0	0	0.082	0	0.058	0.033	0	0	0.018	0	0.667	0	0	0	0.058	0	0	0	0	0.060	0.132	1.140
TCGA-A7-A13E-01	0	0.002	0.022	0	0	0.348	0	0.028	0.058	0	0.047	0	0.037	0.422	0.002	0	0	0	0.034	0	0	0	0.210	0.075	1.099
TCGA-A7-A13E-11	0.044	0	0.041	0	0	0.239	0	0	0	0	0	0.085	0.099	0	0.018	0.353	0	0	0.11	0	0.011	0	0.090	0.104	1.044
TCGA-A7-A13F-01	0.063	0	0.045	0	0	0.136	0	0.016	0	0	0.014	0.002	0	0.006	0.011	0.486	0.116	0.02	0.069	0	0	0.015	0.190	0.082	1.068
TCGA-A7-A13F-11	0.11	0	0.092	0.11	0	0.223	0	0	0	0	0	0.025	0.059	0	0.005	0.283	0	0.011	0.082	0	0	0	0.310	0.056	1.053
TCGA-A7-A13G-01	0.022	0	0.09	0	0.042	0.295	0	0	0	0	0.045	0	0.044	0	0.006	0.37	0.052	0.005	0.029	0	0	0	0.820	-0.015	1.090
TCGA-A7-A13G-11	0.014	0	0.003	0	0	0.436	0	0.004	0.002	0	0.05	0.053	0.09	0	0.014	0.231	0	0.002	0.089	0	0.013	0	0.760	-0.007	1.099
TCGA-A7-A13H-01	0.043	0	0.017	0.021	0	0.115	0	0.003	0.057	0	0.002	0	0.005	0.519	0.057	0.098	0	0	0.063	0	0	0	0.000	0.521	0.857
TCGA-A7-A26E-01	0.056	0	0.038	0	0	0.192	0	0	0	0	0	0.022	0.153	0	0.017	0.4	0	0.011	0.084	0	0.028	0	0.630	0.012	1.086
TCGA-A7-A26F-01	0.027	0	0.017	0	0	0.459	0	0	0	0	0.102	0	0.034	0.061	0.037	0.169	0	0.071	0	0.009	0.01	0.004	0.190	0.080	1.066
TCGA-A7-A26G-01	0.093	0	0.101	0	0	0.336	0	0	0	0	0.024	0	0.016	0.15	0.054	0.12	0	0.001	0.104	0	0	0	0.040	0.154	1.016
TCGA-A7-A26H-01	0.086	0	0.014	0.029	0	0.275	0	0.008	0	0	0.007	0.027	0.019	0.261	0.059	0.158	0	0	0.059	0	0	0	0.070	0.124	1.047
TCGA-A7-A26I-01	0	0	0.013	0	0	0.432	0	0.023	0.038	0	0.055	0	0.025	0.127	0	0.233	0	0	0.055	0	0	0	0.180	0.086	1.066
TCGA-A7-A26J-01	0.118	0	0.02	0	0	0.294	0	0	0	0	0.013	0	0.037	0.05	0.025	0.379	0	0.015	0.044	0	0	0.003	0.430	0.030	1.079
TCGA-A7-A2KD-01	0.071	0	0	0	0	0.25	0	0.023	0.081	0	0.03	0.011	0.019	0.123	0.078	0.252	0	0.033	0.028	0	0	0	0.040	0.191	1.012
TCGA-A7-A3IY-01	0.005	0	0	0	0	0.074	0	0.015	0.037	0	0.023	0.004	0.01	0.528	0.015	0.237	0	0	0	0.053	0	0	0.000	0.668	0.744
TCGA-A7-A3IZ-01	0.116	0	0.065	0.014	0	0.083	0	0.006	0.016	0	0	0.02	0	0.087	0.013	0.278	0	0	0.302	0	0	0	0.310	0.055	1.074
TCGA-A7-A3J0-01	0.071	0	0.039	0.065	0	0.118	0	0.087	0.063	0	0	0.027	0.007	0.201	0.121	0.167	0	0	0.033	0	0	0	0.050	0.145	1.036
TCGA-A7-A3J1-01	0.113	0	0.01	0.038	0	0.389	0	0	0.039	0	0.087	0	0.037	0.011	0.067	0.158	0	0	0.051	0	0	0	0.040	0.185	1.017
TCGA-A7-A3RF-01	0.136	0	0.173	0	0	0.09	0	0.002	0.035	0	0	0.034	0.005	0.232	0	0.188	0	0.005	0.101	0	0	0	0.080	0.107	1.041
TCGA-A7-A425-01	0.056	0	0	0.048	0	0.227	0	0	0.004	0	0.05	0	0.084	0	0.021	0.433	0	0	0.078	0	0	0	0.070	0.115	1.047
TCGA-A7-A426-01	0.098	0	0.059	0.099	0	0.234	0	0.026	0	0	0.062	0.004	0.041	0	0.031	0.181	0	0	0.164	0	0	0	0.220	0.071	1.050
TCGA-A7-A4SA-01	0.089	0	0.004	0.123	0	0.413	0.004	0	0.001	0	0.048	0	0.024	0	0.107	0.137	0.003	0.015	0.032	0	0	0	0.020	0.245	1.000
TCGA-A7-A4SB-01	0.093	0	0.171	0.062	0	0.137	0	0.027	0.028	0	0	0.062	0.017	0	0.04	0.233	0	0	0.13	0	0	0	0.990	-0.044	1.080
TCGA-A7-A4SC-01	0.139	0	0.034	0.19	0	0.274	0	0.01	0	0	0.004	0.009	0.11	0.008	0.026	0.118	0	0.016	0.048	0	0.013	0	0.720	-0.000	1.090
TCGA-A7-A4SD-01	0.08	0	0.119	0.064	0	0.231	0.004	0.045	0.016	0	0.033	0.01	0.015	0.138	0.144	0.055	0	0.021	0.026	0	0	0	0.010	0.259	0.982
TCGA-A7-A4SE-01	0.026	0	0	0	0	0.193	0	0.057	0.006	0	0.003	0.025	0.004	0.478	0.104	0.077	0	0	0.029	0	0	0	0.010	0.312	0.989
TCGA-A7-A4SF-01	0.121	0	0.031	0	0	0.214	0	0	0.062	0	0.022	0.007	0.012	0.239	0.096	0.108	0	0	0.087	0	0	0	0.070	0.123	1.040

TCGA-A7-A56D-01	0.015	0	0.077	0	0	0	0	0.045	0.041	0.006	0	0.04	0.002	0.43	0	0.229	0	0.062	0.054	0	0	0	0.010	0.415	0.924
TCGA-A7-A5ZV-01	0.047	0	0	0.032	0	0.07	0	0.038	0.025	0	0	0.031	0.01	0.282	0.008	0.319	0.004	0.081	0.05	0	0	0.003	0.040	0.161	1.050
TCGA-A7-A5ZW-01	0.085	0	0.015	0.047	0	0.214	0	0.006	0.031	0	0.02	0	0.02	0.155	0.049	0.187	0	0	0.171	0	0	0	0.060	0.135	1.030
TCGA-A7-A5ZX-01	0.135	0	0.124	0.01	0	0.129	0	0.013	0.003	0	0	0.02	0	0.085	0.013	0.256	0	0	0.212	0	0	0	0.280	0.059	1.054
TCGA-A7-A6VV-01	0.005	0	0.001	0	0	0.172	0	0	0.066	0	0.026	0.006	0	0.507	0.04	0.123	0	0	0.053	0	0	0	0.010	0.377	0.953
TCGA-A7-A6VW-01	0.045	0	0.037	0.013	0	0.092	0	0.056	0.04	0	0	0.043	0.014	0.16	0	0.255	0	0.197	0	0	0	0.047	0.040	0.177	1.026
TCGA-A7-A6VX-01	0	0.008	0	0	0	0.375	0	0.037	0.066	0	0.045	0.004	0.041	0.294	0.021	0.021	0	0	0.089	0	0	0	0.210	0.077	1.075
TCGA-A7-A6VY-01	0.029	0	0.098	0	0	0.143	0.009	0.068	0.019	0	0.054	0	0	0.298	0.149	0.132	0	0	0.001	0	0	0	0.010	0.348	0.949
TCGA-A8-A06N-01	0.068	0	0.136	0	0	0.128	0	0.021	0.019	0	0	0.041	0.01	0.022	0.019	0.444	0	0.001	0.092	0	0	0	0.840	-0.016	1.091
TCGA-A8-A06O-01	0.076	0	0.054	0	0	0.216	0	0.017	0.028	0	0	0.013	0.014	0.155	0.093	0.247	0.028	0	0.06	0	0	0	0.060	0.127	1.036
TCGA-A8-A06P-01	0.007	0	0.046	0.03	0	0.133	0	0.006	0.049	0	0.03	0	0.011	0.163	0.011	0.367	0	0	0.148	0	0	0	0.090	0.101	1.059
TCGA-A8-A06Q-01	0.135	0	0.121	0.052	0	0.145	0	0	0	0	0	0.058	0.021	0.029	0.039	0.27	0.004	0	0.126	0	0	0	1.000	-0.049	1.087
TCGA-A8-A06R-01	0.06	0	0.174	0	0	0.176	0	0.017	0.043	0.003	0	0	0	0.21	0.087	0.185	0	0	0.044	0	0	0	0.010	0.422	0.906
TCGA-A8-A06T-01	0.033	0	0.16	0.003	0	0.282	0.009	0	0	0.018	0	0.029	0.025	0.045	0.109	0.189	0.001	0.019	0.068	0	0.008	0	0.090	0.100	1.037
TCGA-A8-A06U-01	0.127	0	0.029	0.022	0	0.209	0	0.055	0.046	0	0.002	0.032	0.018	0.043	0.096	0.256	0	0.005	0.06	0	0	0	0.070	0.119	1.036
TCGA-A8-A06Y-01	0.159	0	0.007	0	0	0.049	0	0.073	0.054	0	0	0.042	0	0.196	0.056	0.21	0	0.087	0.067	0	0	0	0.770	-0.010	1.097
TCGA-A8-A06Z-01	0.031	0	0	0	0	0.178	0	0	0.037	0	0	0.045	0	0.201	0.02	0.426	0	0	0.063	0	0	0	0.120	0.095	1.081
TCGA-A8-A075-01	0.059	0	0.116	0.036	0	0.217	0	0.032	0.017	0	0.033	0.011	0.011	0.127	0.078	0.202	0.013	0	0.049	0	0	0	0.020	0.225	0.989
TCGA-A8-A076-01	0.13	0	0.115	0	0	0.197	0	0	0.025	0	0.009	0.006	0	0.18	0.049	0.219	0	0	0.069	0	0	0.001	0.060	0.128	1.030
TCGA-A8-A079-01	0	0	0.123	0	0	0.283	0	0.01	0.048	0	0.065	0	0.05	0.222	0.006	0.151	0	0	0.031	0	0.011	0	0.340	0.046	1.068
TCGA-A8-A07B-01	0.055	0	0.072	0	0	0.279	0	0.002	0	0	0	0.045	0.014	0.069	0.066	0.264	0.002	0.013	0.119	0	0	0	0.580	0.018	1.071
TCGA-A8-A07C-01	0.011	0	0.145	0	0	0.133	0.001	0.029	0.003	0.07	0	0.07	0	0.065	0.103	0.266	0	0.018	0.072	0	0.014	0	0.040	0.176	1.013
TCGA-A8-A07E-01	0.084	0	0.008	0.036	0	0.332	0	0	0.019	0	0.027	0	0.02	0	0.088	0.202	0.028	0	0.154	0	0	0	0.120	0.095	1.046
TCGA-A8-A07F-01	0.078	0	0.02	0.038	0	0.333	0	0.018	0.007	0	0	0.031	0.016	0.103	0.065	0.197	0.006	0	0.088	0	0	0	0.890	-0.019	1.092
TCGA-A8-A07G-01	0.123	0	0.029	0.052	0	0.245	0	0.013	0	0	0.026	0	0.002	0.149	0.054	0.241	0	0.017	0.049	0	0	0	0.210	0.073	1.058
TCGA-A8-A07I-01	0.031	0	0.057	0.007	0	0.149	0	0.015	0.066	0	0.017	0	0	0.297	0.063	0.258	0	0	0.041	0	0	0	0.020	0.246	1.000
TCGA-A8-A07J-01	0.073	0	0	0	0	0.108	0	0.01	0.024	0	0.006	0.003	0.009	0.269	0.032	0.3	0.055	0	0.108	0	0	0.004	0.010	0.315	0.968
TCGA-A8-A07L-01	0.137	0	0.054	0.036	0	0.136	0	0.026	0.037	0	0	0.014	0	0.131	0.036	0.337	0	0.002	0.054	0	0	0	0.770	-0.010	1.093
TCGA-A8-A07O-01	0.045	0	0.002	0.017	0	0.176	0	0	0.039	0	0.028	0	0	0.589	0.019	0.058	0	0.011	0	0.018	0	0	0.010	0.394	0.954
TCGA-A8-A07P-01	0.048	0	0.085	0.022	0	0.182	0.006	0	0	0	0.001	0.008	0.057	0.026	0.035	0.272	0	0.019	0.239	0	0	0	0.030	0.199	1.001
TCGA-A8-A07R-01	0.192	0	0.171	0	0	0.148	0	0.011	0.024	0	0.011	0.01	0.007	0.146	0.067	0.121	0.007	0.056	0.011	0	0	0.018	0.310	0.053	1.055
TCGA-A8-A07S-01	0	0.006	0	0	0	0.31	0	0.035	0.048	0	0.125	0	0.042	0.191	0.015	0.176	0	0	0.044	0	0.008	0	0.450	0.025	1.090
TCGA-A8-A07U-01	0.094	0	0.107	0	0	0.184	0.001	0.054	0.037	0	0	0.036	0.013	0.112	0.181	0.074	0	0.012	0.095	0	0	0	0.000	0.449	0.893
TCGA-A8-A07W-01	0.059	0	0.002	0	0	0.176	0	0.019	0.028	0	0.006	0.009	0.005	0.329	0.056	0.261	0	0	0.05	0	0	0	0.040	0.175	1.044
TCGA-A8-A07Z-01	0.038	0	0.012	0.013	0	0.137	0	0.015	0.125	0	0.059	0.031	0.076	0.009	0	0.262	0	0.178	0.046	0	0	0	0.190	0.080	1.054
TCGA-A8-A081-01	0.101	0	0.057	0.083	0	0.206	0.009	0	0.045	0	0	0.021	0.024	0.178	0.097	0.078	0	0	0.1	0	0	0	0.040	0.165	1.017
TCGA-A8-A082-01	0.001	0	0.003	0	0	0.266	0.001	0.003	0.054	0	0.154	0	0.032	0.072	0	0.315	0.007	0	0.088	0	0	0.005	0.040	0.170	1.025
TCGA-A8-A083-01	0.091	0	0.138	0	0	0.123	0	0.087	0.004	0	0	0.074	0.047	0.061	0.039	0.298	0	0.004	0.034	0	0	0	0.760	-0.009	1.077
TCGA-A8-A084-01	0.097	0	0.017	0	0	0.082	0	0.035	0.034	0	0.017	0.01	0	0.418	0.076	0.154	0	0	0.041	0	0	0.021	0.340	0.046	1.118
TCGA-A8-A085-01	0.172	0	0.043	0	0	0.033	0	0	0.061	0	0.014	0	0	0.294	0	0.304	0	0	0.08	0	0	0	0.060	0.128	1.063
TCGA-A8-A086-01	0.017	0	0.002	0	0.012	0.297	0	0.005	0.067	0	0.068	0	0.009	0.329	0.002	0.125	0	0	0.068	0	0	0	0.300	0.057	1.091
TCGA-A8-A08A-01	0.096	0	0	0.017	0	0.142	0	0.063	0.022	0	0.006	0.013	0.002	0.211	0.065	0.34	0	0	0.023	0	0	0	0.070	0.113	1.062
TCGA-A8-A08B-01	0.071	0	0.105	0	0	0.28	0	0.08	0	0	0.061	0.015	0.012	0.105	0.134	0.071	0	0.003	0.062	0	0	0	0.180	0.085	1.053
TCGA-A8-A08G-01	0.069	0	0.058	0	0	0.237	0	0.03	0.046	0	0.011	0.008	0.006	0.126	0.097	0.26	0	0	0.051	0	0	0	0.060	0.130	1.034
TCGA-A8-A08H-01	0.035	0	0	0.022	0	0.14	0	0	0.001	0	0.016	0.007	0.111	0.16	0.015	0.394	0.008	0	0.071	0	0	0.02	0.000	0.508	0.861
TCGA-A8-A08I-01	0.006	0	0.02	0.042	0	0.164	0	0	0.056	0	0	0.023	0.01	0.478	0.098	0.06	0	0	0.044	0	0	0	0.010	0.411	0.926
TCGA-A8-A08J-01	0.066	0	0.12	0.007	0	0.08	0	0.012	0.024	0	0	0.009	0	0.28	0.055	0.307	0	0	0.036	0	0	0.004	0.010	0.384	0.930
TCGA-A8-A08L-01	0.072	0	0.019	0	0	0.263	0	0	0.036	0	0.008	0.044	0.049	0.139	0.091	0.169	0.002	0.002	0.106	0	0	0	0.030	0.199	1.004
TCGA-A8-A08O-01	0.142	0	0.095	0.012	0	0.295	0	0	0.006	0	0.023	0.013	0.018	0.035	0.041	0.266	0	0	0.054	0	0	0	0.980	-0.038	1.088
TCGA-A8-A08R-01	0.011	0	0.044	0	0	0.121	0.001	0	0.029	0.009	0.002	0.016	0	0.48	0.141	0.111	0	0	0.055	0	0	0	0.000	0.540	0.843
TCGA-A8-A08S-01	0.089	0	0.053	0	0	0.099	0	0.052	0.048	0	0	0.043	0	0.365	0.036	0.132	0	0.007	0.074	0	0	0	0.380	0.039	1.096
TCGA-A8-A08T-01	0.041	0	0.013	0.029	0	0.13	0	0.024	0.024	0	0.014	0.001	0	0.337	0.023	0.264	0	0	0.101	0	0	0	0.090	0.099	1.081
TCGA-A8-A08X-01	0.045	0	0.286	0	0	0.184	0	0	0.06	0	0.014	0.002	0.009	0.141	0.029	0.155	0	0.044	0.031	0	0	0	0.010	0.328	0.946
TCGA-A8-A08Z-01	0.041	0	0.001	0.011	0	0.21	0	0	0	0	0.02	0	0.028	0.231	0.035	0.356	0	0	0.066	0	0	0	0.010	0.251	1.000
TCGA-A8-A090-01	0.036	0	0.004	0	0	0.116	0	0.037	0.021	0	0	0.006	0	0.384	0.075	0.232	0	0	0.073	0	0	0	0.030	0.216	1.037
TCGA-A8-A091-01	0.112	0	0.06	0.024	0	0.248	0	0.011	0.025	0	0.01	0	0.01	0.127	0.042	0.272	0	0	0.058	0	0	0	0.950	-0.026	1.092
TCGA-A8-A092-01	0.003	0	0.004	0	0	0.326	0	0.047	0.029	0	0	0.031	0.049	0.106	0.018	0.186	0.002	0.009	0.183	0	0.005	0	0.320	0.049	1.068

TCGA-A8-A093-01	0.093	0	0.028	0	0	0.14	0	0.013	0.038	0	0.036	0	0.005	0.219	0.007	0.328	0	0	0.093	0	0	0	0.760	-0.009	1.108
TCGA-A8-A094-01	0.085	0	0.051	0.02	0	0.196	0.019	0.018	0.01	0	0.015	0.001	0	0.204	0.146	0.178	0	0.01	0.047	0	0	0	0.010	0.354	0.941
TCGA-A8-A095-01	0.078	0	0.009	0.063	0	0.258	0	0.01	0.013	0	0.023	0.002	0.011	0.066	0.034	0.291	0.003	0.039	0.1	0	0	0	0.640	0.010	1.079
TCGA-A8-A096-01	0.003	0	0	0.094	0	0.294	0	0.057	0.011	0	0	0.032	0.034	0.011	0.113	0.234	0.025	0	0.089	0	0.003	0	0.020	0.237	0.994
TCGA-A8-A097-01	0.09	0	0.003	0	0	0.157	0	0.024	0.034	0	0.002	0.005	0	0.327	0.09	0.224	0	0	0.043	0	0	0	0.020	0.241	1.009
TCGA-A8-A099-01	0.129	0	0.061	0	0	0.321	0	0.002	0.026	0	0.014	0.007	0.015	0.011	0.047	0.296	0.008	0	0.064	0	0	0	0.590	0.017	1.072
TCGA-A8-A09A-01	0.083	0	0.007	0.063	0	0.29	0	0	0.034	0	0.021	0.012	0.034	0.087	0.111	0.157	0.017	0	0.084	0	0	0	0.050	0.145	1.029
TCGA-A8-A09B-01	0.149	0	0.042	0.092	0	0.216	0	0.003	0.021	0	0.026	0	0.09	0	0.05	0.272	0	0	0.04	0	0	0	0.870	-0.018	1.086
TCGA-A8-A09D-01	0.079	0	0.015	0.008	0	0.177	0	0.005	0.036	0	0.03	0	0	0.308	0.053	0.254	0	0	0.035	0	0	0	0.010	0.299	0.975
TCGA-A8-A09E-01	0.015	0	0.005	0	0	0.34	0	0.044	0.09	0	0.027	0	0.059	0.166	0.034	0.042	0	0.013	0.159	0	0.004	0	0.810	-0.013	1.095
TCGA-A8-A09G-01	0.12	0	0.086	0	0	0.274	0	0.058	0.053	0	0	0.055	0.057	0	0.114	0.121	0	0	0.064	0	0	0	0.070	0.111	1.039
TCGA-A8-A09I-01	0.075	0	0.032	0.023	0	0.167	0.034	0.04	0.023	0	0.036	0.006	0.054	0.09	0.073	0.228	0.017	0.047	0.031	0	0	0.024	0.050	0.138	1.025
TCGA-A8-A09K-01	0.039	0	0.046	0.106	0	0.219	0.013	0.031	0.041	0	0.02	0.007	0.029	0.074	0.057	0.133	0	0	0.184	0	0	0	0.060	0.130	1.029
TCGA-A8-A09M-01	0.133	0	0.068	0.013	0	0.254	0	0.041	0.054	0	0.035	0.013	0.011	0.033	0.092	0.211	0.005	0	0.036	0	0	0	0.040	0.153	1.020
TCGA-A8-A09N-01	0.085	0	0.065	0.083	0	0.211	0	0.012	0.05	0	0	0.058	0.032	0.024	0.076	0.145	0.009	0	0.15	0	0	0	0.890	-0.021	1.085
TCGA-A8-A09Q-01	0.037	0	0.042	0.069	0	0.193	0	0.029	0.002	0	0	0.04	0.035	0.112	0.043	0.265	0	0	0.133	0	0	0	0.440	0.027	1.072
TCGA-A8-A09R-01	0.057	0	0.004	0.009	0	0.128	0	0.056	0.057	0	0.005	0.017	0	0.309	0.111	0.197	0	0	0.05	0	0	0	0.020	0.248	1.002
TCGA-A8-A09T-01	0.005	0	0	0	0	0.388	0	0.003	0	0	0	0	0.055	0.043	0.026	0.368	0	0	0.111	0	0	0	0.240	0.066	1.070
TCGA-A8-A09X-01	0.113	0	0.044	0.059	0	0.307	0	0	0.058	0	0.022	0.008	0.02	0.039	0.059	0.212	0	0.001	0.058	0	0	0	0.020	0.245	0.984
TCGA-A8-A09Z-01	0.021	0	0.014	0	0	0.312	0.005	0.031	0.093	0	0.067	0.015	0.021	0.065	0.097	0.141	0	0	0.103	0	0.016	0	0.070	0.124	1.039
TCGA-A8-A0A1-01	0.039	0	0	0.015	0	0.145	0.003	0.064	0.04	0	0.02	0	0.004	0.44	0.071	0.113	0	0	0.045	0	0	0	0.010	0.439	0.905
TCGA-A8-A0A2-01	0.121	0	0.111	0.03	0	0.281	0	0.023	0.015	0	0.028	0	0.064	0	0.055	0.227	0	0	0.044	0	0	0	0.340	0.045	1.055
TCGA-A8-A0A4-01	0.092	0	0.133	0	0	0.112	0	0	0.044	0	0	0.056	0.013	0.058	0.052	0.277	0	0	0.163	0	0	0	0.410	0.036	1.059
TCGA-A8-A0A6-01	0.1	0	0.067	0.018	0	0.386	0	0.022	0.013	0	0.015	0.004	0.007	0.047	0.092	0.162	0.007	0.009	0.05	0	0	0	0.020	0.245	0.985
TCGA-A8-A0A7-01	0.08	0	0.08	0.068	0	0.295	0.01	0	0.055	0	0.018	0.008	0.014	0.116	0.097	0.108	0.002	0	0.051	0	0	0	0.010	0.434	0.900
TCGA-A8-A0A9-01	0.083	0	0.019	0.05	0	0.268	0	0.01	0.054	0	0.023	0	0.009	0.204	0.073	0.185	0	0	0.021	0	0	0	0.220	0.068	1.069
TCGA-A8-A0AB-01	0.053	0	0.067	0.007	0	0.352	0	0	0	0	0.012	0	0.033	0.223	0.002	0	0	0	0.244	0	0	0.007	0.570	0.018	1.087
TCGA-A8-A0AD-01	0.061	0	0.014	0.011	0	0.181	0	0	0	0	0.014	0	0.173	0	0.012	0.432	0	0.005	0.097	0	0	0	0.320	0.050	1.079
TCGA-AC-A23C-01	0.119	0	0.1	0.017	0	0.31	0	0.016	0.024	0	0.025	0	0.013	0.014	0.073	0.233	0.002	0	0.054	0	0	0	0.510	0.021	1.068
TCGA-AC-A23E-01	0.068	0	0.034	0	0	0.102	0	0.009	0.001	0	0	0.025	0	0.169	0.011	0.492	0	0	0.081	0	0	0.007	0.010	0.379	0.936
TCGA-AC-A23G-01	0.049	0	0	0.044	0	0.095	0	0.005	0.038	0	0.004	0.002	0	0.504	0.018	0.199	0	0	0.044	0	0	0	0.030	0.216	1.058
TCGA-AC-A23H-01	0.087	0	0.04	0	0	0.052	0	0.028	0.033	0.006	0	0.015	0	0.449	0.055	0.202	0	0	0.032	0	0	0	0.040	0.164	1.071
TCGA-AC-A23H-11	0.135	0	0.041	0.057	0	0.211	0	0.001	0	0	0	0.032	0.064	0	0	0.198	0	0.027	0.234	0	0	0	0.280	0.061	1.056
TCGA-AC-A2B8-01	0.058	0	0.024	0.066	0	0.342	0	0	0	0	0.028	0	0.062	0.067	0.062	0.228	0	0.002	0.06	0	0	0	0.070	0.113	1.041
TCGA-AC-A2BK-01	0.105	0	0.158	0	0	0.248	0	0.078	0.003	0	0	0.053	0.03	0.146	0.072	0	0	0	0.106	0	0	0	0.530	0.020	1.064
TCGA-AC-A2BM-01	0.044	0	0.069	0.029	0	0.391	0	0.009	0	0	0.033	0	0.003	0.05	0.039	0.234	0	0.025	0.074	0	0	0.001	0.070	0.115	1.038
TCGA-AC-A2FB-01	0.091	0	0.029	0.1	0	0.399	0.047	0	0	0	0.005	0	0.021	0.003	0.1	0.086	0	0.03	0.089	0	0	0	0.010	0.363	0.936
TCGA-AC-A2FB-11	0.07	0	0.097	0.042	0	0.247	0	0	0	0	0.021	0.015	0.05	0	0.024	0.325	0	0	0.108	0	0	0	0.060	0.131	1.025
TCGA-AC-A2FE-01	0.062	0	0	0.201	0	0.278	0	0.023	0.041	0	0.019	0.03	0.075	0.041	0.115	0.062	0.006	0	0.045	0	0	0	0.010	0.264	0.990
TCGA-AC-A2FF-01	0.144	0	0.087	0.066	0	0.34	0	0	0	0	0.021	0	0.016	0	0.035	0.165	0.032	0.022	0.072	0	0	0	0.040	0.191	1.003
TCGA-AC-A2FG-01	0.112	0	0.129	0	0	0.238	0	0	0	0	0.03	0	0.01	0.071	0.019	0.207	0.048	0.006	0.13	0	0	0	0.600	0.016	1.059
TCGA-AC-A2FK-01	0.129	0	0.077	0.064	0	0.255	0	0	0	0	0.015	0.024	0.038	0.17	0.031	0.126	0	0	0.07	0	0	0	0.070	0.110	1.036
TCGA-AC-A2FM-01	0.134	0	0.034	0.033	0	0.314	0	0	0.044	0	0.027	0.002	0.023	0.004	0.055	0.241	0	0	0.083	0.007	0	0	1.000	-0.050	1.099
TCGA-AC-A2FM-11	0.06	0	0.11	0.017	0	0.193	0	0.008	0	0	0	0.057	0.059	0	0.001	0.367	0	0.005	0.099	0.01	0	0.016	0.280	0.060	1.055
TCGA-AC-A2FO-01	0.125	0	0.035	0.072	0	0.42	0.011	0	0	0	0.021	0	0.032	0	0.058	0.169	0.034	0	0.023	0	0	0	0.020	0.248	0.988
TCGA-AC-A2QH-01	0	0.004	0	0	0	0.46	0	0.04	0.033	0	0.096	0.01	0	0.289	0	0	0	0	0	0.067	0	0	0.610	0.013	1.127
TCGA-AC-A2QI-01	0.092	0	0.009	0.061	0	0.233	0	0.038	0.034	0	0.031	0	0.004	0.127	0.083	0.24	0	0	0.05	0	0	0	0.040	0.155	1.026
TCGA-AC-A2QJ-01	0	0.005	0	0.022	0	0.002	0	0.038	0.025	0	0	0.009	0.032	0.298	0.01	0.518	0	0	0.041	0	0	0	0.000	0.646	0.763
TCGA-AC-A3BB-01	0.067	0.003	0	0.106	0	0.299	0	0	0.053	0	0.004	0	0.067	0.071	0.133	0.145	0	0	0.052	0	0	0	0.010	0.257	0.986
TCGA-AC-A3EH-01	0.072	0	0.02	0.046	0	0.146	0	0.01	0.031	0	0.013	0	0	0.375	0.049	0.199	0	0	0.039	0	0	0	0.040	0.158	1.054
TCGA-AC-A3HN-01	0.086	0	0.034	0.006	0	0.113	0	0.04	0.04	0	0	0.027	0.001	0.3	0.046	0.171	0	0	0.136	0	0	0	0.010	0.304	0.966
TCGA-AC-A3OD-01	0.047	0	0.036	0.017	0	0.547	0.013	0	0	0	0.025	0	0.023	0.002	0.085	0.116	0.026	0.016	0.046	0	0	0	0.010	0.268	0.985
TCGA-AC-A3QP-01	0.078	0	0.038	0.084	0	0.096	0	0.025	0.018	0	0.004	0.006	0.123	0	0.016	0.428	0	0.002	0.082	0	0	0	0.050	0.141	1.033
TCGA-AC-A3QQ-01	0.033	0	0.103	0.022	0	0.33	0.013	0	0	0	0.079	0	0.059	0.036	0.146	0.048	0.017	0	0.112	0	0	0	0.600	0.015	1.083
TCGA-AC-A3TM-01	0.095	0	0.074	0.165	0	0.183	0	0.014	0.067	0	0	0.019	0.006	0.041	0.029	0.272	0	0	0.035	0	0	0	0.070	0.108	1.041
TCGA-AC-A3TN-01	0.073	0	0.072	0.008	0	0.247	0	0.014	0.063	0	0.04	0	0.017	0.136	0.014	0.219	0	0.011	0.086	0	0	0	0.390	0.038	1.062
TCGA-AC-A3W5-01	[illegible]	[illegible]	[illegible]	[illegible]	[illegible]	[illegible]	[illegible]	[illegible]	[illegible]	[illegible]	[illegible]	[illegible]	[illegible]	[illegible]	[illegible]	[illegible]	[illegible]	[illegible]	[illegible]	[illegible]	[illegible]	[illegible]			

	0.08	0	0.02	0.025	0	0.196	0	0.036	0.075	0	0.019	0.009	0.002	0.267	0.08	0.14	0	0	0.051	0	0	0	0.010	0.296	0.968
TCGA-AC-A3W6-01	0.018	0	0	0.081	0	0.364	0	0.008	0.042	0	0	0	0.028	0.043	0.149	0.125	0.015	0	0.125	0	0.002	0	0.030	0.217	1.009
TCGA-AC-A3W7-01	0.092	0	0.03	0.072	0	0.283	0	0	0.034	0	0.051	0	0.018	0.006	0.047	0.271	0.006	0.007	0.083	0	0	0	0.740	-0.004	1.083
TCGA-AC-A3YI-01	0.052	0	0.055	0.008	0	0.1	0	0.008	0.029	0	0.017	0.004	0.026	0.374	0.029	0.174	0	0.029	0.089	0.006	0	0	0.030	0.207	1.025
TCGA-AC-A3YJ-01	0.043	0	0	0.034	0	0.085	0	0.008	0.016	0	0.017	0	0.055	0	0.022	0.665	0.005	0	0.05	0	0	0	0.040	0.180	1.053
TCGA-AC-A4ZE-01	0.111	0	0.043	0.016	0	0.401	0	0	0	0	0	0.013	0.063	0	0.036	0.211	0	0	0.105	0	0	0	0.480	0.022	1.072
TCGA-AC-A5EH-01	0.001	0.003	0	0	0	0.118	0	0.035	0.044	0	0.027	0.009	0	0.386	0.03	0.291	0	0	0.058	0	0	0	0.000	0.544	0.839
TCGA-AC-A5XS-01	0.076	0	0.059	0.066	0	0.335	0	0.01	0.005	0	0	0.015	0.014	0	0.08	0.288	0	0.001	0.029	0	0	0.022	0.030	0.205	1.003
TCGA-AC-A5XU-01	0.01	0	0.192	0	0	0.114	0	0.017	0.046	0	0	0.049	0	0.257	0.051	0.204	0	0	0.06	0	0	0	0.010	0.261	0.981
TCGA-AC-A62V-01	0.002	0.001	0.023	0	0	0.121	0	0	0.029	0	0.067	0	0.004	0.375	0	0.252	0.009	0	0.116	0	0	0	0.010	0.358	0.953
TCGA-AC-A62X-01	0	0.007	0.007	0.024	0	0.085	0	0.085	0.01	0	0	0.065	0.09	0.236	0.01	0	0	0.22	0.149	0	0	0.014	0.090	0.100	1.060
TCGA-AC-A62Y-01	0.043	0	0	0.026	0	0.132	0	0.013	0.007	0	0.016	0	0	0.463	0.036	0.247	0	0	0.017	0	0	0	0.030	0.216	1.055
TCGA-AC-A6IV-01	0.02	0	0.008	0.105	0	0.204	0	0	0.016	0	0	0	0.013	0.18	0.035	0.261	0	0.017	0.132	0	0	0.008	0.040	0.188	1.016
TCGA-AC-A6IW-01	0.006	0.01	0	0	0	0.208	0	0.076	0.025	0	0	0.059	0.012	0.031	0.172	0.229	0.11	0	0.061	0	0.001	0	0.010	0.349	0.944
TCGA-AC-A6IX-01	0.098	0	0.181	0.034	0	0.233	0	0.01	0.012	0	0.024	0	0.037	0	0.043	0.253	0	0.034	0.042	0	0	0	0.060	0.134	1.018
TCGA-AC-A6IX-06	0.18	0.063	0.017	0.103	0	0.355	0	0	0.009	0	0.042	0	0.04	0	0.025	0.127	0	0.023	0.014	0	0	0	0.010	0.290	0.972
TCGA-AC-A6NO-01	0.08	0	0.045	0.037	0	0.229	0	0.051	0.026	0	0	0.025	0.015	0.151	0.055	0.204	0.02	0.022	0.042	0	0	0	0.720	-0.002	1.084
TCGA-AC-A7VB-01	0.199	0	0.183	0.028	0	0.053	0	0.012	0.072	0	0	0.015	0.03	0.237	0.017	0.116	0	0	0.039	0	0	0	0.980	-0.041	1.095
TCGA-AC-A7VC-01	0.012	0	0.007	0	0	0.202	0	0.061	0.016	0	0.069	0.013	0.011	0.568	0	0.029	0.012	0	0	0	0	0	0.000	0.725	0.696
TCGA-AC-A8OP-01	0.146	0	0	0.015	0	0.263	0	0.015	0.007	0	0.061	0	0.033	0.134	0.048	0.195	0	0	0.083	0	0	0	0.410	0.037	1.068
TCGA-AC-A8OQ-01	0.043	0	0.208	0	0	0.219	0	0.023	0.086	0	0.051	0.021	0.036	0.151	0.07	0.069	0	0	0.023	0	0	0	0.010	0.285	0.963
TCGA-AC-A8OR-01	0.001	0	0.003	0	0	0.218	0	0.01	0.095	0	0.091	0	0.019	0.29	0.002	0.224	0	0	0.048	0	0	0	0.060	0.133	1.055
TCGA-AC-A8OS-01	0.069	0	0.015	0.125	0	0.274	0	0	0	0	0.058	0	0.053	0.02	0.063	0.141	0.002	0	0.18	0	0	0	0.070	0.117	1.040
TCGA-AN-A03X-01	0.051	0	0.045	0.045	0	0.149	0	0.008	0	0	0	0.02	0	0	0.039	0.451	0.011	0.017	0.137	0	0	0.028	0.340	0.048	1.073
TCGA-AN-A03Y-01	0.074	0	0	0	0.027	0.163	0.009	0	0.076	0	0.055	0	0.014	0.088	0.174	0.118	0.014	0	0.189	0	0	0	0.280	0.059	1.075
TCGA-AN-A041-01	0.043	0	0.05	0	0	0.158	0	0.025	0.031	0	0	0.012	0	0.342	0.051	0.227	0	0	0.06	0	0	0	0.060	0.128	1.063
TCGA-AN-A046-01	0.058	0	0	0.029	0	0.296	0.007	0.076	0.021	0	0.041	0	0.036	0.068	0.119	0.157	0.016	0.019	0.044	0	0.013	0	0.010	0.263	0.980
TCGA-AN-A049-01	0.075	0	0.047	0.039	0	0.315	0.003	0	0	0	0.039	0	0.012	0.163	0.06	0.202	0.016	0	0.027	0	0	0	0.070	0.116	1.042
TCGA-AN-A04A-01	0.084	0	0.1	0	0	0.317	0	0	0	0	0.029	0	0.009	0.194	0.04	0.192	0	0	0.034	0	0	0	0.220	0.070	1.057
TCGA-AN-A04C-01	0.058	0	0.098	0.061	0	0.303	0	0.017	0.018	0	0.03	0	0.016	0.04	0.067	0.242	0.001	0.047	0.002	0	0	0	0.210	0.076	1.054
TCGA-AN-A04D-01	0.005	0	0.036	0.003	0	0.269	0	0.149	0	0	0.01	0.03	0.08	0.151	0	0.03	0	0.01	0.22	0	0.006	0.003	0.940	-0.025	1.099
TCGA-AN-A0AJ-01	0.012	0	0	0	0	0.21	0	0.003	0.022	0.016	0	0	0	0.342	0.098	0.223	0.009	0.005	0.059	0	0	0	0.010	0.376	0.940
TCGA-AN-A0AK-01	0.08	0	0.027	0.002	0	0.212	0	0.029	0.052	0	0.012	0.004	0.009	0.245	0.066	0.243	0	0	0.019	0	0	0	0.060	0.128	1.049
TCGA-AN-A0AL-01	0.158	0	0.093	0	0	0.267	0	0.002	0.003	0	0.068	0	0.001	0.166	0.043	0.135	0	0.043	0.021	0	0	0	0.030	0.213	0.996
TCGA-AN-A0AM-01	0.108	0	0.078	0.009	0	0.244	0	0.049	0.056	0	0.029	0.022	0.015	0.068	0.143	0.175	0	0	0.005	0	0	0	0.040	0.154	1.028
TCGA-AN-A0AR-01	0.059	0	0	0.012	0	0.213	0	0.004	0.082	0	0.001	0.031	0.03	0.135	0.038	0.315	0.075	0	0.006	0	0	0	0.890	-0.022	1.109
TCGA-AN-A0AS-01	0.001	0.002	0.002	0	0	0.174	0	0.009	0.069	0	0.036	0.015	0	0.55	0	0.054	0	0	0.089	0	0	0	0.010	0.334	0.983
TCGA-AN-A0AT-01	0.099	0	0.026	0.022	0	0.226	0	0.022	0.026	0	0.028	0.003	0	0.353	0.04	0.147	0	0.005	0	0.002	0	0	0.010	0.340	0.955
TCGA-AN-A0FD-01	0.003	0	0.016	0	0	0.144	0	0.003	0.036	0	0	0.055	0	0.419	0.134	0.102	0.013	0	0.076	0	0	0	0.010	0.360	0.955
TCGA-AN-A0FF-01	0.05	0	0.026	0	0.033	0.12	0	0.037	0.032	0.007	0.028	0	0.016	0.411	0.02	0.162	0	0.007	0.048	0	0	0	0.000	0.530	0.848
TCGA-AN-A0FJ-01	0.091	0	0	0.045	0	0.239	0	0.045	0.036	0	0.046	0	0.016	0.249	0.095	0.113	0.015	0	0.01	0	0	0	0.090	0.100	1.060
TCGA-AN-A0FK-01	0.112	0	0.134	0	0	0.042	0	0.031	0.055	0	0	0.018	0	0.326	0.029	0.199	0	0	0.053	0	0	0	0.110	0.098	1.065
TCGA-AN-A0FL-01	0.082	0	0.012	0	0	0.16	0	0.008	0	0	0	0.005	0	0.432	0.01	0.189	0	0.064	0.038	0	0	0	0.040	0.189	1.053
TCGA-AN-A0FN-01	0.108	0	0	0.034	0	0.439	0	0	0.005	0	0	0.004	0.028	0.079	0.122	0.101	0.011	0	0.065	0	0.005	0	0.030	0.216	1.006
TCGA-AN-A0FS-01	0.159	0	0.039	0.004	0	0.205	0	0.036	0	0	0	0.013	0.006	0	0.009	0.336	0	0.005	0.184	0.004	0	0	0.680	0.005	1.080
TCGA-AN-A0FT-01	0.016	0	0.011	0	0	0.307	0	0.045	0.019	0	0.023	0.034	0.031	0.06	0.068	0.324	0.005	0	0.057	0	0	0	0.290	0.059	1.068
TCGA-AN-A0FV-01	0.043	0	0.031	0	0	0.037	0	0.057	0.025	0	0	0.02	0	0.416	0.012	0.262	0	0	0.097	0	0	0	0.010	0.303	0.991
TCGA-AN-A0FW-01	0.116	0	0.079	0.079	0	0.235	0	0	0.026	0	0	0.014	0.026	0.003	0.099	0.234	0.011	0	0.077	0	0	0	0.760	-0.009	1.081
TCGA-AN-A0FX-01	0.127	0	0.046	0.04	0	0.161	0	0.018	0.04	0	0.028	0	0	0.3	0.093	0.126	0	0	0.02	0	0	0	0.040	0.172	1.029
TCGA-AN-A0FY-01	0.048	0	0	0	0	0.294	0	0.042	0.044	0	0.021	0	0.042	0.307	0.014	0.139	0	0	0.049	0	0	0	0.310	0.053	1.087
TCGA-AN-A0FZ-01	0.129	0	0	0	0.003	0.057	0	0.04	0.03	0.027	0.008	0	0	0.358	0.029	0.289	0	0	0.029	0	0	0	0.040	0.157	1.063
TCGA-AN-A0G0-01	0	0	0.055	0	0	0.275	0	0.048	0.001	0	0.036	0	0.032	0.335	0.077	0.034	0.016	0	0.091	0	0	0	0.530	0.020	1.096
TCGA-AN-A0XL-01	0	0	0.032	0.023	0	0.367	0	0.013	0	0	0	0.024	0.069	0.1	0.011	0.194	0	0	0.167	0	0	0	0.320	0.048	1.068
TCGA-AN-A0XN-01	0.005	0	0	0	0	0.133	0	0	0.029	0	0.004	0.018	0	0.533	0.004	0.196	0	0.019	0.058	0	0	0	0.000	0.444	0.920
TCGA-AN-A0XO-01	0.035	0	0.003	0	0	0.2	0	0.01	0.061	0	0.044	0.016	0	0.479	0	0.011	0	0.09	0.051	0	0	0	0.040	0.153	1.068
TCGA-AN-A0XP-01	0.093	0	0.056	0	0	0.198	0	0.007	0.008	0	0.03	0.005	0	0.048	0.044	0.405	0	0.028	0.054	0	0	0.023	0.720	-0.001	1.089
TCGA-AN-A0XR-01	0.001	0.006	0.001	0	0	0.271	0	0.034	0.126	0	0.077	0	0	0.382	0	0	0	0.032	0.069	0	0	0	0.410	0.032	1.109

TCGA-AN-A0XS-01	0.018	0	0.133	0.124	0	0.213	0	0	0.026	0	0	0.015	0.041	0.068	0.136	0.102	0.014	0	0.11	0	0	0	0.070	0.116	1.037
TCGA-AN-A0XT-01	0.114	0	0.067	0.029	0	0.228	0	0	0.004	0	0.02	0	0.018	0.054	0.082	0.215	0	0	0.17	0	0	0	0.780	-0.011	1.079
TCGA-AN-A0XU-01	0.117	0	0.069	0.062	0	0.102	0.009	0.065	0.025	0	0.075	0.011	0	0.185	0.128	0.133	0.003	0	0.016	0	0	0	0.020	0.234	0.994
TCGA-AN-A0XV-01	0.178	0	0.042	0	0	0.312	0	0.028	0.006	0	0.023	0	0.033	0.101	0.089	0.114	0	0	0.074	0	0	0	0.310	0.052	1.061
TCGA-AN-A0XW-01	0.045	0	0	0.011	0	0.3	0.033	0.086	0.002	0	0.007	0.021	0.016	0.02	0.142	0.195	0.005	0	0.111	0	0.004	0	0.010	0.326	0.952
TCGA-AO-A03L-01	0.085	0	0.098	0.061	0	0.327	0	0.007	0.011	0	0.045	0	0.069	0	0.07	0.175	0	0.016	0.036	0	0	0	0.190	0.079	1.048
TCGA-AO-A03M-01	0.127	0	0.046	0	0	0.236	0	0.08	0.011	0	0.008	0.03	0.021	0.093	0.117	0.139	0	0	0.093	0	0	0	0.030	0.203	1.001
TCGA-AO-A03N-01	0.04	0	0.018	0	0	0.175	0	0.032	0.042	0	0.038	0	0	0.489	0	0.067	0	0.014	0.086	0	0	0	0.010	0.418	0.920
TCGA-AO-A03O-01	0.016	0	0.045	0	0	0.32	0.006	0.015	0.042	0	0.017	0.006	0.017	0.143	0.13	0.178	0	0	0.065	0	0	0	0.040	0.157	1.029
TCGA-AO-A03R-01	0.038	0.005	0	0.104	0.056	0.301	0	0	0.001	0	0	0.02	0.079	0.073	0.156	0.073	0.014	0	0.074	0	0.007	0	0.010	0.256	0.991
TCGA-AO-A03T-01	0.118	0	0.104	0.03	0	0.223	0	0.012	0.04	0	0.01	0.027	0.025	0.191	0.102	0.103	0	0	0.016	0	0	0	0.020	0.236	0.989
TCGA-AO-A03U-01	0	0	0.008	0.046	0	0.21	0	0	0	0	0.079	0.054	0.097	0	0.004	0.384	0.022	0.015	0.079	0	0	0	0.060	0.133	1.042
TCGA-AO-A03V-01	0.146	0	0.052	0.071	0	0.258	0	0.013	0.028	0	0.027	0	0.008	0.072	0.094	0.182	0	0.019	0.031	0	0	0	0.660	0.007	1.082
TCGA-AO-A0J2-01	0.034	0	0.005	0	0	0.071	0	0.137	0.051	0	0.003	0.106	0.035	0.196	0.152	0.09	0.001	0.005	0.091	0	0.024	0	0.040	0.176	1.029
TCGA-AO-A0J3-01	0.077	0	0.001	0.035	0	0.212	0	0.01	0.053	0	0	0.057	0.012	0.084	0.054	0.29	0	0	0.115	0	0	0	0.770	-0.010	1.091
TCGA-AO-A0J4-01	0.135	0	0	0	0	0.224	0	0.062	0.033	0	0.055	0.009	0.02	0.215	0.198	0.025	0.005	0	0.018	0	0	0	0.010	0.313	0.970
TCGA-AO-A0J5-01	0.057	0	0.101	0.084	0	0.259	0	0.003	0	0	0	0.038	0.042	0.12	0	0.137	0	0.01	0.15	0	0	0	0.500	0.021	1.067
TCGA-AO-A0J6-01	0.027	0	0.003	0.077	0	0.208	0	0.088	0.032	0	0.056	0.058	0.014	0.072	0.107	0.08	0	0.164	0.015	0	0	0	0.010	0.260	0.997
TCGA-AO-A0J7-01	0.025	0	0.02	0	0	0.274	0	0.005	0.057	0	0.144	0	0.037	0.259	0	0	0.039	0.009	0.131	0	0	0	0.810	-0.013	1.103
TCGA-AO-A0J8-01	0.097	0	0	0.026	0	0.151	0	0.033	0.053	0	0	0.018	0.003	0.412	0.035	0.098	0	0	0.076	0	0	0	0.020	0.226	1.017
TCGA-AO-A0J9-01	0.035	0	0	0.075	0	0.213	0	0.022	0.064	0	0.002	0.021	0.018	0.135	0.058	0.285	0.009	0	0.064	0	0	0	0.010	0.266	0.980
TCGA-AO-A0JA-01	0.088	0	0.067	0	0	0.389	0	0	0	0	0.013	0.005	0.009	0	0.074	0.281	0.003	0	0.072	0	0	0	0.150	0.088	1.048
TCGA-AO-A0JB-01	0.124	0	0.108	0.049	0	0.135	0	0.076	0.032	0	0	0.021	0.032	0.064	0.051	0.267	0	0.022	0.019	0	0	0	0.070	0.125	1.027
TCGA-AO-A0JC-01	0.287	0.027	0.002	0.072	0	0.335	0.048	0	0.014	0	0.058	0	0.019	0.019	0.043	0.066	0	0	0	0.009	0	0	0.000	0.554	0.835
TCGA-AO-A0JD-01	0.126	0	0	0.007	0	0.236	0	0.074	0.023	0	0.001	0.025	0.015	0.119	0.092	0.166	0.041	0.009	0.067	0	0	0	0.310	0.057	1.061
TCGA-AO-A0JE-01	0.009	0	0.04	0.031	0	0.304	0	0.032	0.086	0	0	0.033	0.017	0.043	0.135	0.127	0	0.001	0.143	0	0	0	0.020	0.224	0.999
TCGA-AO-A0JF-01	0.07	0	0	0.049	0	0.331	0	0	0	0	0.035	0	0.029	0.014	0.034	0.25	0.063	0	0.125	0	0	0	0.090	0.099	1.043
TCGA-AO-A0JG-01	0.131	0	0.056	0.085	0	0.381	0	0.027	0	0	0.021	0	0.025	0.049	0.073	0.125	0	0	0.027	0	0	0	0.090	0.103	1.051
TCGA-AO-A0JI-01	0.099	0	0.101	0.026	0	0.155	0	0	0	0	0	0.023	0	0.003	0.034	0.044	0	0	0.514	0	0	0	0.010	0.254	1.017
TCGA-AO-A0JJ-01	0.02	0	0.114	0.035	0.016	0.25	0	0	0.01	0	0	0.025	0.045	0.047	0.111	0.158	0.014	0	0.153	0	0.002	0	0.090	0.100	1.038
TCGA-AO-A0JL-01	0.178	0	0.04	0	0	0.097	0	0.036	0.057	0	0	0.013	0.002	0.377	0.046	0.113	0	0	0.042	0	0	0	0.040	0.183	1.032
TCGA-AO-A0JM-01	0.084	0	0.08	0	0	0.066	0	0.046	0.059	0	0	0	0	0.407	0.029	0.177	0	0.005	0.046	0	0	0	0.010	0.343	0.957
TCGA-AO-A124-01	0.12	0	0	0.068	0	0.202	0	0.043	0.019	0	0.035	0	0.024	0.222	0.011	0.084	0	0.136	0	0.036	0	0	0.130	0.090	1.059
TCGA-AO-A125-01	0.058	0	0.051	0	0	0.078	0	0.179	0	0	0	0.038	0.026	0.233	0	0.193	0	0.132	0.011	0	0	0	0.760	-0.009	1.101
TCGA-AO-A126-01	0.119	0	0.078	0.001	0	0.252	0	0.014	0.064	0	0	0.04	0.015	0	0.043	0.245	0	0	0.13	0	0	0	0.220	0.071	1.046
TCGA-AO-A128-01	0.02	0	0	0.111	0	0.213	0.013	0.042	0.047	0	0	0.048	0.007	0.075	0.256	0.084	0.007	0	0.077	0	0	0.001	0.000	0.528	0.850
TCGA-AO-A129-01	0.102	0	0.046	0.016	0	0.195	0	0.051	0.038	0	0.006	0.032	0.04	0.221	0.163	0.021	0	0.001	0.067	0	0	0	0.010	0.339	0.948
TCGA-AO-A12A-01	0.117	0	0.018	0.081	0	0.312	0	0.002	0.034	0	0.026	0.001	0.017	0	0.057	0.221	0.025	0.058	0.033	0	0	0	0.310	0.051	1.067
TCGA-AO-A12B-01	0.07	0	0.055	0	0	0.226	0	0.013	0.032	0	0.039	0	0.044	0.01	0.086	0.372	0	0	0.055	0	0	0	0.640	0.009	1.081
TCGA-AO-A12C-01	0.042	0	0.037	0	0	0.181	0	0.022	0.047	0	0	0.028	0	0.42	0.005	0.136	0	0	0.083	0	0	0	0.040	0.173	1.049
TCGA-AO-A12D-01	0.003	0	0.036	0	0	0.368	0.008	0.022	0.009	0	0.024	0.005	0.019	0.141	0.128	0.126	0	0	0.111	0	0	0	0.010	0.288	0.970
TCGA-AO-A12E-01	0.073	0	0.063	0	0	0.222	0	0	0	0	0.017	0.014	0.026	0.018	0.011	0.362	0.001	0.009	0.183	0	0	0	0.130	0.091	1.047
TCGA-AO-A12F-01	0.043	0	0.042	0.035	0	0.279	0	0.033	0	0	0.005	0.033	0.069	0.098	0	0.234	0	0.021	0.103	0	0	0.006	0.640	0.010	1.076
TCGA-AO-A12G-01	0.119	0	0.042	0.045	0	0.262	0	0	0.035	0	0.028	0	0.027	0	0.063	0.305	0	0	0.074	0	0	0	0.320	0.049	1.061
TCGA-AO-A12H-01	0.001	0	0.126	0	0	0.335	0	0.063	0.011	0	0.047	0	0.023	0.162	0	0.19	0	0	0.039	0	0.003	0	0.720	-0.002	1.086
TCGA-AO-A1KO-01	0.097	0	0.109	0.062	0	0.382	0	0	0	0	0.017	0.022	0.032	0.012	0	0.162	0	0.067	0.04	0	0	0	0.200	0.079	1.051
TCGA-AO-A1KP-01	0.196	0	0.023	0	0	0.322	0	0.082	0.001	0	0	0.039	0.002	0.109	0.025	0.051	0.004	0.075	0.071	0	0	0	0.980	-0.038	1.102
TCGA-AO-A1KQ-01	0.08	0	0.013	0.146	0	0.187	0.007	0.049	0.036	0	0.027	0	0	0.237	0.052	0.144	0	0	0.023	0	0	0	0.010	0.328	0.954
TCGA-AO-A1KR-01	0.091	0	0.07	0.152	0	0.155	0	0.13	0.028	0	0.046	0.041	0.027	0.021	0.071	0.063	0	0.088	0.019	0	0	0	0.430	0.027	1.088
TCGA-AO-A1KS-01	0.097	0	0.06	0	0	0.372	0	0	0	0	0	0.044	0.015	0.038	0.026	0.177	0.004	0.054	0.112	0	0	0	0.740	-0.004	1.078
TCGA-AO-A1KT-01	0.02	0	0.007	0	0	0.207	0	0.018	0.006	0	0.013	0	0	0.189	0.014	0.398	0	0.067	0.053	0	0	0.007	0.040	0.148	1.050
TCGA-AQ-A04H-01	0.039	0	0.002	0	0	0.319	0	0.037	0.034	0	0.034	0	0.055	0.286	0.044	0.103	0	0	0.045	0	0	0	0.570	0.018	1.097
TCGA-AQ-A04J-01	0.186	0	0.012	0.03	0	0.421	0.01	0	0	0	0.071	0	0.035	0.09	0.046	0.067	0	0.032	0	0	0	0	0.010	0.308	0.963
TCGA-AQ-A04L-01	0.004	0	0	0	0	0.237	0	0.089	0.018	0	0.047	0	0.014	0.405	0	0.128	0	0.003	0.057	0	0	0	0.210	0.074	1.100
TCGA-AQ-A0Y5-01	0.054	0	0.09	0	0	0.067	0	0.013	0.007	0	0	0.015	0	0.287	0.033	0.371	0	0	0.055	0	0	0.007	0.010	0.426	0.911
TCGA-AQ-A1H2-01	0.065	0	0.037	0.012	0	0.169	0	0	0.007	0	0	0.003	0.19	0	0.043	0.407	0	0	0.066	0	0	0	0.530	0.020	1.089
TCGA-AQ-A1H3-01	0.027	0	0.028	0.131	0	0.193	0	0.033	0.013	0	0.04	0	0.069	0.172	0.074	0.073	0.004	0	0.132	0	0.011	0	0.290	0.059	1.061

TCGA-AQ-A54N-01	0.02	0	0.045	0.13	0	0.119	0	0.134	0.009	0	0	0.045	0.044	0.03	0.081	0.304	0	0.01	0.029	0	0	0	0.960	-0.036	1.104
TCGA-AQ-A54O-01	0.042	0	0.003	0.018	0	0.039	0	0.124	0.06	0	0.013	0	0	0.388	0.061	0.211	0	0	0.041	0	0	0	0.040	0.181	1.048
TCGA-AQ-A7U7-01	0.113	0	0.039	0.045	0	0.385	0	0.054	0.026	0	0.021	0	0.018	0.027	0.136	0.099	0	0	0.036	0	0	0	0.010	0.351	0.943
TCGA-AR-A0TP-01	0.001	0.005	0	0	0	0.192	0	0.013	0.016	0	0.065	0	0	0.648	0	0.041	0	0	0.021	0	0	0	0.010	0.267	1.053
TCGA-AR-A0TQ-01	0.053	0	0.017	0.084	0	0.307	0.025	0	0	0	0.04	0	0.015	0.285	0.063	0.096	0	0	0.015	0	0	0	0.010	0.308	0.966
TCGA-AR-A0TR-01	0.134	0	0.052	0.002	0.032	0.272	0	0	0.089	0	0.077	0	0.027	0.012	0.075	0.144	0	0	0.084	0	0	0	1.000	-0.052	1.099
TCGA-AR-A0TS-01	0.219	0	0.008	0.03	0	0.371	0.006	0	0.007	0	0.071	0	0.038	0.053	0.089	0.108	0	0	0.001	0	0	0	0.010	0.427	0.904
TCGA-AR-A0TT-01	0.102	0	0.073	0.002	0	0.219	0.015	0.003	0.071	0	0.03	0.002	0.011	0.224	0.104	0.106	0	0	0.037	0	0	0	0.010	0.353	0.938
TCGA-AR-A0TV-01	0.072	0	0.02	0.068	0	0.206	0	0.041	0.033	0	0.02	0.012	0.04	0.238	0.075	0.146	0	0	0.028	0	0	0	0.070	0.116	1.048
TCGA-AR-A0TX-01	0.076	0	0.021	0	0	0.237	0	0.028	0.03	0	0.017	0.033	0.002	0.115	0.099	0.225	0.004	0	0.114	0	0	0	0.010	0.253	0.982
TCGA-AR-A0TY-01	0.13	0	0.088	0.029	0	0.141	0	0	0.003	0	0.012	0	0	0.254	0.014	0.247	0	0	0.082	0	0	0	0.010	0.301	0.965
TCGA-AR-A0TZ-01	0.044	0	0.115	0.038	0	0.283	0	0.038	0	0	0	0.044	0.035	0.046	0.022	0.276	0	0.017	0.043	0	0	0	0.800	-0.012	1.081
TCGA-AR-A0U2-01	0.038	0	0.109	0.1	0	0.323	0.007	0	0	0	0.024	0	0.015	0.169	0.065	0.123	0	0	0.026	0	0	0	0.070	0.115	1.042
TCGA-AR-A0U3-01	0.131	0	0.084	0	0	0.195	0	0	0.044	0	0.008	0.007	0	0.27	0.039	0.144	0	0.021	0.058	0	0	0	0.090	0.099	1.050
TCGA-AR-A0U4-01	0	0	0.001	0.006	0	0.12	0.005	0.006	0	0	0.019	0	0.069	0.598	0.137	0.029	0.003	0	0.001	0	0	0.006	0.000	0.614	0.793
TCGA-AR-A1AH-01	0.101	0	0.011	0.051	0	0.385	0	0.031	0.004	0	0	0	0.055	0.307	0.016	0	0	0	0	0.034	0.005	0	0.270	0.062	1.087
TCGA-AR-A1AI-01	0.01	0	0.127	0.014	0	0.322	0.017	0.016	0.002	0	0.004	0.038	0.06	0.107	0.096	0.074	0.011	0.039	0.06	0	0.001	0	0.010	0.258	0.977
TCGA-AR-A1AJ-01	0.056	0	0	0	0	0.334	0.002	0.074	0	0	0.056	0	0.02	0.084	0.139	0.215	0.003	0.017	0	0	0	0	0.010	0.350	0.944
TCGA-AR-A1AK-01	0.019	0	0.002	0	0	0.294	0	0.064	0	0	0	0.024	0.021	0.008	0.142	0.252	0.027	0	0.148	0	0	0	0.020	0.245	0.990
TCGA-AR-A1AL-01	0.112	0	0.013	0.033	0	0.38	0	0	0	0	0.032	0	0.033	0	0.025	0.224	0.071	0.007	0.069	0	0	0	0.330	0.048	1.063
TCGA-AR-A1AM-01	0.114	0	0.087	0.069	0	0.186	0	0.015	0.029	0	0.006	0.01	0.029	0.137	0.05	0.186	0	0	0.081	0	0	0	0.070	0.115	1.031
TCGA-AR-A1AN-01	0.015	0	0.003	0.02	0	0.143	0	0	0.003	0	0.017	0	0	0.558	0.011	0.206	0	0.004	0.019	0	0	0	0.000	0.625	0.782
TCGA-AR-A1AO-01	0.015	0	0	0	0	0.091	0	0.018	0.027	0	0.017	0	0	0.629	0.046	0.118	0	0	0.038	0	0	0	0.000	0.852	0.545
TCGA-AR-A1AP-01	0.096	0	0	0.019	0	0.239	0	0.024	0.043	0	0.042	0	0.02	0.132	0.08	0.254	0.009	0	0.042	0	0	0	0.090	0.105	1.047
TCGA-AR-A1AQ-01	0.033	0	0.128	0.003	0	0.163	0.027	0.078	0.01	0	0	0.03	0.015	0.238	0.102	0.125	0.003	0.003	0.042	0	0	0	0.010	0.336	0.946
TCGA-AR-A1AR-01	0.008	0	0	0	0	0.314	0.029	0	0	0	0.063	0	0.045	0.298	0.105	0.118	0.001	0	0.018	0	0	0	0.010	0.409	0.916
TCGA-AR-A1AS-01	0.005	0.005	0	0	0.028	0.24	0	0.005	0.05	0	0.073	0	0	0.393	0	0.162	0	0	0	0.035	0	0.003	0.030	0.199	1.041
TCGA-AR-A1AT-01	0.04	0	0.025	0.006	0	0.293	0	0.027	0.012	0	0.014	0.024	0.028	0.181	0.1	0.191	0.001	0.011	0.048	0	0	0	0.010	0.265	0.981
TCGA-AR-A1AU-01	0.096	0	0.096	0.095	0	0.197	0	0	0	0	0	0.045	0.044	0.065	0.039	0.2	0	0.007	0.115	0	0	0	0.640	0.010	1.066
TCGA-AR-A1AV-01	0.015	0	0.042	0	0	0.3	0	0.017	0.078	0	0.097	0	0.02	0.347	0	0.046	0	0	0.037	0	0	0	0.660	0.008	1.112
TCGA-AR-A1AW-01	0.093	0	0.099	0	0	0.303	0.042	0	0.009	0	0.076	0	0.02	0.139	0.09	0.112	0	0	0.017	0	0	0	0.010	0.434	0.901
TCGA-AR-A1AX-01	0.152	0	0	0.053	0	0.257	0.006	0	0.032	0	0.082	0	0.049	0.025	0.15	0.055	0.001	0	0.138	0	0	0	0.010	0.338	0.948
TCGA-AR-A1AY-01	0	0.006	0	0	0	0.257	0	0.019	0	0	0.052	0	0	0.579	0.02	0.059	0	0	0	0.009	0	0	0.040	0.158	1.103
TCGA-AR-A24H-01	0.027	0	0.047	0.02	0	0.133	0	0.133	0.001	0	0	0.024	0.037	0.112	0.119	0.273	0	0	0.065	0	0.009	0	0.060	0.133	1.036
TCGA-AR-A24K-01	0.146	0	0.014	0.029	0	0.27	0	0	0	0	0.03	0	0.041	0.01	0.039	0.363	0.008	0.046	0.005	0	0	0	1.000	-0.054	1.111
TCGA-AR-A24L-01	0.119	0	0.03	0	0	0.211	0	0	0.009	0	0	0.011	0	0.221	0.052	0.276	0	0	0.072	0	0	0	0.650	0.009	1.097
TCGA-AR-A24M-01	0.065	0	0.032	0	0	0.178	0	0	0.001	0	0.024	0	0	0.1	0.015	0.412	0	0.008	0.16	0	0	0.005	0.410	0.034	1.085
TCGA-AR-A24N-01	0.117	0	0.156	0.016	0	0.229	0	0.03	0.017	0	0.024	0.021	0.023	0.04	0.036	0.207	0.001	0.02	0.058	0	0.004	0	0.760	-0.008	1.067
TCGA-AR-A24O-01	0.022	0	0.013	0.134	0	0.196	0.017	0	0	0	0	0	0.051	0.09	0.104	0.221	0.017	0	0.128	0	0.006	0	0.300	0.058	1.065
TCGA-AR-A24P-01	0.092	0	0.045	0.004	0	0.322	0	0	0	0	0.035	0	0.046	0.023	0.065	0.304	0	0.012	0.052	0	0	0	0.720	0.001	1.081
TCGA-AR-A24Q-01	0.041	0	0.08	0.005	0	0.199	0	0	0.001	0.032	0	0.008	0	0.412	0.058	0.091	0	0	0.073	0	0	0	0.000	0.452	0.894
TCGA-AR-A24R-01	0.089	0	0.008	0.026	0	0.314	0	0.006	0.045	0	0.021	0.02	0.031	0.097	0.073	0.176	0.011	0.016	0.068	0	0	0	0.260	0.063	1.060
TCGA-AR-A24S-01	0.045	0	0	0.002	0	0.241	0.01	0	0	0	0.043	0	0.011	0.249	0.07	0.195	0.1	0	0.035	0	0	0	0.010	0.347	0.946
TCGA-AR-A24T-01	0.09	0	0.035	0.006	0	0.241	0	0	0	0	0.026	0	0.01	0	0.043	0.402	0.005	0	0.143	0	0	0	0.090	0.104	1.044
TCGA-AR-A24U-01	0.059	0	0.053	0.011	0	0.39	0.003	0	0	0	0.054	0	0.026	0.148	0.078	0.127	0	0.01	0.043	0	0	0	0.040	0.193	1.009
TCGA-AR-A24V-01	0.075	0	0	0	0	0.227	0	0	0.06	0	0.041	0	0	0.23	0.031	0.25	0	0	0.085	0	0	0	0.040	0.154	1.034
TCGA-AR-A24W-01	0.072	0	0.124	0.075	0	0.357	0	0	0	0	0.002	0	0.036	0	0	0.185	0	0.041	0.091	0	0.017	0	0.660	0.007	1.072
TCGA-AR-A24X-01	0.162	0	0.063	0.003	0	0.433	0	0.019	0	0	0	0.012	0.017	0	0.07	0.201	0	0.005	0	0.015	0	0	0.760	-0.008	1.096
TCGA-AR-A24Z-01	0.008	0	0.003	0	0	0.347	0	0.038	0.041	0	0.068	0	0	0.366	0.018	0.098	0	0	0.012	0	0	0	0.410	0.031	1.116
TCGA-AR-A250-01	0.068	0	0.03	0	0	0.235	0	0.012	0.052	0	0.018	0	0	0.241	0.062	0.221	0	0	0.06	0	0	0	0.010	0.313	0.960
TCGA-AR-A251-01	0.009	0	0.017	0.134	0	0.274	0	0.056	0.018	0	0	0	0.04	0.158	0.102	0.122	0.001	0.019	0.045	0	0.006	0	0.260	0.063	1.074
TCGA-AR-A252-01	0.025	0	0.089	0.034	0	0.488	0.006	0	0	0	0.037	0.008	0.053	0.037	0.057	0.105	0.007	0	0.053	0	0	0	0.030	0.220	1.000
TCGA-AR-A254-01	0.01	0	0.031	0.008	0	0.308	0	0	0	0	0.015	0.018	0.023	0.155	0.082	0.249	0.004	0	0.098	0	0	0	0.010	0.291	0.968
TCGA-AR-A255-01	0.1	0	0	0.008	0	0.268	0	0.051	0.005	0	0.017	0.002	0.012	0.115	0.099	0.189	0.006	0.021	0.106	0	0	0	0.070	0.116	1.040
TCGA-AR-A256-01	0.027	0	0	0	0	0.279	0	0.05	0	0	0.012	0	0	0.342	0.032	0.115	0	0.143	0	0	0	0	0.040	0.190	1.038
TCGA-AR-A2LE-01	0.144	0	0.026	0.017	0	0.279	0	0	0.013	0	0.026	0	0.011	0.051	0.045	0.269	0.051	0.023	0.044	0	0	0	0.950	-0.026	1.089
TCGA-AR-A2LH-01																									

	0.094	0	0.041	0.059	0	0.38	0	0	0.038	0	0	0.039	0.085	0.019	0.066	0.11	0	0	0.07	0	0	0	0.030	0.200	1.007
TCGA-AR-A2LJ-01	0.02	0	0.009	0.024	0	0.143	0	0.003	0.052	0	0	0.028	0.023	0.387	0.042	0.216	0	0	0.053	0	0	0	0.000	0.537	0.843
TCGA-AR-A2LK-01	0.071	0	0.015	0.007	0	0.165	0	0.008	0.037	0	0.004	0.003	0.028	0.14	0.064	0.336	0	0	0.121	0	0	0	0.350	0.042	1.078
TCGA-AR-A2LL-01	0.013	0	0.016	0	0	0.205	0	0.005	0.045	0	0	0	0.03	0.306	0	0.215	0.003	0.016	0.146	0	0	0	0.040	0.194	1.027
TCGA-AR-A2LM-01	0.102	0	0.014	0.046	0	0.371	0	0	0	0	0.014	0	0.063	0	0.04	0.191	0.018	0.016	0.124	0	0	0	0.310	0.052	1.061
TCGA-AR-A2LN-01	0.194	0	0.093	0.003	0	0.317	0	0	0	0	0.008	0.012	0.031	0	0.033	0.185	0	0.006	0.118	0	0	0	0.390	0.038	1.059
TCGA-AR-A2LO-01	0.151	0	0.058	0.117	0	0.325	0	0.036	0.003	0	0.03	0	0.026	0	0.073	0.145	0.008	0.006	0.02	0	0	0	0.020	0.222	0.999
TCGA-AR-A2LQ-01	0.048	0	0.073	0	0	0.255	0	0	0.001	0	0.021	0.027	0.038	0.255	0.013	0.222	0	0	0.049	0	0	0	0.040	0.154	1.033
TCGA-AR-A2LR-01	0.157	0	0.025	0	0	0.319	0	0.053	0	0	0.002	0.008	0.089	0.085	0.012	0.211	0	0	0.029	0	0.009	0	0.720	0.001	1.083
TCGA-AR-A5QM-01	0.106	0	0.038	0.008	0	0.172	0	0.014	0.026	0	0.013	0	0	0.347	0.023	0.122	0	0.017	0.115	0	0	0	0.030	0.199	1.019
TCGA-AR-A5QN-01	0.101	0	0.018	0.006	0	0.412	0	0	0	0	0.032	0	0.01	0	0.1	0.246	0	0	0.075	0	0	0	0.350	0.043	1.074
TCGA-AR-A5QP-01	0.126	0	0.154	0.021	0	0.227	0	0.004	0.012	0	0.022	0.005	0.016	0.064	0.032	0.287	0	0.003	0.027	0	0	0	0.200	0.078	1.043
TCGA-AR-A5QQ-01	0.068	0	0.043	0.02	0	0.304	0.003	0	0.007	0	0.026	0.006	0	0.178	0.068	0.254	0	0.015	0	0.009	0	0	0.000	0.455	0.890
TCGA-B6-A0I1-01	0.157	0	0.074	0.023	0	0.007	0	0.017	0.048	0	0	0	0	0.529	0.052	0.086	0	0.006	0	0	0	0	0.070	0.113	1.109
TCGA-B6-A0I2-01	0.056	0	0.032	0.135	0	0.104	0.007	0.089	0.058	0	0.034	0.056	0.01	0.14	0.192	0.031	0	0	0.056	0	0	0	0.010	0.421	0.911
TCGA-B6-A0I5-01	0.089	0	0.044	0.028	0	0.124	0	0.071	0.058	0	0.011	0	0.014	0.237	0.029	0.257	0	0.004	0.034	0	0	0	0.310	0.054	1.076
TCGA-B6-A0I6-01	0.064	0	0.001	0.04	0	0.166	0	0.072	0.039	0	0	0.037	0.011	0.337	0.055	0.125	0	0	0.053	0	0	0	0.210	0.073	1.080
TCGA-B6-A0I8-01	0.091	0	0.128	0	0	0.083	0	0	0.056	0	0	0.034	0.001	0.096	0.02	0.374	0.003	0	0.115	0	0	0	0.880	-0.018	1.091
TCGA-B6-A0I9-01	0.119	0	0.07	0.04	0	0.165	0	0.016	0.039	0	0.027	0	0.018	0.166	0.038	0.194	0	0.084	0.025	0	0	0	0.350	0.045	1.064
TCGA-B6-A0IA-01	0.097	0	0	0	0	0.014	0	0.112	0.015	0.042	0.006	0	0	0.432	0	0.161	0	0.122	0	0	0	0	0.010	0.252	1.019
TCGA-B6-A0IB-01	0.111	0	0.04	0.032	0	0.166	0	0	0.092	0	0	0.064	0	0.247	0.046	0.143	0	0	0.059	0	0	0	0.870	-0.018	1.100
TCGA-B6-A0IC-01	0	0.01	0.003	0	0	0.422	0	0.04	0.067	0	0.017	0.026	0.105	0.238	0	0.015	0.006	0	0.041	0	0.009	0	0.910	-0.023	1.119
TCGA-B6-A0IE-01	0.07	0	0.417	0.024	0	0.114	0	0	0.047	0	0	0.036	0.011	0.007	0.053	0.134	0.005	0	0.081	0	0	0	0.280	0.060	1.042
TCGA-B6-A0IG-01	0.181	0	0.183	0.048	0	0.15	0	0.023	0.021	0	0	0	0	0.265	0	0	0	0.087	0.041	0	0	0	0.060	0.132	1.030
TCGA-B6-A0IH-01	0.072	0	0.148	0.055	0	0.281	0	0	0.023	0	0.02	0.003	0.031	0	0.098	0.171	0.007	0	0.092	0	0	0	0.070	0.108	1.032
TCGA-B6-A0IJ-01	0.031	0	0.082	0	0	0.209	0.004	0.04	0.02	0	0.008	0.021	0.052	0.338	0.09	0.06	0	0	0.045	0	0	0	0.010	0.342	0.948
TCGA-B6-A0IK-01	0.126	0	0.094	0.06	0	0.255	0	0.012	0.037	0	0.033	0	0.018	0.038	0.053	0.189	0.006	0.054	0.024	0	0	0	0.110	0.096	1.039
TCGA-B6-A0IM-01	0.001	0.005	0.002	0	0	0.287	0	0.013	0.033	0	0.072	0	0.024	0.393	0.015	0.079	0	0	0.078	0	0	0	0.130	0.094	1.083
TCGA-B6-A0IN-01	0.128	0	0.045	0.001	0	0.253	0	0.016	0.053	0	0.051	0	0.029	0.129	0.054	0.145	0.066	0	0.03	0	0	0	0.040	0.149	1.019
TCGA-B6-A0IO-01	0.048	0	0	0.035	0	0.257	0	0.031	0.072	0	0	0.004	0.025	0.189	0.119	0.161	0	0	0.062	0	0	0	0.310	0.051	1.078
TCGA-B6-A0IP-01	0.078	0.091	0	0.071	0	0.203	0	0	0.049	0	0.02	0	0.04	0.165	0.064	0.034	0.001	0	0.184	0	0	0	0.430	0.029	1.071
TCGA-B6-A0IQ-01	0.078	0	0.096	0.031	0	0.256	0	0.087	0.045	0	0.013	0.019	0	0.18	0.124	0.045	0.015	0	0.01	0	0	0	0.050	0.144	1.034
TCGA-B6-A0RE-01	0.036	0	0.118	0.016	0	0.082	0	0.066	0.039	0	0.007	0.001	0	0.368	0.099	0.133	0	0.013	0.022	0	0	0	0.180	0.084	1.082
TCGA-B6-A0RG-01	0.084	0	0.06	0	0	0.115	0	0.054	0.017	0	0	0.046	0.017	0.336	0.031	0.137	0	0.001	0.102	0	0	0	0.410	0.034	1.090
TCGA-B6-A0RH-01	0.095	0	0.11	0.072	0	0.088	0	0.049	0.053	0	0.004	0.02	0	0.165	0.079	0.138	0	0.064	0.065	0	0	0	0.090	0.103	1.038
TCGA-B6-A0RI-01	0.07	0	0.059	0.008	0	0.136	0	0.007	0.028	0	0.012	0.01	0.002	0.272	0.038	0.277	0	0	0.08	0	0	0	0.110	0.097	1.065
TCGA-B6-A0RL-01	0.073	0	0.046	0.057	0	0.009	0	0.132	0.025	0	0	0.021	0.001	0.294	0	0.191	0	0.111	0.039	0	0	0	0.980	-0.038	1.122
TCGA-B6-A0RM-01	0.119	0	0.041	0.048	0	0.238	0	0.002	0.017	0	0.006	0.002	0.007	0	0.042	0.195	0.048	0	0.237	0	0	0	0.720	-0.001	1.079
TCGA-B6-A0RN-01	0.112	0	0.141	0.061	0	0.275	0	0	0.014	0	0	0.033	0.013	0	0.036	0.208	0	0	0.106	0	0	0	0.910	-0.023	1.078
TCGA-B6-A0RO-01	0.126	0	0.288	0.047	0	0.112	0	0.023	0.006	0	0	0.049	0.008	0.007	0.038	0.267	0	0	0.029	0	0	0	0.760	-0.007	1.067
TCGA-B6-A0RP-01	0.097	0	0.027	0.105	0	0.262	0	0.021	0.016	0	0.056	0	0.029	0.033	0.046	0.283	0.019	0	0.006	0	0	0	0.290	0.058	1.066
TCGA-B6-A0RQ-01	0.173	0	0.065	0.063	0	0.067	0	0.045	0.001	0	0.019	0.014	0.004	0.033	0.014	0.4	0	0	0.101	0	0	0	0.620	0.013	1.079
TCGA-B6-A0RS-01	0.079	0	0.032	0.043	0	0.336	0.003	0.034	0.004	0	0.029	0	0.006	0.095	0.065	0.235	0	0.026	0.012	0	0	0	0.410	0.032	1.079
TCGA-B6-A0RT-01	0.084	0	0.017	0.082	0	0.359	0.043	0	0.045	0	0.066	0	0.027	0.058	0.154	0.039	0.002	0	0.026	0	0	0	0.000	0.507	0.861
TCGA-B6-A0RU-01	0.059	0	0.016	0.018	0	0.235	0	0.045	0	0	0	0.039	0.024	0.24	0.074	0.214	0	0	0.035	0	0	0	0.040	0.170	1.029
TCGA-B6-A0RV-01	0.094	0	0.045	0.107	0	0.329	0	0	0	0	0.039	0	0.045	0.118	0.076	0.109	0	0	0.039	0	0	0	0.070	0.125	1.039
TCGA-B6-A0WS-01	0.167	0	0.043	0	0.005	0.196	0.013	0	0.088	0	0.041	0	0.022	0.232	0.079	0.07	0	0	0.042	0	0	0	0.040	0.156	1.024
TCGA-B6-A0WT-01	0.1	0	0.182	0	0	0.201	0	0.024	0.028	0	0	0.031	0	0.104	0.018	0.196	0	0.013	0.103	0	0	0	0.600	0.015	1.059
TCGA-B6-A0WV-01	0.202	0	0.284	0	0	0.15	0	0.007	0.024	0	0	0.031	0	0.146	0.014	0.054	0	0.024	0.063	0	0	0	1.000	-0.065	1.088
TCGA-B6-A0WW-01	0.072	0	0.11	0	0	0.171	0	0.051	0.025	0	0.017	0	0	0.101	0.13	0.255	0	0	0.067	0	0	0	0.340	0.047	1.066
TCGA-B6-A0WX-01	0.158	0	0.051	0.067	0	0.329	0	0.023	0.042	0	0.028	0	0.024	0.039	0.095	0.14	0	0	0.003	0	0	0	0.050	0.143	1.034
TCGA-B6-A0WY-01	0.104	0	0.065	0.033	0	0.159	0	0.051	0.03	0	0	0.039	0.017	0.168	0.096	0.185	0	0	0.053	0	0	0	0.060	0.130	1.032
TCGA-B6-A0WZ-01	0.075	0	0.02	0.005	0	0.15	0	0.026	0.041	0	0.007	0.027	0	0.248	0.026	0.223	0	0.039	0.114	0	0	0	0.700	0.004	1.096
TCGA-B6-A0X0-01	0.098	0	0.043	0.099	0	0.275	0	0.019	0.041	0	0.01	0.01	0.014	0.096	0.065	0.18	0.029	0.008	0.013	0	0	0	0.890	-0.019	1.095
TCGA-B6-A0X4-01	0	0	0.361	0	0	0.152	0	0.01	0.039	0	0	0.076	0.009	0.044	0.002	0.188	0	0.001	0.118	0	0	0	0.990	-0.041	1.078
TCGA-B6-A0X5-01	0.131	0	0.07	0.057	0	0.137	0	0.027	0	0	0	0.046	0.017	0	0	0.375	0	0	0.14	0	0	0	0.980	-0.040	1.096
TCGA-B6-A0X7-01	0.143	0	0.108	0.018	0	0.204	0	0.015	0.016	0	0.016	0.016	0.008	0.005	0.062	0.232	0.005	0.004	0.148	0	0	0	0.660	0.008	1.065

TCGA-B6-A1KC-01	0.06	0	0.035	0.012	0	0.064	0	0.007	0.023	0	0	0	0	0.185	0.031	0.484	0	0	0.1	0	0	0	0.030	0.200	1.034
TCGA-B6-A1KF-01	0.001	0.005	0.017	0	0	0.238	0	0.033	0.035	0	0.078	0	0.02	0.525	0	0.033	0	0	0.016	0	0	0	0.040	0.183	1.067
TCGA-B6-A1KI-01	0.16	0	0.135	0	0	0.06	0	0.036	0.031	0.023	0	0.03	0	0.306	0.023	0.119	0	0	0.077	0	0	0	0.720	0.001	1.091
TCGA-B6-A1KN-01	0.208	0	0.027	0.134	0.027	0.223	0	0	0	0	0	0	0.025	0.085	0.04	0.018	0	0.099	0.109	0	0	0.004	0.030	0.221	0.997
TCGA-B6-A2IU-01	0.161	0	0.116	0.051	0	0.181	0	0.015	0.007	0	0.007	0.004	0.05	0	0.062	0.242	0.015	0	0.09	0	0	0	0.740	-0.005	1.069
TCGA-B6-A3ZX-01	0.357	0	0.014	0.129	0	0.139	0.008	0.067	0.073	0	0.013	0	0.006	0.073	0.034	0.072	0	0.011	0.004	0	0	0	0.000	0.533	0.847
TCGA-B6-A400-01	0.008	0	0	0	0	0.108	0	0.04	0.038	0	0.023	0	0	0.473	0	0.19	0	0.096	0	0.025	0	0	0.000	0.463	0.899
TCGA-B6-A401-01	0.087	0	0.003	0	0	0.119	0	0.127	0.049	0	0.05	0	0	0.505	0	0.023	0	0.019	0.019	0	0	0	0.000	0.708	0.719
TCGA-B6-A402-01	0.002	0.009	0.003	0	0	0.192	0	0.081	0.015	0	0.099	0	0	0.588	0.008	0.003	0	0	0	0	0	0	0.020	0.237	1.052
TCGA-B6-A408-01	0.089	0	0.075	0.01	0	0.223	0	0.032	0.078	0	0.024	0.009	0.008	0.165	0.033	0.211	0	0.009	0.034	0	0	0	0.040	0.146	1.024
TCGA-B6-A409-01	0.061	0	0.073	0.043	0	0.231	0	0.012	0.089	0	0	0.03	0.019	0.108	0.081	0.208	0	0.01	0.037	0	0	0	0.220	0.072	1.054
TCGA-B6-A40B-01	0.001	0	0.007	0	0	0.333	0	0.075	0.018	0	0	0.025	0.021	0.36	0	0.025	0	0.022	0.113	0	0.001	0	0.070	0.117	1.065
TCGA-B6-A40C-01	0.089	0	0.093	0.085	0	0.231	0	0.005	0.052	0	0.028	0	0.092	0	0.043	0.272	0	0	0.009	0	0	0	0.310	0.056	1.057
TCGA-BH-A0AU-01	0.099	0	0.088	0.036	0	0.25	0	0.023	0.028	0	0.038	0	0.033	0.158	0.068	0.127	0	0	0.051	0	0	0	0.070	0.124	1.030
TCGA-BH-A0AU-11	0.109	0	0.064	0.088	0	0.423	0	0	0	0	0.026	0.028	0.026	0.002	0	0.175	0	0.04	0.019	0	0	0	0.310	0.056	1.075
TCGA-BH-A0AV-01	0.056	0	0.028	0	0	0.111	0	0.004	0.011	0	0.027	0	0	0.598	0.011	0.123	0	0.009	0.021	0	0	0	0.000	0.494	0.888
TCGA-BH-A0AW-01	0.082	0	0.034	0.042	0	0.303	0.032	0.015	0.046	0	0.016	0.023	0.03	0.125	0.083	0.095	0.003	0	0.071	0	0	0	0.010	0.359	0.935
TCGA-BH-A0AY-01	0.058	0	0.055	0.044	0	0.233	0	0.003	0.016	0	0	0.032	0.017	0.069	0.054	0.284	0	0	0.134	0	0	0	0.020	0.237	0.985
TCGA-BH-A0AY-11	0.1	0	0.202	0.045	0	0.214	0	0	0	0	0	0.04	0.03	0.021	0.013	0.245	0	0.006	0.076	0	0.007	0	0.310	0.052	1.045
TCGA-BH-A0AZ-01	0.134	0	0.05	0.013	0	0.239	0	0	0.035	0	0.007	0.007	0.001	0.18	0.048	0.215	0	0	0.071	0	0	0	0.090	0.105	1.043
TCGA-BH-A0AZ-11	0.137	0	0.069	0.037	0	0.341	0	0	0	0	0.023	0.018	0.042	0.048	0	0.246	0	0.016	0.023	0	0	0	0.370	0.040	1.067
TCGA-BH-A0B0-01	0.092	0	0.006	0.078	0	0.222	0.01	0.022	0.038	0	0.028	0	0.022	0.145	0.061	0.128	0.005	0.033	0.109	0	0	0	0.040	0.151	1.022
TCGA-BH-A0B1-01	0.124	0	0.017	0.002	0	0.335	0	0.044	0	0	0.015	0.005	0.004	0.049	0.103	0.208	0.007	0.005	0.084	0	0	0	0.660	0.006	1.083
TCGA-BH-A0B2-01	0.136	0	0	0.021	0	0.225	0	0.003	0.009	0	0.024	0.018	0.029	0.223	0.045	0.222	0	0	0.045	0	0	0	0.020	0.236	0.995
TCGA-BH-A0B3-01	0.041	0	0.171	0.056	0	0.134	0.003	0.045	0.045	0	0.033	0	0.007	0.098	0.175	0.163	0	0	0.029	0	0	0	0.010	0.301	0.963
TCGA-BH-A0B3-11	0.072	0	0.068	0.135	0	0.275	0	0.021	0	0	0	0.017	0.057	0.018	0.04	0.129	0	0	0.167	0	0.001	0	0.210	0.075	1.051
TCGA-BH-A0B4-01	0.106	0	0	0.044	0	0.255	0	0.009	0.045	0	0.041	0	0.02	0.082	0.109	0.202	0	0	0.088	0	0	0	0.040	0.176	1.015
TCGA-BH-A0B5-01	0.081	0	0.025	0.011	0	0.356	0	0.008	0.002	0	0.011	0.012	0.009	0.135	0.059	0.169	0	0.017	0.104	0	0	0	0.070	0.119	1.037
TCGA-BH-A0B5-11	0.056	0	0	0	0	0.201	0	0	0	0	0	0.083	0.091	0	0.021	0.398	0	0.001	0.132	0	0.017	0	0.720	-0.002	1.094
TCGA-BH-A0B6-01	0.048	0	0.031	0.168	0	0.197	0	0.012	0.092	0	0.012	0.055	0.073	0.099	0.107	0.051	0.003	0	0.053	0	0	0	0.010	0.391	0.922
TCGA-BH-A0B7-01	0.064	0	0.002	0.053	0	0.381	0.021	0	0	0	0.031	0	0	0.25	0.088	0.093	0	0	0.017	0	0	0	0.010	0.309	0.966
TCGA-BH-A0B7-11	0.101	0	0.109	0.063	0	0.368	0	0	0	0	0.052	0.008	0.019	0.031	0	0.225	0	0.021	0	0.002	0	0	0.340	0.048	1.068
TCGA-BH-A0B8-01	0.131	0	0.07	0	0	0.3	0	0.003	0.001	0	0.017	0	0.028	0.009	0.055	0.279	0.02	0.009	0.077	0	0	0	0.890	-0.020	1.081
TCGA-BH-A0B9-01	0.065	0	0.006	0.105	0	0.226	0.026	0.06	0.079	0	0.013	0	0.009	0.099	0.141	0.087	0.056	0	0.029	0	0	0	0.010	0.328	0.953
TCGA-BH-A0BA-01	0.16	0	0.113	0.013	0	0.401	0	0	0	0	0.004	0.014	0.02	0	0.037	0.126	0.016	0	0.096	0	0	0	0.190	0.080	1.048
TCGA-BH-A0BC-01	0.065	0	0.01	0.059	0	0.171	0	0.007	0.063	0	0.027	0	0	0.252	0.073	0.245	0	0	0.028	0	0	0	0.040	0.185	1.025
TCGA-BH-A0BC-11	0.007	0	0.058	0.153	0	0.26	0	0.009	0	0	0	0.005	0.087	0.057	0.014	0.164	0	0.042	0.137	0	0.008	0	0.260	0.063	1.055
TCGA-BH-A0BD-01	0.062	0	0.004	0.077	0	0.311	0	0.032	0.015	0	0.017	0.004	0.024	0.058	0.056	0.183	0	0.073	0.083	0	0	0	0.050	0.140	1.030
TCGA-BH-A0BF-01	0.096	0	0.1	0.034	0	0.232	0.012	0.048	0.054	0	0.018	0.009	0.017	0.041	0.084	0.146	0	0.001	0.109	0	0	0	0.010	0.335	0.943
TCGA-BH-A0BG-01	0.041	0	0	0.007	0	0.425	0	0.049	0.025	0	0.017	0.037	0.026	0.047	0.166	0.066	0	0.052	0.042	0	0	0	0.010	0.269	0.994
TCGA-BH-A0BJ-01	0.098	0	0.045	0.035	0	0.22	0	0.009	0.002	0	0	0.029	0.013	0.206	0.031	0.244	0	0	0.067	0	0	0	0.040	0.179	1.017
TCGA-BH-A0BJ-11	0.167	0	0.05	0.059	0	0.256	0	0.01	0.008	0	0	0.027	0.041	0	0	0.279	0	0.019	0.083	0	0	0	0.590	0.017	1.071
TCGA-BH-A0BL-01	0.088	0	0	0.016	0	0.282	0	0.073	0.006	0	0.029	0.019	0.03	0.059	0.148	0.155	0.038	0.014	0.04	0	0	0	0.010	0.315	0.959
TCGA-BH-A0BM-01	0.149	0	0.095	0.054	0	0.296	0	0.002	0	0	0	0.037	0.023	0.046	0.071	0.141	0	0	0.087	0	0	0	0.720	-0.003	1.077
TCGA-BH-A0BO-01	0.052	0	0.043	0	0	0.232	0.002	0	0	0	0.049	0	0.117	0.025	0.013	0.218	0	0.027	0.09	0.001	0	0.132	0.220	0.069	1.058
TCGA-BH-A0BP-01	0.003	0.004	0.012	0	0	0.186	0	0.019	0.049	0	0.057	0	0	0.448	0.021	0.176	0	0	0.026	0	0	0	0.040	0.177	1.062
TCGA-BH-A0BQ-01	0.061	0	0.048	0.086	0	0.277	0	0	0	0	0.033	0	0.008	0.147	0.062	0.173	0	0.035	0.069	0	0	0	0.040	0.162	1.020
TCGA-BH-A0BQ-11	0.096	0	0.071	0.025	0	0.443	0	0	0	0	0	0.029	0.024	0.027	0.018	0.209	0	0.014	0.044	0	0	0	0.190	0.080	1.056
TCGA-BH-A0BR-01	0.043	0	0	0.02	0	0.189	0	0.026	0.033	0	0.038	0	0	0.351	0.04	0.2	0	0.021	0.039	0	0	0	0.010	0.279	0.989
TCGA-BH-A0BS-11	0.157	0	0.076	0.001	0	0.45	0	0	0	0	0.035	0.022	0.025	0.031	0	0.18	0	0.024	0	0	0	0	0.110	0.098	1.052
TCGA-BH-A0BT-01	0.082	0	0.025	0.079	0	0.263	0	0.037	0.068	0	0	0.015	0.019	0.087	0.07	0.168	0.015	0.019	0.053	0	0	0	0.150	0.089	1.050
TCGA-BH-A0BT-11	0.12	0	0.033	0.023	0	0.388	0	0	0	0	0.036	0	0.029	0.028	0.004	0.283	0	0.054	0	0	0.004	0	0.620	0.012	1.085
TCGA-BH-A0BV-01	0.123	0	0	0.028	0	0.275	0	0.006	0.027	0	0.042	0	0.003	0.22	0.029	0.223	0	0	0	0.023	0	0	0.110	0.096	1.061
TCGA-BH-A0BV-11	0.13	0	0.128	0	0	0.154	0	0.071	0	0	0	0.028	0.021	0	0.003	0.392	0	0.047	0.016	0.008	0	0	0.710	0.002	1.078
TCGA-BH-A0BW-01	0.072	0	0.018	0.008	0	0.244	0.007	0.039	0.013	0	0.084	0	0.02	0.07	0.254	0.121	0	0	0.051	0	0	0	0.010	0.429	0.910
TCGA-BH-A0BW-11	0.203	0	0.026	0.058	0	0.386	0.012	0	0	0	0.048	0	0.015	0.093	0.046	0.074	0.016	0.023	0	0	0	0	0.010	0.304	0.964
TCGA-BH-A0BZ-01	0.032	0	0.042	0.011	0	0.352	0	0	0.048	0	0	0.029	0.021	0.127	0.178	0.095	0.005	0	0.061	0	0	0	0.010	0.376	0.932

TCGA-BH-A0BZ-11	0.146	0	0.064	0	0	0.286	0	0.003	0	0	0.014	0.038	0.103	0	0.001	0.279	0	0.013	0	0.045	0.007	0	0.410	0.033	1.067
TCGA-BH-A0C0-01	0.061	0	0.026	0.078	0	0.228	0.009	0.017	0.076	0	0.033	0.02	0.03	0.089	0.146	0.123	0.009	0	0.054	0	0	0	0.010	0.386	0.924
TCGA-BH-A0C0-11	0.086	0	0.111	0.026	0	0.286	0	0.029	0	0	0.01	0.041	0.055	0	0.017	0.273	0	0.025	0.04	0	0	0	0.710	0.002	1.073
TCGA-BH-A0C1-01	0.116	0	0.02	0.121	0	0.429	0.004	0	0	0	0.025	0	0.017	0.008	0.047	0.15	0.021	0.023	0.019	0	0	0	0.180	0.086	1.068
TCGA-BH-A0C3-01	0.085	0	0	0.039	0	0.4	0	0	0	0	0.046	0	0.045	0.028	0.101	0.145	0.018	0.002	0.091	0	0	0	0.040	0.172	1.020
TCGA-BH-A0C3-11	0.088	0	0.136	0.063	0	0.367	0	0	0	0	0.044	0.007	0.02	0.03	0	0.174	0	0.015	0.056	0	0	0	0.190	0.082	1.048
TCGA-BH-A0C7-01	0.12	0	0.019	0.021	0	0.383	0	0	0	0	0.025	0	0	0.109	0.063	0.147	0	0.012	0.103	0	0	0	0.760	-0.008	1.089
TCGA-BH-A0DD-01	0.1	0	0	0.013	0	0.318	0	0.042	0.027	0	0.045	0	0.02	0.176	0.107	0.129	0	0	0.025	0	0	0	0.380	0.040	1.081
TCGA-BH-A0DD-11	0.003	0	0.001	0	0	0.286	0	0.001	0.009	0	0.029	0.076	0.04	0.165	0.014	0.298	0	0	0.078	0	0	0	0.410	0.031	1.091
TCGA-BH-A0DE-01	0.074	0	0.008	0.001	0	0.281	0	0.018	0.012	0	0.043	0	0.045	0	0.102	0.362	0	0.01	0.045	0	0	0	0.120	0.095	1.054
TCGA-BH-A0DG-01	0.114	0	0.059	0.002	0	0.267	0	0.015	0.023	0	0.065	0	0.021	0.134	0.078	0.182	0	0	0.04	0	0	0	0.040	0.174	1.012
TCGA-BH-A0DG-11	0.074	0	0.064	0.034	0	0.436	0	0	0	0	0.058	0.003	0.026	0.033	0.001	0.233	0	0.018	0.021	0	0	0	0.210	0.073	1.063
TCGA-BH-A0DH-01	0	0	0.239	0	0	0.095	0	0	0.071	0	0	0.023	0	0.362	0.039	0.092	0.004	0	0.075	0	0	0	0.010	0.268	0.984
TCGA-BH-A0DI-01	0.079	0	0.072	0	0	0.157	0	0	0.035	0	0.011	0	0	0.332	0.067	0.207	0	0	0.042	0	0	0	0.010	0.280	0.984
TCGA-BH-A0DK-01	0.091	0	0.03	0.088	0	0.339	0.013	0	0.035	0	0.028	0	0.009	0.097	0.104	0.148	0	0	0.018	0	0	0	0.010	0.347	0.943
TCGA-BH-A0DK-11	0.092	0	0.077	0.002	0	0.257	0	0.001	0	0	0.028	0	0.011	0.139	0.001	0.336	0	0.025	0.019	0.011	0	0	0.340	0.047	1.072
TCGA-BH-A0DL-01	0	0	0.027	0.06	0	0.133	0.038	0.028	0.029	0	0.022	0.002	0.011	0.219	0.141	0.191	0.067	0.01	0.021	0	0	0.002	0.010	0.307	0.968
TCGA-BH-A0DL-11	0.119	0	0.01	0.053	0	0.315	0	0.005	0.001	0	0.059	0	0.04	0	0.071	0.259	0.006	0.02	0.041	0	0	0	0.120	0.095	1.049
TCGA-BH-A0DO-01	0.089	0	0.073	0	0	0.257	0	0.085	0	0	0.045	0	0	0.383	0	0	0	0	0.067	0	0	0	0.240	0.066	1.081
TCGA-BH-A0DO-11	0.12	0	0.146	0.073	0	0.363	0	0	0	0	0.055	0	0.017	0.044	0.027	0.142	0	0	0	0.014	0	0	0.310	0.053	1.063
TCGA-BH-A0DP-01	0.123	0	0.051	0.072	0	0.294	0	0.005	0	0	0.005	0	0.035	0.042	0.062	0.254	0	0	0.055	0	0	0.003	0.120	0.095	1.044
TCGA-BH-A0DP-11	0.104	0	0.112	0.011	0	0.366	0	0	0	0	0.011	0.036	0.043	0	0	0.211	0	0.032	0.074	0	0	0	0.240	0.065	1.050
TCGA-BH-A0DQ-01	0.06	0	0.048	0	0	0.089	0	0.018	0.023	0	0	0.016	0	0.357	0.045	0.226	0	0	0.12	0	0	0	0.030	0.217	1.022
TCGA-BH-A0DQ-11	0.143	0	0.111	0.015	0	0.364	0	0	0	0	0.067	0	0.051	0.037	0.012	0.14	0	0.003	0.058	0	0	0	0.500	0.022	1.068
TCGA-BH-A0DS-01	0.071	0	0.02	0.076	0	0.159	0	0.02	0.025	0	0.017	0	0	0.265	0.05	0.274	0.008	0.002	0.013	0	0	0	0.010	0.282	0.982
TCGA-BH-A0DT-01	0.083	0	0.028	0.051	0	0.389	0	0	0	0	0.031	0	0.025	0.078	0.096	0.107	0.06	0.012	0.042	0	0	0	0.070	0.113	1.044
TCGA-BH-A0DT-11	0.156	0	0.1	0.033	0	0.277	0	0	0	0	0.025	0.023	0.036	0.03	0	0.295	0	0.026	0	0.001	0	0	0.620	0.012	1.075
TCGA-BH-A0DV-01	0.087	0	0.027	0.06	0	0.238	0	0	0	0	0	0.047	0.015	0.046	0.021	0.265	0	0	0.195	0	0	0	0.280	0.060	1.059
TCGA-BH-A0DV-11	0.092	0	0.142	0.038	0	0.389	0	0	0	0	0.019	0.028	0.032	0.021	0	0.186	0	0.015	0.039	0	0	0	0.410	0.034	1.066
TCGA-BH-A0DX-01	0.043	0	0.006	0.01	0	0.095	0.001	0.001	0.009	0	0.002	0	0	0.592	0.034	0.132	0	0	0.073	0	0	0	0.000	0.538	0.854
TCGA-BH-A0DZ-01	0.027	0	0.125	0.023	0	0.154	0	0.017	0.028	0	0.017	0	0	0.132	0.102	0.331	0	0.005	0.04	0	0	0	0.020	0.240	0.991
TCGA-BH-A0DZ-11	0	0	0.067	0.127	0	0.313	0	0.015	0	0	0	0	0.04	0.208	0.019	0.026	0.001	0	0.183	0	0	0	0.190	0.079	1.062
TCGA-BH-A0E0-01	0.017	0	0.003	0	0	0.301	0	0.076	0.006	0	0	0.041	0.055	0.053	0	0.132	0.102	0.083	0.122	0	0.009	0	0.280	0.061	1.055
TCGA-BH-A0E1-01	0.08	0	0.051	0	0	0.148	0	0	0	0	0	0.003	0	0.43	0	0.215	0	0.018	0.055	0	0	0	0.000	0.550	0.836
TCGA-BH-A0E1-11	0.058	0	0.231	0.084	0	0.277	0	0	0	0	0.035	0.018	0.026	0.003	0	0.212	0	0.056	0	0	0	0	0.200	0.078	1.044
TCGA-BH-A0E2-01	0.054	0	0.061	0.029	0	0.216	0	0.03	0.063	0	0.024	0	0	0.257	0.068	0.121	0	0	0.077	0	0	0	0.090	0.099	1.051
TCGA-BH-A0E6-01	0.127	0	0.171	0.025	0	0.066	0.018	0.029	0.054	0	0	0	0	0.437	0.04	0.014	0	0.001	0.017	0	0	0	0.030	0.204	1.022
TCGA-BH-A0E7-01	0.033	0	0.087	0	0	0.186	0	0.014	0.011	0	0.006	0.057	0.017	0.025	0.009	0.369	0	0.008	0.177	0	0	0	0.820	-0.015	1.089
TCGA-BH-A0E9-01	0.128	0	0.036	0.062	0	0.372	0	0	0	0	0.019	0	0.023	0	0.03	0.218	0.041	0.011	0.06	0	0	0	0.310	0.054	1.062
TCGA-BH-A0EA-01	0.078	0	0.023	0.026	0	0.155	0	0.019	0.021	0	0.022	0.001	0.007	0.23	0.032	0.273	0.078	0.006	0.03	0	0	0	0.070	0.116	1.055
TCGA-BH-A0EE-01	0.075	0	0.074	0.034	0	0.241	0	0.034	0.044	0	0.065	0	0.013	0.063	0.156	0.104	0	0.053	0.043	0	0	0	0.030	0.208	1.008
TCGA-BH-A0EI-01	0.088	0	0.004	0	0	0.196	0	0.011	0.031	0	0	0.033	0	0.134	0.007	0.351	0	0.009	0.136	0	0	0	0.760	-0.008	1.099
TCGA-BH-A0GY-01	0.064	0	0	0	0	0.172	0	0.02	0.012	0	0.044	0	0	0.398	0.076	0.126	0	0	0.085	0	0	0.001	0.020	0.226	1.019
TCGA-BH-A0GZ-01	0.131	0	0.065	0.037	0	0.175	0	0.022	0.045	0	0.009	0.016	0.006	0.059	0.033	0.297	0.012	0	0.094	0	0	0	0.720	0.001	1.074
TCGA-BH-A0H0-01	0.039	0	0.089	0	0	0.248	0	0.035	0.029	0	0.034	0.011	0.035	0	0.037	0.215	0	0.009	0.22	0	0	0	0.450	0.024	1.065
TCGA-BH-A0H3-01	0.106	0	0.019	0.046	0	0.34	0	0	0	0	0.009	0.005	0.005	0.095	0.067	0.247	0.025	0	0.037	0	0	0	0.150	0.087	1.054
TCGA-BH-A0H5-01	0.079	0	0.035	0.094	0	0.436	0.013	0	0.013	0	0.076	0	0.038	0.074	0.093	0.039	0	0	0.01	0	0	0	0.010	0.402	0.919
TCGA-BH-A0H5-11	0.04	0	0.069	0.042	0	0.425	0	0	0	0	0.037	0.025	0.022	0.022	0.023	0.24	0	0.031	0.024	0	0	0	0.310	0.052	1.071
TCGA-BH-A0H6-01	0.081	0	0.044	0	0	0.219	0	0	0.019	0	0.013	0.013	0.026	0.077	0.039	0.379	0.002	0	0.085	0	0	0.003	0.430	0.029	1.077
TCGA-BH-A0H7-01	0.108	0	0.022	0.029	0	0.214	0	0.011	0.019	0	0.03	0	0	0.257	0.071	0.184	0	0	0.054	0	0	0	0.050	0.142	1.038
TCGA-BH-A0H7-11	0.154	0	0.103	0.071	0	0.262	0	0.005	0	0	0.026	0.007	0.02	0	0.035	0.266	0	0.015	0.036	0	0	0	0.340	0.046	1.058
TCGA-BH-A0H9-01	0.1	0	0.066	0	0	0.282	0	0	0	0	0	0.005	0.007	0.182	0.045	0.282	0	0	0.033	0	0	0	0.640	0.010	1.090
TCGA-BH-A0HA-01	0.06	0	0.068	0.024	0	0.246	0	0	0.065	0	0	0.037	0.018	0.064	0.138	0.083	0.008	0	0.189	0	0	0	0.040	0.153	1.026
TCGA-BH-A0HA-11	0.075	0	0.057	0.179	0	0.474	0	0	0	0	0.021	0.016	0.052	0.034	0	0	0	0.029	0.065	0	0	0	0.090	0.101	1.079
TCGA-BH-A0HB-01	0.047	0	0.055	0	0	0.344	0	0.022	0.02	0	0	0.041	0.035	0.143	0.06	0.187	0.005	0	0.041	0	0	0	0.020	0.246	0.985
TCGA-BH-A0HF-01	0.07	0	0.008	0.038	0	0.133	0	0.007	0.017	0	0.016	0	0	0.436	0.049	0.184	0.001	0	0.042	0	0	0	0.010	0.358	0.957
TCGA-BH-A0HI-01																									

	0.131	0	0.004	0.003	0	0.12	0	0.05	0.036	0	0	0.01	0	0.256	0.039	0.327	0	0	0.023	0	0	0.002	0.220	0.070	1.087
TCGA-BH-A0HK-01	0.101	0	0.237	0	0	0.319	0	0	0	0	0.027	0.002	0.025	0	0.049	0.206	0.001	0.004	0.029	0	0	0	0.440	0.026	1.057
TCGA-BH-A0HK-11	0.001	0	0.039	0	0	0.491	0	0.019	0.003	0	0.036	0.044	0.103	0	0.001	0.197	0.003	0.023	0.028	0	0.011	0	0.350	0.044	1.082
TCGA-BH-A0HL-01	0.11	0	0.294	0	0	0.225	0	0.005	0.031	0	0.011	0.033	0.007	0.136	0	0.129	0	0.005	0.013	0	0	0	0.720	0.000	1.068
TCGA-BH-A0HN-01	0	0.005	0	0	0	0.145	0	0.011	0.036	0	0.071	0	0.177	0	0.023	0.508	0.015	0	0	0.01	0	0	0.720	-0.003	1.123
TCGA-BH-A0HO-01	0.004	0	0.048	0	0	0.274	0	0.018	0.017	0	0.051	0	0	0.388	0.02	0.1	0	0	0.08	0	0	0	0.120	0.095	1.078
TCGA-BH-A0HP-01	0.139	0	0.047	0.027	0	0.236	0	0.014	0.034	0	0.035	0	0.044	0	0.056	0.321	0	0	0.046	0	0	0	0.310	0.052	1.059
TCGA-BH-A0HQ-01	0.048	0	0.048	0	0	0.07	0	0.005	0.028	0	0	0.011	0	0.466	0.012	0.13	0	0	0.182	0	0	0	0.010	0.276	1.007
TCGA-BH-A0HU-01	0.092	0	0.038	0.113	0	0.221	0	0.02	0.068	0	0.042	0	0.017	0.12	0.061	0.162	0	0.024	0.023	0	0	0	0.430	0.029	1.076
TCGA-BH-A0HW-01	0.127	0	0.18	0.006	0	0.051	0	0.004	0.057	0	0	0.026	0	0.2	0	0.236	0	0.073	0.042	0	0	0	0.890	-0.022	1.091
TCGA-BH-A0HX-01	0.119	0	0.007	0	0	0.303	0	0	0.017	0	0.029	0	0.001	0.288	0.052	0.16	0	0	0.025	0	0	0	0.350	0.044	1.089
TCGA-BH-A0HY-01	0.116	0	0.038	0	0	0.26	0	0.045	0.008	0	0.026	0.004	0	0.105	0.106	0.234	0	0	0.059	0	0	0	0.950	-0.027	1.096
TCGA-BH-A0RX-01	0.093	0	0.029	0.087	0	0.222	0.078	0.018	0.02	0	0.016	0.025	0.013	0.135	0.116	0.078	0	0.014	0.056	0	0	0	0.040	0.186	1.012
TCGA-BH-A0W3-01	0.056	0	0.059	0.001	0	0.158	0	0.01	0.006	0	0.011	0.01	0.207	0	0.04	0.381	0	0	0.06	0	0	0	0.740	-0.004	1.095
TCGA-BH-A0W4-01	0.027	0	0.045	0	0	0.273	0	0.028	0.034	0	0.013	0.034	0	0.371	0	0.107	0	0	0.069	0	0	0	0.090	0.100	1.074
TCGA-BH-A0W5-01	0.208	0.025	0.009	0.058	0	0.37	0.009	0.009	0	0	0.024	0	0.009	0.131	0.028	0.094	0	0.002	0.024	0	0	0	0.010	0.416	0.910
TCGA-BH-A0W7-01	0.061	0	0.039	0.025	0.016	0.216	0	0	0.003	0	0	0.001	0.02	0.271	0.131	0.121	0.008	0	0.089	0	0	0	0.010	0.366	0.936
TCGA-BH-A0WA-01	0.016	0	0	0	0	0.232	0	0.054	0.021	0.044	0	0	0	0.461	0.116	0.039	0	0	0.017	0	0	0	0.010	0.360	0.958
TCGA-BH-A18F-01	0.132	0	0.048	0.041	0	0.325	0.014	0.001	0.019	0	0.016	0	0.004	0.096	0.081	0.133	0.025	0	0.066	0	0	0	0.060	0.132	1.029
TCGA-BH-A18G-01	0.005	0	0	0.048	0	0.329	0.005	0	0	0	0.076	0	0.11	0	0.048	0.333	0	0.035	0.01	0	0	0	0.440	0.027	1.087
TCGA-BH-A18H-01	0.141	0	0.067	0.032	0	0.317	0.028	0	0.006	0	0.067	0	0.014	0.242	0.07	0.016	0	0	0	0.001	0	0	0.010	0.324	0.954
TCGA-BH-A18I-01	0.087	0	0.016	0.025	0	0.354	0.005	0.019	0.009	0	0.049	0	0.01	0.053	0.1	0.153	0.017	0.041	0.061	0	0	0	0.040	0.174	1.018
TCGA-BH-A18J-01	0.015	0	0	0	0	0.077	0	0.02	0.047	0	0	0.027	0	0.402	0.035	0.357	0	0	0.018	0	0	0	0.010	0.313	0.999
TCGA-BH-A18J-11	0.079	0	0.038	0.126	0	0.287	0	0	0	0	0.018	0	0.094	0.183	0.024	0.108	0	0	0.042	0	0	0	0.440	0.026	1.079
TCGA-BH-A18K-01	0.098	0	0.032	0	0	0.079	0	0.063	0.022	0	0	0.004	0	0.328	0.048	0.272	0.007	0	0.047	0	0	0	0.030	0.215	1.023
TCGA-BH-A18K-11	0.118	0	0.091	0.037	0	0.294	0	0	0	0	0.056	0	0.027	0	0.003	0.317	0	0.01	0.047	0	0	0	0.630	0.012	1.073
TCGA-BH-A18L-01	0	0	0.017	0	0	0.468	0	0.05	0	0	0	0	0.066	0	0.039	0.234	0	0	0.126	0	0	0	0.960	-0.030	1.106
TCGA-BH-A18L-11	0.038	0	0.024	0	0	0.335	0	0.002	0.046	0	0.029	0.064	0.078	0.053	0.008	0.27	0	0.006	0.047	0	0	0	0.610	0.013	1.085
TCGA-BH-A18M-01	0.072	0	0.047	0.11	0	0.211	0	0.004	0	0	0	0	0.082	0.019	0.014	0.198	0	0.043	0.2	0	0	0	0.410	0.033	1.063
TCGA-BH-A18M-11	0.112	0	0.066	0.095	0	0.246	0	0.01	0.004	0	0.02	0.003	0.131	0.027	0.021	0.115	0	0.021	0.129	0	0	0.001	0.450	0.025	1.062
TCGA-BH-A18N-01	0.083	0	0.047	0	0	0.089	0	0	0.016	0	0.005	0.007	0	0.311	0.024	0.378	0	0	0.04	0	0	0.001	0.310	0.057	1.116
TCGA-BH-A18N-11	0.069	0	0.104	0.004	0	0.224	0	0	0	0	0.027	0.029	0.07	0	0.017	0.326	0	0.004	0.127	0	0	0	0.410	0.033	1.060
TCGA-BH-A18P-01	0.134	0	0.062	0.005	0	0.233	0	0.021	0.032	0	0.023	0.003	0.004	0.214	0.085	0.163	0	0.008	0.014	0	0	0	0.070	0.118	1.043
TCGA-BH-A18P-11	0.069	0	0.044	0.004	0	0.454	0	0	0	0	0.045	0	0.041	0.023	0.004	0.23	0	0.015	0.065	0	0.007	0	0.760	-0.006	1.090
TCGA-BH-A18Q-01	0.086	0	0.015	0.103	0	0.182	0	0.073	0.029	0	0.049	0.011	0.032	0	0.149	0.174	0	0.057	0.04	0	0	0	0.090	0.100	1.061
TCGA-BH-A18Q-11	0.081	0	0.145	0	0	0.418	0	0	0	0	0.051	0.024	0.025	0.018	0.014	0.201	0	0.001	0.021	0	0	0	0.430	0.027	1.071
TCGA-BH-A18R-01	0.116	0	0.212	0.031	0	0.257	0.027	0.005	0	0	0.033	0	0.021	0.018	0.06	0.148	0.035	0.009	0.026	0	0	0	0.120	0.096	1.030
TCGA-BH-A18R-11	0.076	0	0.161	0.022	0	0.402	0	0	0	0	0.056	0	0.026	0	0	0.155	0	0.015	0.081	0.005	0	0	0.410	0.035	1.063
TCGA-BH-A18S-01	0.002	0	0.013	0	0	0.383	0	0.007	0.055	0	0.093	0.007	0.048	0.043	0.001	0.147	0	0.002	0.189	0	0.012	0	0.510	0.020	1.085
TCGA-BH-A18S-11	0	0	0.016	0.018	0	0.276	0	0.038	0	0	0	0.017	0	0.253	0.012	0.313	0	0	0.056	0	0.001	0	0.060	0.129	1.060
TCGA-BH-A18T-01	0.066	0	0.005	0.016	0	0.257	0	0.069	0.031	0	0.011	0.015	0	0.314	0.083	0.112	0	0	0.021	0	0	0	0.060	0.130	1.055
TCGA-BH-A18U-01	0.049	0	0.1	0	0	0.224	0	0.062	0.002	0	0	0.023	0.002	0.046	0.152	0.254	0.01	0	0.076	0	0	0.001	0.050	0.143	1.031
TCGA-BH-A18U-11	0.09	0	0.02	0.017	0	0.34	0	0.029	0	0	0.041	0.014	0.058	0	0.028	0.23	0	0.049	0.083	0	0	0	0.610	0.013	1.074
TCGA-BH-A18V-01	0.002	0	0	0	0	0.233	0	0.031	0.006	0	0.034	0.008	0.008	0.428	0.097	0.142	0	0	0.011	0	0	0	0.010	0.306	0.989
TCGA-BH-A18V-06	0.084	0	0.005	0.169	0	0.073	0.017	0.06	0.123	0	0.023	0.012	0.024	0.062	0.156	0.15	0	0.009	0.032	0	0	0	0.010	0.319	0.959
TCGA-BH-A1EN-01	0.1	0	0.143	0.052	0	0.14	0	0	0	0	0	0.056	0.021	0	0.01	0.381	0	0	0.096	0	0	0	0.960	-0.029	1.087
TCGA-BH-A1EN-11	0.155	0	0.042	0.088	0	0.239	0	0	0	0	0	0.042	0.094	0.001	0.02	0.201	0	0.005	0.108	0	0.005	0	0.440	0.026	1.066
TCGA-BH-A1EO-01	0.032	0	0	0	0	0.295	0	0.009	0	0	0.019	0	0.011	0.193	0.085	0.19	0.014	0	0.152	0	0	0	0.070	0.112	1.048
TCGA-BH-A1EO-11	0.005	0	0.004	0	0	0.181	0	0	0	0	0	0.044	0.056	0	0	0.589	0	0.009	0.11	0	0	0	0.030	0.218	1.021
TCGA-BH-A1ES-01	0.094	0	0.018	0	0	0.256	0	0	0	0	0.023	0.002	0	0.193	0	0.31	0	0.069	0.03	0	0	0.006	0.770	-0.011	1.107
TCGA-BH-A1ES-06	0.087	0	0.039	0.06	0	0.292	0	0	0.015	0	0.031	0.017	0.037	0.044	0.003	0.3	0	0.042	0	0	0	0.032	1.000	-0.056	1.110
TCGA-BH-A1ET-01	0.153	0	0.108	0.082	0	0.285	0	0.013	0	0	0	0.008	0.043	0	0.045	0.187	0.005	0.003	0.069	0	0	0	0.910	-0.023	1.082
TCGA-BH-A1ET-11	0.096	0	0.071	0	0	0.438	0	0	0	0	0	0	0.075	0	0.003	0.185	0	0.013	0.118	0	0	0	0.410	0.033	1.068
TCGA-BH-A1EU-01	0.108	0	0.001	0.045	0	0.409	0.001	0.004	0	0	0.003	0	0.013	0.094	0.057	0.199	0.007	0.016	0.043	0	0	0	0.040	0.192	1.013
TCGA-BH-A1EU-11	0.203	0	0.02	0.032	0	0.241	0	0	0	0	0.01	0.019	0.024	0.006	0	0.301	0.008	0.023	0.113	0	0	0	0.430	0.029	1.069
TCGA-BH-A1EV-01	0	0.003	0.025	0	0	0.541	0	0.003	0.02	0	0.064	0.021	0.051	0.186	0.005	0.055	0	0	0.027	0	0	0	0.410	0.031	1.104
TCGA-BH-A1EV-11	0.12	0	0.026	0.044	0	0.277	0	0.005	0	0	0	0.076	0.039	0	0.034	0.253	0	0	0	0.126	0	0	0.410	0.037	1.080

TCGA-BH-A1EW-01	0.064	0	0.034	0	0	0.392	0.055	0	0	0	0.06	0	0.075	0	0.076	0.148	0	0.021	0.076	0	0	0	0.070	0.123	1.033
TCGA-BH-A1EW-11	0.122	0	0.155	0.147	0	0.235	0	0	0	0	0	0.028	0.059	0.053	0.012	0.041	0	0.028	0.12	0	0.001	0	0.600	0.016	1.067
TCGA-BH-A1EX-01	0.062	0	0	0	0	0.28	0	0.034	0.014	0	0.019	0	0	0.249	0.028	0.29	0	0.002	0.021	0	0	0	0.220	0.071	1.083
TCGA-BH-A1EY-01	0.053	0	0	0.024	0	0.377	0.002	0	0	0	0.039	0	0.021	0.131	0.066	0.193	0.033	0.001	0.059	0	0	0	0.030	0.220	0.999
TCGA-BH-A1F0-01	0.051	0	0.074	0	0	0.253	0	0.045	0	0	0.02	0.012	0.057	0.044	0.139	0.203	0	0.009	0.088	0	0.004	0	0.010	0.351	0.938
TCGA-BH-A1F0-11	0.114	0	0.105	0.075	0	0.266	0	0.068	0	0	0	0.024	0.046	0	0.045	0.191	0	0.021	0.043	0	0.001	0	0.410	0.037	1.061
TCGA-BH-A1F2-01	0.009	0	0.002	0	0	0.267	0	0.019	0.051	0	0.033	0.018	0.029	0.404	0.009	0.063	0	0	0.098	0	0	0	0.150	0.089	1.085
TCGA-BH-A1F2-11	0.091	0	0.075	0	0	0.299	0	0	0	0	0.022	0.047	0.059	0	0.042	0.273	0	0.003	0.089	0	0	0	0.730	-0.003	1.075
TCGA-BH-A1F5-01	0.03	0	0.045	0	0	0.326	0	0.073	0	0	0	0.038	0.014	0	0.022	0.216	0.055	0.005	0.178	0	0	0	0.740	-0.005	1.081
TCGA-BH-A1F6-01	0.116	0	0.064	0	0	0.111	0	0.042	0.001	0.017	0	0.015	0	0.294	0.098	0.228	0	0.008	0	0.005	0	0	0.010	0.286	0.981
TCGA-BH-A1F6-11	0.036	0	0.009	0	0	0.254	0	0.012	0	0	0	0.104	0.04	0	0.003	0.359	0	0.013	0.168	0	0	0	0.300	0.057	1.070
TCGA-BH-A1F8-01	0.093	0	0.136	0.014	0	0.248	0	0.024	0	0	0	0.008	0	0.053	0.038	0.225	0	0.107	0.042	0	0	0.013	0.720	-0.003	1.072
TCGA-BH-A1F8-11	0.006	0	0.061	0	0	0.362	0	0.003	0.016	0	0	0.03	0.057	0	0.012	0.233	0	0.027	0.194	0	0	0	0.550	0.019	1.073
TCGA-BH-A1FB-01	0.162	0	0	0	0	0.345	0.004	0.01	0.002	0	0.044	0	0.042	0.062	0.105	0.066	0.01	0	0.146	0	0	0	0.030	0.201	1.005
TCGA-BH-A1FB-11	0.012	0	0.133	0.185	0	0.254	0	0.015	0	0	0	0.007	0.102	0.014	0.021	0.117	0	0.004	0.131	0	0.005	0	0.370	0.040	1.063
TCGA-BH-A1FC-01	0.066	0	0.28	0.006	0	0.209	0.056	0.031	0.018	0	0	0.045	0.008	0.052	0.097	0.064	0	0.006	0.059	0	0.003	0	0.010	0.286	0.962
TCGA-BH-A1FC-11	0.026	0	0.023	0.099	0	0.289	0	0.022	0	0	0	0.019	0.123	0.038	0.095	0.148	0.001	0	0.098	0	0.02	0	0.210	0.075	1.055
TCGA-BH-A1FD-11	0.065	0	0.044	0.031	0	0.313	0	0	0	0	0.011	0.024	0.043	0	0.02	0.383	0	0.007	0.013	0.047	0	0	0.430	0.029	1.079
TCGA-BH-A1FE-01	0.191	0	0	0	0	0.351	0	0.006	0	0	0.042	0.005	0.033	0.032	0.149	0.061	0.002	0	0.129	0	0	0	0.010	0.316	0.959
TCGA-BH-A1FE-06	0.033	0	0.004	0	0	0.423	0	0	0	0	0.074	0.028	0.083	0	0.001	0.335	0	0.011	0	0.007	0	0	0.700	0.003	1.103
TCGA-BH-A1FE-11	0.066	0	0.04	0.077	0	0.537	0	0	0	0	0.007	0.005	0.019	0.028	0	0.176	0	0.041	0	0.004	0	0	0.350	0.044	1.095
TCGA-BH-A1FG-01	0.051	0	0.021	0.034	0	0.211	0	0.021	0	0	0	0.003	0.062	0.003	0.067	0.288	0.004	0	0.235	0	0	0	0.700	0.002	1.085
TCGA-BH-A1FG-11	0.165	0	0.02	0.123	0	0.253	0	0.029	0	0	0	0.031	0.02	0	0.023	0.231	0	0.006	0.064	0.034	0	0	0.660	0.008	1.084
TCGA-BH-A1FH-01	0.105	0	0.097	0.006	0	0.285	0	0	0	0	0.016	0.012	0.025	0	0.029	0.345	0	0.003	0.079	0	0	0	0.480	0.023	1.067
TCGA-BH-A1FH-11	0.011	0	0.022	0.092	0	0.242	0	0	0	0	0	0.06	0.098	0.005	0.009	0.285	0.006	0	0.138	0	0	0.032	0.340	0.047	1.068
TCGA-BH-A1FJ-01	0.106	0	0.031	0	0	0.137	0	0.032	0.038	0	0.01	0.008	0	0.12	0	0.305	0	0.148	0.061	0	0	0.003	0.910	-0.025	1.099
TCGA-BH-A1FJ-11	0.097	0	0.022	0	0	0.253	0	0.028	0	0.025	0.013	0	0.008	0	0.029	0.432	0	0.017	0.075	0	0	0	0.320	0.049	1.070
TCGA-BH-A1FL-01	0.176	0	0.048	0.007	0	0.192	0	0.049	0.018	0	0.054	0	0.028	0.067	0.044	0.212	0	0	0.105	0	0	0	0.690	0.004	1.070
TCGA-BH-A1FM-01	0.001	0.004	0.008	0	0.018	0.253	0	0	0.037	0	0.05	0	0	0.352	0.002	0.136	0	0	0.111	0	0	0.028	0.210	0.077	1.086
TCGA-BH-A1FM-11	0.11	0	0.11	0.113	0	0.218	0	0	0	0	0.024	0.023	0.049	0	0.002	0.274	0	0.009	0.067	0	0.002	0	0.660	0.007	1.071
TCGA-BH-A1FN-01	0.089	0	0.007	0	0	0.074	0	0.067	0.022	0.011	0	0.009	0	0.532	0.068	0.119	0	0	0.003	0	0	0	0.020	0.225	1.053
TCGA-BH-A1FN-11	0.035	0	0.072	0.068	0	0.248	0	0.038	0	0	0	0.008	0.062	0	0.047	0.277	0.002	0	0.144	0	0	0	0.050	0.140	1.022
TCGA-BH-A1FR-01	0.105	0	0.021	0.031	0	0.191	0	0.011	0.035	0	0.026	0	0	0.238	0.063	0.276	0	0	0.004	0	0	0	0.070	0.122	1.055
TCGA-BH-A1FR-11	0.113	0	0.055	0.015	0	0.118	0	0.007	0.013	0	0.01	0	0.004	0.277	0.034	0.287	0.008	0	0.06	0	0	0	0.010	0.282	0.981
TCGA-BH-A1FU-01	0.091	0	0	0.03	0	0.329	0.016	0.005	0	0	0.04	0	0.006	0.259	0.063	0.118	0	0.014	0.028	0	0	0	0.010	0.271	0.982
TCGA-BH-A1FU-11	0.04	0	0.055	0.147	0	0.305	0	0.006	0	0	0.013	0.016	0.082	0.126	0.026	0.014	0	0	0.172	0	0	0	0.410	0.033	1.076
TCGA-BH-A201-01	0	0	0.01	0	0	0.376	0	0.083	0.007	0	0.033	0.014	0	0.406	0.008	0	0	0.006	0.058	0	0	0	0.050	0.136	1.068
TCGA-BH-A202-01	0.056	0	0	0.011	0	0.233	0	0.087	0.017	0	0.006	0.022	0	0.144	0.091	0.263	0	0	0.07	0	0	0	0.040	0.147	1.033
TCGA-BH-A203-01	0.075	0	0.072	0.016	0	0.288	0	0.03	0	0	0.026	0	0.026	0.093	0.074	0.259	0	0.02	0.022	0	0	0	0.040	0.190	1.007
TCGA-BH-A203-11	0.102	0	0.102	0	0	0.37	0	0	0	0	0.063	0	0.04	0	0	0.208	0	0.066	0.048	0	0.001	0	0.660	0.007	1.071
TCGA-BH-A204-01	0	0	0	0.098	0	0.248	0	0.001	0	0	0.008	0.028	0.038	0	0.002	0.476	0	0	0.09	0	0	0.01	0.990	-0.041	1.125
TCGA-BH-A204-11	0.107	0	0.031	0	0	0.415	0	0.003	0	0	0.018	0.082	0.083	0	0.007	0.194	0	0.007	0.052	0	0.003	0	0.710	0.002	1.092
TCGA-BH-A208-01	0.138	0	0.096	0.057	0	0.138	0	0.016	0.027	0	0.003	0	0.006	0.002	0.002	0.326	0	0.142	0.048	0	0	0	0.220	0.069	1.050
TCGA-BH-A208-11	0.092	0	0.257	0.011	0	0.297	0	0.016	0	0	0.038	0	0.025	0.014	0	0.182	0	0.058	0.011	0	0	0	0.350	0.045	1.050
TCGA-BH-A209-01	0.117	0	0.04	0.077	0	0.366	0.02	0	0	0	0.037	0	0.018	0.063	0.108	0.094	0	0.021	0.04	0	0	0	0.000	0.450	0.892
TCGA-BH-A209-11	0.078	0	0.042	0.043	0	0.354	0	0	0	0	0.031	0.006	0.043	0	0.073	0.239	0	0.044	0.046	0	0	0	0.090	0.100	1.045
TCGA-BH-A28O-01	0.075	0	0.109	0.019	0	0.454	0	0	0	0	0.033	0.01	0.018	0	0.038	0.186	0	0.026	0.031	0	0	0	0.130	0.094	1.049
TCGA-BH-A28Q-01	0.082	0	0.02	0.015	0	0.429	0	0	0	0	0.028	0	0.064	0	0.06	0.237	0.008	0.025	0.033	0	0	0	0.590	0.017	1.083
TCGA-BH-A2L8-01	0.099	0	0.019	0.156	0	0.258	0.026	0.042	0.025	0	0.008	0.001	0.026	0.024	0.083	0.189	0	0	0.044	0	0	0	0.010	0.295	0.966
TCGA-BH-A42T-01	0.081	0	0.006	0.039	0	0.171	0	0.092	0.013	0	0	0.01	0.081	0	0.019	0.288	0	0.033	0	0.166	0	0	0.090	0.103	1.049
TCGA-BH-A42U-01	0.111	0.004	0.054	0.081	0	0.271	0	0.016	0.051	0	0.015	0.011	0.013	0.146	0.043	0.161	0	0	0	0.024	0	0	0.040	0.154	1.025
TCGA-BH-A42V-01	0.13	0	0.016	0.078	0	0.3	0	0.016	0.018	0	0.025	0	0.04	0.065	0.041	0.176	0.036	0	0.06	0	0	0	0.050	0.142	1.026
TCGA-BH-A5IZ-01	0.035	0	0.049	0.042	0	0.082	0	0	0.016	0	0.016	0.007	0.012	0.372	0.018	0.232	0	0.106	0	0	0	0.013	0.070	0.108	1.090
TCGA-BH-A5J0-01	0.055	0	0.016	0.033	0	0.176	0	0.031	0.063	0	0.01	0.003	0.017	0.205	0.085	0.253	0.006	0	0.048	0	0	0	0.010	0.390	0.923
TCGA-BH-A6R8-01	0.1	0	0.045	0	0	0.157	0	0.05	0.076	0	0.026	0.013	0.025	0.185	0.056	0.229	0	0	0.037	0	0	0	0.030	0.214	0.999
TCGA-BH-A6R9-01	0.005	0	0.006	0.029	0	0.143	0	0	0.018	0	0.042	0	0	0.243	0.073	0.327	0.017	0	0.099	0	0	0	0.000	0.459	0.889
TCGA-BH-A8FY-01	0	0.039	0	0	0	0.33	0	0.105	0	0	0.052	0.029	0.075	0	0.014	0.214	0.012	0	0.13	0	0.001	0	0.820	-0.015	1.092

TCGA-BH-A8FZ-01	0.093	0	0.008	0	0	0.238	0.022	0.029	0.013	0	0.038	0	0.039	0.159	0.022	0.153	0.038	0.049	0.098	0	0	0	0.040	0.191	1.003
TCGA-BH-A8G0-01	0.099	0.031	0	0.006	0	0.148	0	0	0.011	0	0.035	0	0	0.462	0.013	0.151	0	0	0.045	0	0	0	0.000	0.722	0.708
TCGA-BH-AB28-01	0.196	0	0	0.062	0	0.3	0.012	0	0.009	0	0.048	0	0.034	0	0.067	0.238	0.003	0.01	0.021	0	0	0	0.050	0.138	1.033
TCGA-C8-A12K-01	0.031	0	0.011	0.084	0	0.16	0	0.031	0.067	0	0.027	0.035	0.013	0.151	0.232	0.13	0	0	0.027	0	0	0	0.010	0.389	0.934
TCGA-C8-A12L-01	0.051	0	0.015	0	0	0.319	0.008	0.073	0.002	0	0.038	0.015	0.017	0.177	0.089	0.116	0.017	0	0.062	0	0	0	0.210	0.076	1.060
TCGA-C8-A12M-01	0.112	0	0.104	0.016	0	0.31	0	0.027	0.041	0	0.037	0	0.021	0.054	0.049	0.119	0	0.005	0.106	0	0	0	0.890	-0.019	1.079
TCGA-C8-A12N-01	0.078	0	0	0.015	0	0.164	0	0.018	0.016	0	0	0.033	0.018	0	0.057	0.275	0.179	0	0.138	0	0	0.011	0.070	0.115	1.036
TCGA-C8-A12O-01	0.068	0	0.034	0.003	0	0.168	0	0.025	0.053	0	0.008	0	0	0.352	0.053	0.175	0	0	0.061	0	0	0	0.010	0.344	0.951
TCGA-C8-A12P-01	0.071	0	0.067	0	0	0.217	0	0.009	0.027	0	0.013	0.002	0	0.151	0.093	0.274	0	0.017	0.06	0	0	0	0.030	0.208	1.003
TCGA-C8-A12Q-01	0.064	0	0	0	0	0.146	0	0.044	0.025	0	0	0.013	0	0.388	0.07	0.18	0	0	0.061	0	0	0	0.020	0.242	1.015
TCGA-C8-A12T-01	0.019	0	0.083	0.032	0	0.241	0	0.023	0.005	0	0.005	0.044	0.018	0.055	0.047	0.245	0	0.04	0.143	0	0	0	0.220	0.072	1.048
TCGA-C8-A12U-01	0.032	0	0.015	0.209	0	0.166	0.006	0.034	0.021	0	0	0.052	0.034	0.088	0.108	0.096	0.002	0	0.135	0	0.001	0	0.040	0.173	1.024
TCGA-C8-A12V-01	0.076	0	0.028	0.088	0	0.226	0.015	0.049	0.063	0	0.06	0	0.01	0.134	0.148	0.088	0.002	0.004	0.008	0	0	0	0.000	0.516	0.857
TCGA-C8-A12W-01	0.065	0	0.162	0.01	0	0.106	0	0.002	0.038	0	0.022	0	0	0.294	0.04	0.235	0	0	0.028	0	0	0	0.010	0.313	0.963
TCGA-C8-A12X-01	0.084	0	0.072	0	0	0.091	0	0.007	0.06	0	0.017	0.023	0	0.24	0.013	0.322	0	0	0.058	0	0	0.013	0.040	0.155	1.038
TCGA-C8-A12Y-01	0.018	0	0.068	0.034	0	0.149	0	0.044	0.057	0	0	0.064	0.017	0.047	0.095	0.265	0	0.001	0.14	0	0	0	0.340	0.047	1.063
TCGA-C8-A12Z-01	0.042	0	0.023	0	0	0.279	0	0.052	0.062	0	0.046	0	0	0.408	0.016	0	0	0	0.07	0	0	0.001	0.070	0.108	1.073
TCGA-C8-A130-01	0.092	0	0.024	0.077	0	0.278	0	0.027	0.038	0	0.045	0	0.005	0.052	0.082	0.208	0	0	0.075	0	0	0	0.260	0.063	1.061
TCGA-C8-A131-01	0.051	0	0.036	0.012	0	0.226	0	0.028	0	0	0.015	0.012	0	0.42	0.046	0.143	0	0	0.011	0	0	0	0.050	0.145	1.067
TCGA-C8-A132-01	0.19	0	0.024	0.011	0	0.281	0.005	0	0.044	0	0.05	0	0.041	0.222	0.051	0.068	0	0.011	0.001	0	0	0	0.020	0.226	0.997
TCGA-C8-A133-01	0.1	0	0.097	0.017	0	0.051	0	0.008	0	0	0	0.029	0.005	0	0.014	0.537	0	0.018	0.121	0	0	0.001	0.440	0.026	1.093
TCGA-C8-A134-01	0.006	0.012	0	0	0	0.139	0	0.012	0.007	0.038	0.012	0	0.011	0.444	0.211	0.082	0	0	0.006	0	0.018	0	0.000	0.572	0.823
TCGA-C8-A135-01	0.129	0	0.069	0.047	0	0.335	0	0.021	0.033	0	0.015	0	0.037	0	0.058	0.224	0.008	0	0.023	0	0	0	0.190	0.082	1.051
TCGA-C8-A137-01	0.09	0	0.131	0	0	0.33	0	0.027	0.056	0	0	0.056	0.016	0.212	0.031	0	0	0	0.044	0	0.006	0	0.760	-0.009	1.089
TCGA-C8-A138-01	0.047	0	0.037	0.04	0	0.149	0	0.041	0.056	0.002	0	0.047	0.002	0.273	0.07	0.123	0	0.002	0.105	0	0.007	0	0.010	0.374	0.930
TCGA-C8-A1HE-01	0.081	0	0.026	0.009	0	0.322	0	0	0	0	0.03	0	0.068	0	0.058	0.354	0.027	0	0.021	0	0	0.006	0.840	-0.017	1.095
TCGA-C8-A1HF-01	0.009	0	0.066	0.063	0	0.356	0.032	0.04	0.03	0	0	0.027	0.014	0.033	0.13	0.127	0.006	0	0.069	0	0	0	0.010	0.250	0.989
TCGA-C8-A1HG-01	0.01	0	0.154	0.059	0	0.286	0.019	0.003	0.027	0	0	0.03	0.008	0.065	0.105	0.179	0	0	0.056	0	0	0	0.010	0.252	0.980
TCGA-C8-A1HI-01	0.096	0	0.175	0	0	0.16	0	0.004	0	0.013	0	0.034	0.004	0.036	0.059	0.298	0	0.002	0.12	0	0	0	0.410	0.034	1.057
TCGA-C8-A1HJ-01	0.082	0	0.025	0.022	0	0.304	0	0.066	0	0	0.045	0	0.024	0.091	0.128	0.078	0	0.092	0.035	0	0.006	0	0.130	0.092	1.062
TCGA-C8-A1HK-01	0.085	0	0.108	0.019	0	0.278	0	0.003	0.056	0	0	0.032	0.007	0	0.076	0.25	0	0.022	0.063	0	0	0	0.710	0.002	1.075
TCGA-C8-A1HL-01	0.113	0	0.055	0	0	0.08	0	0.001	0.025	0	0.006	0	0	0.369	0.017	0.286	0	0	0.047	0	0	0	0.040	0.190	1.046
TCGA-C8-A1HM-01	0.073	0	0.025	0.146	0	0.141	0.081	0.085	0.021	0	0	0.064	0.008	0.018	0.125	0.11	0	0.003	0.1	0	0	0	0.010	0.314	0.959
TCGA-C8-A1HN-01	0.003	0	0.016	0	0	0.224	0	0.021	0.09	0	0.078	0	0.012	0.394	0.005	0.157	0	0	0	0	0	0	0.120	0.095	1.091
TCGA-C8-A1HO-01	0.07	0	0.231	0.012	0	0.225	0	0	0.017	0	0.013	0	0	0.14	0.092	0.164	0	0	0.035	0	0	0	0.610	0.014	1.068
TCGA-C8-A26V-01	0.015	0	0.02	0	0	0.235	0	0.036	0	0	0.028	0.012	0	0.199	0.139	0.205	0.004	0.063	0.045	0	0	0	0.070	0.112	1.064
TCGA-C8-A26W-01	0.108	0	0.031	0.08	0	0.344	0.003	0.025	0.028	0	0.037	0	0.034	0	0.078	0.223	0	0	0.009	0	0	0	0.110	0.096	1.055
TCGA-C8-A26X-01	0.052	0	0.125	0	0	0.155	0.007	0.039	0.011	0.007	0.01	0.018	0	0.292	0.094	0.144	0	0	0.048	0	0	0	0.010	0.419	0.908
TCGA-C8-A26Y-01	0.108	0	0	0.044	0	0.112	0	0.051	0.063	0	0.032	0	0	0.351	0.023	0.212	0	0.002	0	0	0	0	0.020	0.225	1.018
TCGA-C8-A26Z-01	0.111	0	0.122	0.049	0	0.138	0	0.006	0.013	0	0.01	0.033	0.025	0	0.051	0.331	0.015	0	0.096	0	0	0	1.000	-0.057	1.091
TCGA-C8-A273-01	0.072	0	0.087	0	0	0.186	0	0.025	0.035	0	0	0.012	0	0.28	0.026	0.244	0	0.017	0.01	0.005	0	0	0.130	0.091	1.067
TCGA-C8-A274-01	0.086	0	0.05	0	0	0.214	0	0.026	0.008	0	0.03	0	0.029	0	0.093	0.394	0.008	0	0.049	0	0	0.013	0.960	-0.030	1.100
TCGA-C8-A275-01	0.02	0	0.023	0	0	0.29	0	0.04	0.001	0	0	0.043	0.01	0.156	0.131	0.159	0.001	0.012	0.108	0	0.006	0	0.020	0.232	0.997
TCGA-C8-A278-01	0.064	0	0.205	0.001	0	0.209	0.007	0.002	0	0	0.011	0	0	0.136	0.081	0.202	0.009	0.009	0.064	0	0	0	0.010	0.311	0.953
TCGA-C8-A27A-01	0.09	0	0.066	0	0	0.191	0	0.021	0.039	0	0	0.006	0	0.221	0.107	0.255	0	0	0.004	0	0	0	0.070	0.119	1.054
TCGA-C8-A27B-01	0.009	0	0	0	0	0.205	0	0.007	0.004	0.025	0	0	0	0.41	0.202	0.076	0	0	0.055	0	0	0.007	0.010	0.437	0.915
TCGA-C8-A3M7-01	0.096	0	0.195	0	0	0.284	0	0	0	0	0.018	0.032	0.032	0	0.069	0.142	0	0	0.132	0	0	0	0.380	0.039	1.051
TCGA-C8-A3M8-01	0.121	0	0.007	0.013	0	0.101	0	0.013	0.086	0	0.021	0	0	0.344	0.047	0.227	0	0	0.018	0	0	0	0.600	0.015	1.119
TCGA-C8-A8HP-01	0.05	0	0.08	0.091	0	0.183	0	0.043	0.077	0	0.033	0.009	0.015	0.135	0.074	0.156	0	0.027	0.027	0	0	0	0.010	0.327	0.948
TCGA-C8-A8HQ-01	0.003	0	0.067	0	0	0.185	0	0.075	0.012	0	0	0.06	0.032	0.306	0.115	0.077	0.008	0	0.059	0	0	0	0.010	0.292	0.973
TCGA-C8-A8HR-01	0.046	0	0	0.02	0	0.39	0	0.041	0.007	0	0.029	0.013	0.027	0	0.131	0.296	0	0	0	0	0	0	0.030	0.214	1.012
TCGA-D8-A13Y-01	0	0	0	0	0	0.135	0	0.132	0	0	0	0.083	0.022	0.272	0.004	0.293	0	0	0.058	0	0	0	0.950	-0.029	1.135
TCGA-D8-A13Z-01	0.077	0	0	0	0	0.167	0	0.032	0.026	0	0.04	0	0	0.382	0.046	0.185	0	0.037	0.008	0	0	0	0.010	0.412	0.919
TCGA-D8-A140-01	0.095	0	0.183	0.005	0	0.245	0	0.019	0.046	0	0.016	0.021	0.021	0.062	0.061	0.153	0	0.015	0.058	0	0.001	0	0.310	0.056	1.045
TCGA-D8-A141-01	0.1	0	0.006	0.045	0	0.357	0.004	0.013	0.032	0	0.047	0	0.016	0.165	0.081	0.104	0	0	0.029	0	0	0	0.010	0.255	0.986
TCGA-D8-A142-01	0.051	0	0.159	0.004	0	0.192	0	0.019	0.02	0	0.016	0	0.007	0.266	0.061	0.157	0	0	0.048	0	0	0	0.040	0.176	1.017
TCGA-D8-A143-01																									

	0.118	0	0.119	0.002	0	0.28	0	0.054	0.004	0	0.032	0.01	0.001	0.201	0.052	0.113	0	0.004	0.01	0	0	0	0.130	0.090	1.047
TCGA-D8-A145-01	0.106	0	0.236	0.002	0	0.233	0	0.004	0.004	0	0.038	0	0.022	0.087	0.045	0.155	0	0	0.07	0	0	0	0.290	0.059	1.041
TCGA-D8-A146-01	0.12	0	0.002	0.01	0	0.173	0	0.016	0.004	0	0.043	0	0.001	0.308	0.042	0.138	0	0	0.143	0	0	0	0.090	0.100	1.060
TCGA-D8-A147-01	0.066	0	0.008	0.009	0	0.325	0	0.078	0	0	0.019	0.013	0.041	0.248	0.034	0.066	0	0.055	0.037	0	0	0	0.040	0.147	1.035
TCGA-D8-A1J8-01	0.064	0	0.007	0.033	0	0.337	0.025	0.064	0	0	0.016	0.011	0	0.107	0.152	0.142	0	0.025	0.012	0	0	0.006	0.010	0.301	0.970
TCGA-D8-A1J9-01	0.002	0	0.004	0	0	0.249	0	0.027	0.076	0	0.009	0.02	0.04	0.265	0.018	0.177	0.001	0	0.11	0	0	0	0.090	0.102	1.059
TCGA-D8-A1JA-01	0.049	0	0.002	0	0	0.253	0	0.029	0	0	0.02	0.003	0	0	0.021	0.239	0	0.282	0.018	0	0	0.086	0.410	0.035	1.096
TCGA-D8-A1JB-01	0.112	0	0.018	0.051	0	0.316	0	0.062	0.029	0	0.025	0	0.015	0.024	0.125	0.149	0.007	0	0.066	0	0	0	0.010	0.330	0.951
TCGA-D8-A1JC-01	0.106	0	0.028	0	0	0.21	0	0.033	0.045	0	0.029	0	0	0.315	0.066	0.122	0	0	0.048	0	0	0	0.040	0.189	1.021
TCGA-D8-A1JD-01	0.067	0	0.056	0	0	0.194	0.002	0.039	0.04	0	0.048	0	0.007	0.005	0.079	0.295	0.028	0	0.136	0	0	0.004	0.060	0.130	1.025
TCGA-D8-A1JE-01	0.062	0	0	0	0	0.418	0	0.003	0.002	0	0.03	0	0.032	0.023	0.077	0.224	0.008	0	0.12	0	0	0	0.720	-0.000	1.088
TCGA-D8-A1JF-01	0.11	0	0.119	0	0	0.214	0.019	0.009	0.008	0	0.051	0	0.006	0.13	0.034	0.232	0	0.016	0.052	0	0	0	0.310	0.055	1.052
TCGA-D8-A1JG-01	0.104	0	0.137	0	0	0.282	0.011	0	0	0	0.03	0	0.002	0.097	0.11	0.176	0.01	0	0.039	0	0	0	0.040	0.194	1.002
TCGA-D8-A1JH-01	0.059	0	0.019	0.048	0	0.252	0	0.004	0.009	0	0.024	0	0.037	0	0.031	0.344	0.045	0.029	0.099	0	0	0	0.060	0.131	1.030
TCGA-D8-A1JI-01	0.098	0	0.042	0.039	0	0.086	0	0.039	0	0	0	0.037	0.069	0	0.033	0.444	0.024	0	0.089	0	0	0	0.770	-0.011	1.096
TCGA-D8-A1JJ-01	0.042	0	0.077	0	0	0.092	0	0.019	0.026	0	0	0.008	0	0.435	0.07	0.213	0	0	0.018	0	0	0	0.010	0.262	1.016
TCGA-D8-A1JK-01	0.059	0	0.025	0.068	0	0.171	0.04	0.02	0.003	0	0.064	0	0.003	0.234	0.136	0.152	0	0	0.026	0	0	0	0.130	0.091	1.067
TCGA-D8-A1JL-01	0.015	0	0	0	0	0.229	0.003	0.039	0.034	0.002	0.018	0	0	0.334	0.134	0.124	0	0.014	0.055	0	0	0	0.010	0.392	0.929
TCGA-D8-A1JN-01	0.09	0	0.016	0.077	0	0.285	0	0.069	0.027	0	0.017	0	0.067	0	0.063	0.228	0	0	0.061	0	0	0	0.890	-0.021	1.093
TCGA-D8-A1JP-01	0.044	0	0	0.064	0	0.228	0	0.019	0.023	0	0.055	0	0.013	0.048	0.053	0.415	0	0	0.037	0	0	0	0.090	0.100	1.057
TCGA-D8-A1JS-01	0.002	0	0.04	0	0	0.278	0	0.068	0.026	0	0.065	0	0.019	0.315	0.007	0.099	0.015	0	0.067	0	0	0	0.260	0.062	1.080
TCGA-D8-A1JT-01	0.029	0	0.036	0	0	0.065	0	0.057	0.001	0.033	0	0.031	0	0.319	0.067	0.281	0	0.015	0.066	0	0	0	0.410	0.034	1.113
TCGA-D8-A1JU-01	0.06	0	0.006	0.056	0	0.221	0	0.001	0.004	0	0.038	0.005	0.023	0	0.04	0.361	0	0	0.186	0	0	0	0.120	0.096	1.049
TCGA-D8-A1X5-01	0.059	0	0.044	0.015	0	0.227	0	0.024	0.023	0	0	0.005	0.162	0.048	0.106	0.249	0	0	0.041	0	0	0	0.310	0.052	1.066
TCGA-D8-A1X6-01	0.002	0	0	0	0	0.332	0	0.048	0.063	0	0.033	0	0.044	0.019	0.018	0.431	0	0	0.011	0	0	0	0.180	0.085	1.070
TCGA-D8-A1X7-01	0.041	0	0.018	0	0	0.341	0	0.064	0.006	0	0.038	0.014	0.01	0.31	0	0.13	0	0	0.029	0	0	0	0.050	0.141	1.051
TCGA-D8-A1X8-01	0.084	0	0.065	0	0	0.318	0	0.004	0	0	0.014	0.007	0.068	0	0.058	0.331	0	0	0.049	0	0	0	0.130	0.091	1.045
TCGA-D8-A1X9-01	0.053	0	0.006	0.024	0	0.255	0	0.044	0.047	0	0.032	0	0.024	0.133	0.047	0.228	0	0.069	0.038	0	0	0	0.060	0.131	1.036
TCGA-D8-A1XA-01	0.045	0	0	0	0	0.109	0	0.024	0.073	0	0.012	0	0	0.359	0.043	0.19	0	0	0.145	0	0	0	0.350	0.043	1.105
TCGA-D8-A1XB-01	0.067	0	0.051	0	0	0.16	0	0.015	0.028	0	0.005	0.012	0	0.047	0.048	0.311	0.006	0	0.252	0	0	0	0.310	0.052	1.067
TCGA-D8-A1XC-01	0.046	0	0.054	0	0	0.232	0	0.03	0.036	0	0.05	0.038	0.017	0.09	0.03	0.286	0	0	0.089	0	0	0	0.540	0.020	1.073
TCGA-D8-A1XD-01	0.079	0	0.09	0	0	0.272	0	0	0.005	0	0	0.051	0.009	0.187	0.005	0.231	0	0	0.071	0	0	0	0.700	0.002	1.085
TCGA-D8-A1XF-01	0.084	0	0.183	0.007	0	0.054	0	0.023	0.043	0	0	0.036	0	0.241	0.063	0.207	0	0	0.058	0	0	0	0.310	0.055	1.066
TCGA-D8-A1XG-01	0.063	0	0	0	0	0.239	0	0	0.023	0	0.05	0.022	0.016	0	0.017	0.386	0.072	0.002	0.109	0	0	0	0.340	0.045	1.070
TCGA-D8-A1XJ-01	0.025	0	0.008	0	0	0.318	0	0.02	0.096	0	0.04	0	0.009	0.404	0.005	0.022	0	0	0.054	0	0	0	0.630	0.012	1.124
TCGA-D8-A1XK-01	0.03	0	0.015	0.029	0	0.223	0.034	0.01	0	0	0.071	0	0.013	0.384	0.066	0.125	0	0	0	0	0	0	0.040	0.163	1.051
TCGA-D8-A1XL-01	0.049	0	0.001	0	0	0.349	0.002	0.058	0.048	0	0.004	0.001	0.048	0.043	0.158	0.175	0.002	0	0.052	0	0.011	0	0.010	0.314	0.961
TCGA-D8-A1XM-01	0.02	0	0	0.049	0	0.257	0	0	0.03	0	0	0	0.053	0.097	0.124	0.185	0.041	0	0.142	0	0.003	0	0.030	0.210	1.003
TCGA-D8-A1XO-01	0.083	0	0.03	0.047	0	0.304	0	0.004	0	0	0.018	0	0.136	0	0.034	0.26	0.036	0.012	0.036	0	0	0	0.440	0.027	1.070
TCGA-D8-A1XQ-01	0.026	0	0.107	0.033	0	0.201	0.01	0.013	0.009	0	0.027	0	0.002	0.2	0.132	0.23	0	0	0.008	0	0	0	0.010	0.353	0.942
TCGA-D8-A1XR-01	0.133	0	0.002	0	0	0.135	0	0.01	0.06	0	0.011	0.016	0	0.35	0.012	0.258	0	0	0.012	0	0	0	0.420	0.030	1.119
TCGA-D8-A1XS-01	0.076	0	0.029	0	0	0.279	0	0.029	0.016	0	0.04	0	0.038	0.032	0.097	0.282	0.001	0	0.08	0	0	0	0.270	0.062	1.058
TCGA-D8-A1XT-01	0.054	0	0	0	0	0.107	0	0.048	0.022	0	0.023	0	0	0.332	0.123	0.256	0	0	0.036	0	0	0	0.010	0.365	0.948
TCGA-D8-A1XU-01	0.098	0	0.048	0.016	0	0.189	0	0.008	0.018	0	0.02	0	0.04	0	0.054	0.344	0.096	0	0.069	0	0	0	0.430	0.029	1.067
TCGA-D8-A1XV-01	0.088	0	0.055	0	0	0.2	0	0	0.018	0	0.028	0.025	0.01	0.065	0.024	0.374	0.009	0	0.103	0	0	0	0.760	-0.006	1.087
TCGA-D8-A1XW-01	0	0	0.014	0	0	0.254	0	0.025	0.022	0	0	0.032	0.028	0.154	0.085	0.274	0.004	0	0.108	0	0	0	0.040	0.160	1.029
TCGA-D8-A1XY-01	0.076	0	0.067	0	0	0.122	0	0.031	0.015	0	0	0.012	0	0.119	0.047	0.4	0.006	0.01	0.093	0	0	0	0.240	0.065	1.070
TCGA-D8-A1XZ-01	0.069	0	0.083	0	0	0.329	0	0.032	0.013	0	0.033	0	0.027	0.041	0.077	0.263	0	0	0.033	0	0	0	0.070	0.109	1.038
TCGA-D8-A1Y0-01	0.096	0	0.01	0	0	0.418	0	0.043	0	0	0.035	0	0.033	0	0.092	0.187	0.042	0.001	0.041	0	0	0	0.060	0.128	1.039
TCGA-D8-A1Y1-01	0.019	0	0.002	0	0	0.258	0	0	0	0	0.045	0	0.08	0.078	0.066	0.425	0	0	0.024	0	0	0.003	0.060	0.130	1.051
TCGA-D8-A1Y2-01	0.001	0	0	0.028	0	0.071	0	0	0.041	0	0	0.007	0	0.522	0.047	0.22	0	0	0.063	0	0	0	0.000	0.730	0.687
TCGA-D8-A1Y3-01	0.032	0	0.013	0.058	0	0.086	0.001	0	0.006	0	0	0	0.022	0.041	0.073	0.55	0	0	0.119	0	0	0	0.010	0.315	0.969
TCGA-D8-A27E-01	0.031	0	0.027	0.012	0	0.256	0	0.038	0	0	0.004	0.023	0.103	0	0.078	0.34	0.033	0	0.056	0	0	0	0.740	-0.004	1.088
TCGA-D8-A27F-01	0.059	0	0.017	0.022	0	0.167	0	0	0	0	0.028	0.005	0.008	0.549	0.011	0.112	0	0.007	0	0.015	0	0	0.010	0.334	0.989
TCGA-D8-A27G-01	0.05	0	0	0	0	0.311	0	0.029	0.004	0	0.017	0.013	0.05	0.143	0.088	0.152	0.009	0	0.124	0	0.01	0	0.040	0.148	1.026
TCGA-D8-A27H-01	0.06	0	0.017	0	0	0.387	0	0.032	0.049	0	0.004	0.014	0.064	0.264	0.022	0.05	0	0	0.038	0	0	0	0.200	0.078	1.069
TCGA-D8-A27I-01	0.114	0	0.045	0.064	0	0.334	0	0	0	0	0.035	0	0.019	0	0.061	0.204	0.054	0	0.069	0	0	0	0.050	0.141	1.024

TCGA-D8-A27K-01	0.18	0	0.053	0	0	0.315	0	0.04	0.011	0	0.082	0.006	0.052	0.028	0.074	0.107	0	0	0.048	0	0.004	0	0.390	0.038	1.068
TCGA-D8-A27L-01	0.11	0	0.032	0	0	0.269	0	0.028	0.024	0	0.043	0	0.033	0.061	0.057	0.192	0.01	0	0.14	0	0	0	0.070	0.116	1.030
TCGA-D8-A27M-01	0.149	0	0.065	0.027	0	0.224	0	0.053	0	0	0.049	0.003	0.019	0.026	0.159	0.185	0	0	0.04	0	0	0	0.010	0.329	0.951
TCGA-D8-A27N-01	0.07	0	0.032	0.025	0	0.137	0	0.004	0.027	0	0.024	0	0	0.335	0.047	0.26	0	0	0.039	0	0	0	0.010	0.344	0.955
TCGA-D8-A27P-01	0	0.001	0.019	0	0	0.282	0	0.021	0.044	0	0.065	0.008	0.011	0.443	0	0.007	0.008	0	0.091	0	0	0	0.290	0.059	1.106
TCGA-D8-A27R-01	0.015	0	0.122	0.103	0.022	0.166	0	0	0.006	0	0	0.013	0.056	0	0.041	0.326	0	0.008	0.122	0	0	0	0.040	0.177	1.007
TCGA-D8-A27T-01	0.018	0	0	0.149	0	0.209	0	0.027	0.009	0	0	0.005	0.047	0.175	0.095	0.125	0.004	0	0.137	0	0	0	0.060	0.128	1.041
TCGA-D8-A27V-01	0.12	0	0.01	0	0	0.353	0	0.013	0	0	0.021	0.021	0.038	0	0.1	0.21	0.017	0	0.096	0	0	0	0.740	-0.005	1.087
TCGA-D8-A27W-01	0.141	0	0.207	0.05	0	0.11	0	0	0.022	0	0	0.025	0.002	0.11	0.032	0.212	0.003	0	0.085	0	0	0	0.050	0.140	1.016
TCGA-D8-A3Z5-01	0.09	0	0.275	0.087	0	0.15	0	0.027	0.035	0	0.051	0.014	0.028	0	0.035	0.169	0	0	0	0.039	0	0	0.740	-0.004	1.067
TCGA-D8-A3Z6-01	0.003	0	0.192	0	0	0.252	0	0.014	0.089	0	0.078	0.024	0.042	0.116	0	0.017	0	0.002	0.171	0	0	0	0.600	0.016	1.069
TCGA-D8-A4Z1-01	0.081	0	0.091	0.025	0	0.309	0	0.039	0	0	0.049	0.03	0.035	0	0.056	0.163	0	0	0.123	0	0	0	0.310	0.054	1.055
TCGA-D8-A73U-01	0.035	0	0.052	0.105	0	0.281	0	0.025	0.041	0	0.054	0.012	0.022	0.091	0.067	0.163	0.004	0	0.047	0	0	0	0.010	0.326	0.951
TCGA-D8-A73W-01	0.052	0	0.002	0	0	0.158	0	0.026	0.072	0	0.033	0	0.034	0.414	0.046	0.127	0	0	0.035	0	0	0	0.610	0.013	1.127
TCGA-D8-A73X-01	0.001	0.006	0.009	0	0	0.186	0	0.007	0.05	0	0.041	0.007	0	0.415	0	0.088	0	0	0.191	0	0	0	0.040	0.194	1.038
TCGA-E2-A105-01	0.035	0	0.006	0.031	0	0.29	0	0.039	0.071	0	0.011	0	0	0.389	0.026	0.073	0	0	0.024	0	0	0.006	0.340	0.047	1.108
TCGA-E2-A106-01	0.11	0	0.225	0.043	0	0.093	0	0.035	0	0	0	0.048	0.005	0.125	0.079	0.123	0	0	0.114	0	0	0	0.790	-0.012	1.069
TCGA-E2-A107-01	0.085	0	0.028	0.087	0	0.213	0	0.005	0.052	0	0.009	0.013	0.03	0.02	0.01	0.293	0	0.04	0.115	0	0	0	0.720	0.001	1.078
TCGA-E2-A108-01	0.071	0	0	0.103	0	0.267	0.01	0.056	0.005	0	0.006	0.023	0.029	0.099	0.087	0.126	0	0	0.12	0	0	0	0.010	0.340	0.944
TCGA-E2-A109-01	0.094	0	0.107	0.029	0	0.226	0	0	0.034	0	0.02	0	0	0.251	0.063	0.143	0	0	0.033	0	0	0	0.070	0.115	1.042
TCGA-E2-A10A-01	0.021	0	0.05	0	0	0.102	0	0.007	0.042	0.016	0	0.02	0	0.413	0.042	0.242	0	0	0.046	0	0	0	0.010	0.315	0.982
TCGA-E2-A10B-01	0.081	0	0.001	0	0	0.207	0	0.046	0.059	0	0.027	0.03	0.036	0.033	0.05	0.293	0.017	0	0.119	0	0	0	0.800	-0.012	1.084
TCGA-E2-A10C-01	0.105	0	0.004	0.065	0	0.288	0	0.002	0.065	0	0.038	0	0.014	0.243	0.047	0.098	0	0	0.03	0	0	0	0.130	0.093	1.060
TCGA-E2-A10E-01	0.122	0	0.065	0.017	0	0.278	0	0.053	0.003	0	0.014	0.023	0.023	0.052	0.12	0.136	0.03	0	0.063	0	0	0	0.660	0.008	1.078
TCGA-E2-A10F-01	0.071	0	0.006	0.015	0	0.304	0	0.009	0.018	0	0.019	0.011	0.017	0.144	0.065	0.228	0.01	0	0.084	0	0	0	0.040	0.168	1.020
TCGA-E2-A14N-01	0.061	0	0	0.045	0	0.415	0.035	0.021	0	0	0.044	0	0.012	0.088	0.181	0.082	0	0.017	0	0	0	0	0.020	0.247	1.007
TCGA-E2-A14O-01	0.028	0	0.02	0.029	0	0.133	0	0.014	0.026	0	0	0.019	0	0.482	0.063	0.144	0	0	0.042	0	0	0	0.040	0.150	1.083
TCGA-E2-A14P-01	0.134	0	0.064	0.034	0	0.266	0	0.016	0.014	0	0.052	0.001	0.058	0.152	0.128	0.03	0.008	0	0.044	0	0	0	0.040	0.171	1.018
TCGA-E2-A14Q-01	0.113	0	0.053	0.056	0	0.411	0	0	0	0	0.014	0	0.02	0	0.1	0.125	0.025	0	0.083	0	0	0	0.450	0.024	1.081
TCGA-E2-A14R-01	0.002	0	0.099	0	0	0.189	0	0.051	0.018	0.043	0	0	0	0.389	0.137	0.03	0	0	0.045	0	0	0	0.010	0.408	0.919
TCGA-E2-A14T-01	0.077	0	0.006	0	0	0.047	0	0.025	0.024	0	0.022	0	0	0.624	0.02	0.126	0	0	0.029	0	0	0	0.020	0.223	1.083
TCGA-E2-A14U-01	0.175	0	0.058	0.072	0	0.26	0	0.007	0	0	0.041	0	0.009	0.026	0.024	0.322	0	0	0	0.007	0	0	0.660	0.006	1.085
TCGA-E2-A14V-01	0.115	0	0	0.034	0	0.244	0	0.019	0.04	0	0.015	0.002	0	0.167	0.073	0.164	0	0.005	0.124	0	0	0	0.500	0.021	1.078
TCGA-E2-A14W-01	0.081	0	0.27	0	0	0.117	0	0.017	0.04	0	0.003	0.009	0	0.318	0.019	0.116	0	0	0.007	0	0.003	0	0.380	0.040	1.078
TCGA-E2-A14X-01	0.15	0	0.167	0.045	0	0.3	0.002	0.035	0	0	0.041	0	0.018	0.049	0.072	0.118	0	0	0.003	0	0	0	0.070	0.114	1.034
TCGA-E2-A14Y-01	0.002	0	0.057	0	0	0.147	0	0.076	0	0	0.058	0	0	0.335	0.035	0.098	0	0.159	0.033	0	0	0	0.200	0.078	1.083
TCGA-E2-A14Z-01	0.081	0	0	0	0	0.302	0.003	0.042	0.042	0	0.041	0.015	0.062	0.084	0.086	0.136	0.004	0.012	0.092	0	0	0	0.010	0.283	0.968
TCGA-E2-A150-01	0.063	0	0	0	0	0.179	0	0.054	0.064	0	0.05	0	0.001	0.384	0.098	0.095	0	0	0.008	0	0	0	0.010	0.257	1.003
TCGA-E2-A152-01	0.066	0	0.08	0	0	0.149	0	0.001	0.029	0	0	0.034	0	0.262	0.033	0.273	0	0	0.072	0	0	0	0.030	0.211	1.010
TCGA-E2-A153-01	0	0.003	0	0	0	0.237	0	0.017	0.061	0	0.029	0.01	0.017	0.403	0	0.138	0	0.004	0.081	0	0	0	0.030	0.209	1.031
TCGA-E2-A153-11	0.109	0	0.066	0.007	0	0.247	0	0	0	0	0.054	0	0.004	0.185	0.03	0.265	0.013	0.001	0.018	0	0	0	0.050	0.143	1.032
TCGA-E2-A154-01	0	0	0.045	0	0	0.322	0	0	0.061	0	0.11	0	0.032	0.235	0	0.006	0	0.015	0.193	0	0	0	0.950	-0.026	1.106
TCGA-E2-A155-01	0.181	0	0.13	0.004	0	0.235	0	0.026	0.003	0	0	0.018	0.027	0.101	0.045	0.028	0.002	0	0.199	0	0	0	0.720	-0.001	1.073
TCGA-E2-A156-01	0.038	0	0.048	0	0	0.092	0	0	0.05	0	0	0.045	0	0.152	0	0.431	0	0.03	0.113	0	0	0	0.410	0.035	1.096
TCGA-E2-A158-01	0.054	0	0.012	0.082	0	0.144	0	0.19	0	0	0.011	0.035	0.062	0.135	0.121	0.096	0	0.006	0.043	0	0.01	0	0.640	0.011	1.090
TCGA-E2-A158-11	0.156	0	0.009	0	0	0.31	0	0	0	0	0	0.081	0.078	0	0.002	0.24	0	0.006	0.114	0	0.004	0	0.470	0.023	1.075
TCGA-E2-A159-01	0.063	0	0.07	0.092	0	0.293	0.032	0.002	0.007	0	0.047	0	0.008	0.112	0.12	0.119	0.003	0.001	0.03	0	0	0	0.010	0.349	0.941
TCGA-E2-A15A-01	0.006	0	0.007	0.077	0	0.166	0	0.033	0	0	0	0.048	0.03	0.136	0.132	0.205	0.007	0	0.153	0	0	0	0.040	0.159	1.031
TCGA-E2-A15A-06	0.105	0.017	0	0.111	0	0.408	0	0	0	0	0.053	0	0.042	0.009	0.106	0.107	0.024	0	0.02	0	0	0	0.010	0.330	0.956
TCGA-E2-A15C-01	0.163	0	0.108	0.055	0	0.258	0	0.037	0	0	0.035	0	0.004	0.052	0.062	0.177	0	0.001	0.047	0	0	0	0.690	0.004	1.073
TCGA-E2-A15D-01	0.083	0	0.039	0.086	0	0.258	0	0.089	0	0	0	0.025	0.026	0.087	0.064	0.187	0.006	0	0.051	0	0	0	0.430	0.029	1.073
TCGA-E2-A15E-01	0.097	0	0.044	0.063	0	0.165	0	0.002	0	0	0	0.023	0.022	0.059	0.057	0.307	0	0	0.151	0	0	0	0.720	-0.003	1.082
TCGA-E2-A15E-06	0.191	0.07	0	0.06	0	0.35	0	0	0.044	0	0.025	0	0.036	0	0.035	0.148	0	0.003	0.038	0	0	0	0.220	0.070	1.068
TCGA-E2-A15F-01	0.17	0	0.023	0.016	0	0.255	0	0.003	0.058	0	0.073	0	0	0.231	0	0.075	0	0.045	0.052	0	0	0	0.260	0.063	1.065
TCGA-E2-A15G-01	0.067	0	0.02	0.036	0	0.171	0	0.028	0.004	0	0.01	0.013	0.093	0	0.033	0.42	0.003	0	0.104	0	0	0	0.890	-0.020	1.098
TCGA-E2-A15H-01	0.121	0	0.131	0.047	0	0.18	0	0.007	0.024	0	0	0.045	0.019	0.183	0.052	0.122	0	0	0.07	0	0	0	0.590	0.017	1.067
TCGA-E2-A15I-01	0.173	0	0.004	0.044	0	0.387	0	0	0	0	0.019	0	0.016	0.105	0.069	0.143	0	0	0.041	0	0	0	0.310	0.051	1.073

TCGA-E2-A15I-11	0.235	0	0.08	0.008	0	0.296	0	0	0	0	0	0.05	0.036	0	0.005	0.214	0	0.022	0.05	0	0.004	0	0.720	-0.000	1.080
TCGA-E2-A15J-01	0.029	0	0	0.029	0	0.069	0	0.112	0.025	0	0	0.053	0.003	0.307	0	0.239	0	0.092	0.043	0	0	0	0.590	0.017	1.114
TCGA-E2-A15K-01	0.16	0	0	0.058	0	0.317	0	0.017	0.018	0	0.042	0	0.027	0.146	0.071	0.116	0	0	0.027	0	0	0	0.040	0.188	1.014
TCGA-E2-A15K-06	0.047	0	0.137	0	0	0.14	0	0.003	0.015	0	0.031	0.003	0.022	0	0	0.425	0.006	0.024	0.14	0.005	0	0.001	0.600	0.015	1.076
TCGA-E2-A15K-11	0.007	0	0.013	0.068	0	0.179	0	0.012	0.03	0	0	0.097	0.113	0.072	0.025	0.299	0.005	0	0.08	0	0	0	0.150	0.087	1.058
TCGA-E2-A15L-01	0.035	0	0.169	0	0	0.152	0	0.006	0.02	0	0	0.034	0	0.196	0.022	0.258	0	0	0.109	0	0	0	0.600	0.015	1.078
TCGA-E2-A15M-01	0.069	0	0.014	0.008	0	0.51	0.018	0	0	0	0.031	0	0.029	0.039	0.053	0.197	0.012	0.008	0.014	0	0	0	0.010	0.278	0.977
TCGA-E2-A15M-11	0.127	0	0.007	0.043	0	0.31	0	0.005	0	0	0	0.057	0.038	0	0.014	0.253	0	0.009	0	0.137	0	0	0.690	0.005	1.095
TCGA-E2-A15O-01	0.048	0	0.007	0.012	0	0.244	0	0.02	0.056	0	0.025	0	0.014	0.317	0.04	0.145	0	0	0.073	0	0	0	0.160	0.087	1.071
TCGA-E2-A15P-01	0.131	0	0.103	0.042	0	0.179	0	0	0.02	0	0.016	0	0.012	0	0.079	0.284	0	0.01	0.124	0	0	0	0.950	-0.029	1.082
TCGA-E2-A15R-01	0.073	0	0.052	0.142	0	0.227	0	0.007	0	0	0	0.074	0.033	0	0.011	0.245	0	0	0.136	0	0	0	0.980	-0.040	1.101
TCGA-E2-A15S-01	0.085	0	0.173	0.028	0	0.089	0	0	0.051	0	0	0.041	0	0.295	0.015	0.161	0	0	0.062	0	0	0	0.820	-0.014	1.098
TCGA-E2-A15T-01	0.024	0	0.055	0	0	0.294	0	0.032	0.047	0	0.012	0	0	0.408	0	0.061	0.002	0	0.064	0	0	0	0.310	0.056	1.100
TCGA-E2-A1AZ-01	0.106	0	0.007	0.047	0	0.393	0.033	0.031	0	0	0.049	0	0.028	0.035	0.122	0.104	0	0.017	0.029	0	0	0	0.040	0.195	1.018
TCGA-E2-A1B0-01	0.049	0	0.016	0.044	0	0.252	0.003	0.012	0	0	0.028	0	0	0.263	0.069	0.154	0	0.077	0.033	0	0	0	0.720	-0.003	1.109
TCGA-E2-A1B1-01	0.111	0	0	0	0	0.327	0	0	0.002	0	0.009	0	0.023	0.231	0.113	0.116	0	0	0.069	0	0	0	0.010	0.296	0.969
TCGA-E2-A1B4-01	0.102	0	0.043	0.015	0	0.197	0	0.026	0.054	0	0	0.012	0	0.081	0.073	0.279	0	0	0.119	0	0	0	0.760	-0.009	1.083
TCGA-E2-A1B5-01	0.106	0.007	0.025	0.123	0	0.391	0.014	0	0.057	0	0.042	0	0.021	0.022	0.071	0.086	0	0.023	0.012	0	0	0	0.010	0.418	0.910
TCGA-E2-A1B6-01	0.106	0.062	0	0.053	0.001	0.304	0.013	0	0.085	0	0.111	0	0.037	0.092	0.096	0.025	0	0.013	0	0	0	0	0.000	0.493	0.869
TCGA-E2-A1BC-01	0.114	0	0.027	0	0	0.399	0	0.031	0	0	0	0.085	0.03	0.046	0.054	0.167	0.015	0	0.032	0	0	0	0.770	-0.011	1.097
TCGA-E2-A1BC-11	0.089	0	0	0.027	0	0.25	0	0	0	0	0.003	0.046	0.028	0	0.021	0.391	0	0	0.145	0	0	0	0.720	0.000	1.090
TCGA-E2-A1BD-01	0.064	0	0	0	0	0.216	0	0.038	0.055	0	0	0	0.005	0.101	0.084	0.346	0	0	0.09	0	0	0	0.420	0.030	1.083
TCGA-E2-A1IE-01	0.198	0	0.223	0	0	0.155	0	0	0.012	0	0	0.028	0.003	0.071	0.025	0.206	0	0	0.08	0	0	0	1.000	-0.056	1.084
TCGA-E2-A1IF-01	0.115	0	0.027	0.046	0	0.138	0	0.016	0.071	0	0.015	0	0	0.393	0.031	0.138	0	0	0.01	0	0	0	0.040	0.195	1.033
TCGA-E2-A1IG-01	0.117	0	0.071	0.002	0	0.206	0	0	0.053	0	0	0.004	0	0.181	0.062	0.284	0.018	0	0.002	0	0	0.001	0.680	0.005	1.093
TCGA-E2-A1IG-11	0	0	0	0.057	0	0.32	0	0.031	0	0	0.026	0.022	0.069	0.045	0.033	0.289	0	0.035	0.075	0	0	0	0.410	0.032	1.078
TCGA-E2-A1IH-01	0.015	0.036	0	0.111	0	0.251	0.015	0.069	0.001	0	0	0	0.034	0.26	0.1	0.062	0.011	0	0.034	0	0	0	0.010	0.330	0.955
TCGA-E2-A1II-01	0.012	0	0	0.048	0	0.389	0.031	0.073	0.043	0	0.008	0.013	0.012	0.059	0.167	0.089	0.011	0.003	0.04	0	0	0	0.010	0.363	0.943
TCGA-E2-A1IJ-01	0.028	0	0	0.043	0	0.249	0.013	0.017	0.028	0	0.055	0	0.042	0.067	0.095	0.129	0.012	0	0.221	0	0	0	0.050	0.143	1.029
TCGA-E2-A1IK-01	0.068	0	0.088	0.099	0	0.238	0.008	0.007	0.01	0	0.024	0	0	0.126	0.109	0.206	0	0	0.011	0.005	0	0	0.220	0.070	1.062
TCGA-E2-A1IL-01	0.171	0	0.084	0.005	0	0.138	0	0.021	0.018	0	0.014	0.012	0	0.12	0.088	0.277	0	0	0.051	0	0	0	0.960	-0.035	1.097
TCGA-E2-A1IN-01	0.086	0	0.032	0.004	0	0.24	0	0	0	0	0.019	0	0.034	0	0.04	0.4	0	0.023	0.119	0	0	0.004	0.400	0.037	1.070
TCGA-E2-A1IO-01	0.128	0	0.027	0.082	0	0.338	0	0.015	0.013	0	0.016	0	0.017	0.068	0.07	0.157	0	0.014	0.056	0	0	0	0.090	0.106	1.045
TCGA-E2-A1IU-01	0.155	0	0.108	0.029	0	0.225	0	0.041	0	0	0.014	0	0.018	0.043	0.097	0.199	0	0	0.071	0	0	0	0.500	0.021	1.065
TCGA-E2-A1L6-01	0.02	0	0.015	0	0	0.258	0	0.039	0	0	0.051	0	0	0.449	0	0.098	0.001	0	0.07	0	0	0	0.010	0.416	0.920
TCGA-E2-A1L7-01	0.054	0	0.019	0.176	0	0.312	0.008	0.014	0.018	0	0.019	0	0.013	0.089	0.08	0.181	0.006	0.007	0.004	0	0	0	0.030	0.210	1.011
TCGA-E2-A1L7-11	0.103	0	0.056	0.073	0	0.307	0	0	0	0	0.017	0.014	0.032	0	0.064	0.308	0.002	0	0.024	0	0	0	0.070	0.114	1.041
TCGA-E2-A1L8-01	0.16	0	0.057	0.039	0	0.203	0	0.046	0	0	0.006	0.004	0.016	0	0.036	0.154	0.117	0.119	0.045	0	0	0	0.340	0.047	1.056
TCGA-E2-A1L9-01	0.003	0	0	0	0	0.16	0	0.017	0.048	0	0.036	0	0	0.48	0.008	0.18	0	0.013	0.056	0	0	0	0.010	0.345	0.973
TCGA-E2-A1LA-01	0.052	0	0.082	0.101	0	0.112	0	0.055	0.029	0.004	0	0.081	0.015	0.213	0.081	0.105	0	0	0.071	0	0	0	0.010	0.281	0.971
TCGA-E2-A1LB-01	0	0	0.083	0	0	0.211	0	0.013	0.068	0	0.011	0.015	0.061	0.08	0.003	0.246	0	0.006	0.201	0	0	0	0.720	-0.002	1.079
TCGA-E2-A1LB-11	0.169	0	0.109	0.074	0	0.266	0	0.022	0.011	0	0.059	0	0.026	0.007	0.018	0.188	0	0.05	0	0	0	0	0.840	-0.016	1.085
TCGA-E2-A1LE-01	0.083	0	0	0.087	0	0.277	0	0.024	0.052	0	0.017	0	0.014	0.194	0.074	0.162	0	0	0.015	0	0	0	0.010	0.290	0.972
TCGA-E2-A1LG-01	0.067	0	0.176	0.042	0	0.142	0	0.086	0	0	0.015	0.004	0	0.112	0.177	0.135	0.008	0.003	0.032	0	0	0	0.220	0.071	1.063
TCGA-E2-A1LH-01	0.033	0	0.06	0.079	0	0.209	0	0.05	0.038	0	0	0.059	0.016	0.166	0.153	0.091	0.004	0	0.044	0	0	0	0.010	0.293	0.971
TCGA-E2-A1LH-11	0.017	0	0.011	0.134	0	0.237	0	0.008	0	0	0.028	0.002	0.052	0.187	0.039	0.087	0.014	0	0.186	0	0	0	0.300	0.057	1.068
TCGA-E2-A1LI-01	0.085	0	0.037	0	0	0.225	0	0.035	0.017	0	0.029	0.005	0.043	0.336	0.086	0.093	0	0	0.009	0	0	0	0.040	0.152	1.043
TCGA-E2-A1LK-01	0.006	0	0.006	0	0	0.129	0	0.015	0.004	0	0.05	0	0	0.603	0.009	0.121	0	0.057	0.002	0	0	0	0.220	0.069	1.174
TCGA-E2-A1LL-01	0.003	0	0.072	0.004	0	0.212	0	0.065	0	0	0	0.051	0.014	0.189	0.053	0.26	0.07	0	0.006	0	0	0	0.410	0.031	1.085
TCGA-E2-A1LS-01	0.13	0	0	0.102	0	0.223	0	0.043	0	0	0.05	0	0.045	0	0.077	0.26	0	0.068	0	0.002	0	0	0.700	0.003	1.092
TCGA-E2-A1LS-11	0.145	0	0	0	0	0.384	0	0	0	0	0	0.078	0.019	0	0.047	0.265	0	0	0	0.062	0	0	0.950	-0.026	1.109
TCGA-E2-A2P5-01	0.043	0	0.087	0	0	0.396	0	0.021	0.022	0	0	0.049	0.019	0.02	0.081	0.133	0.007	0	0.123	0	0	0	0.350	0.042	1.067
TCGA-E2-A2P6-01	0.127	0	0.06	0	0	0.163	0	0.023	0.019	0	0	0.01	0.032	0	0.067	0.383	0	0	0.116	0	0	0	0.210	0.076	1.052
TCGA-E2-A3DX-01	0.116	0	0.027	0.178	0	0.354	0.008	0	0	0	0	0	0.031	0.014	0.056	0.137	0.006	0.003	0.071	0	0	0	0.020	0.237	0.995
TCGA-E2-A56Z-01	0.072	0	0.12	0	0	0.157	0	0.01	0.082	0	0	0.047	0	0.255	0.076	0.142	0	0	0.04	0	0	0	0.210	0.076	1.059
TCGA-E2-A570-01	0.163	0	0.072	0.025	0	0.209	0	0	0.033	0	0	0.025	0	0.084	0.019	0.271	0	0.011	0.083	0	0	0.007	0.740	-0.004	1.077
TCGA-E2-A572-01																									

	0.013	0	0.258	0.063	0	0.149	0	0.008	0.033	0	0.092	0	0.06	0.091	0.012	0.02	0	0.012	0.185	0	0.003	0	0.230	0.067	1.042
TCGA-E2-A573-01	0.066	0	0.123	0	0	0.117	0	0.072	0.004	0	0.01	0.045	0	0.349	0.082	0.092	0	0.009	0.031	0	0	0	0.010	0.283	0.978
TCGA-E2-A574-01	0.069	0	0	0.014	0	0.204	0	0.033	0.084	0	0.02	0	0	0.444	0	0.128	0	0	0.004	0	0	0	0.260	0.062	1.117
TCGA-E2-A576-01	0.009	0	0.103	0.087	0	0.134	0	0.053	0.032	0	0	0	0.017	0.094	0.037	0.053	0	0.014	0.367	0	0	0	0.190	0.082	1.066
TCGA-E2-A9RU-01	0	0	0.015	0.014	0	0.135	0	0.034	0	0	0.01	0.009	0.003	0.382	0.111	0.229	0	0	0.058	0	0	0	0.020	0.228	1.032
TCGA-E9-A1N3-01	0.088	0	0.2	0.012	0	0.11	0	0.015	0.022	0	0.001	0.006	0.014	0	0.035	0.428	0.011	0	0.05	0	0	0.008	0.960	-0.036	1.093
TCGA-E9-A1N4-01	0.018	0.004	0	0	0.046	0.105	0.001	0	0.006	0.016	0.038	0	0.021	0.446	0.086	0.121	0	0	0.091	0	0	0	0.000	0.695	0.730
TCGA-E9-A1N4-11	0.095	0	0.007	0.056	0	0.432	0	0	0	0	0.049	0.005	0.022	0.042	0.007	0.251	0	0.019	0	0.016	0	0	0.260	0.062	1.075
TCGA-E9-A1N5-01	0.065	0	0.001	0.053	0	0.213	0	0.024	0.052	0	0	0.057	0.021	0.196	0.035	0.196	0	0	0.088	0	0	0	0.040	0.175	1.019
TCGA-E9-A1N5-11	0.095	0	0.072	0	0	0.117	0	0.08	0	0	0	0.056	0	0.246	0	0.294	0	0.008	0.031	0	0	0	0.070	0.119	1.053
TCGA-E9-A1N6-01	0.035	0	0	0	0	0.125	0	0.032	0.024	0	0.009	0.002	0	0.401	0.045	0.302	0	0	0.026	0	0	0	0.010	0.355	0.965
TCGA-E9-A1N6-11	0.008	0	0.086	0.099	0	0.226	0	0	0	0	0.037	0.028	0.072	0.044	0.006	0.298	0.003	0.01	0.06	0	0.024	0	0.240	0.067	1.055
TCGA-E9-A1N8-01	0.076	0	0	0	0	0.177	0	0.106	0.019	0	0	0.043	0	0.362	0.134	0.077	0	0	0.007	0	0	0	0.300	0.057	1.104
TCGA-E9-A1N9-01	0.132	0	0.049	0.049	0	0.4	0.008	0	0	0	0.014	0	0.026	0.051	0.095	0.147	0.012	0	0.017	0	0	0	0.350	0.045	1.074
TCGA-E9-A1N9-11	0.126	0	0.149	0.083	0	0.251	0	0.014	0	0	0	0.04	0.062	0.003	0.003	0.19	0	0.019	0.057	0	0.004	0	0.680	0.005	1.068
TCGA-E9-A1NA-01	0.023	0	0	0.011	0	0.192	0	0.004	0	0	0.016	0.005	0.132	0.149	0.04	0.33	0	0.062	0.035	0	0	0	0.030	0.199	1.018
TCGA-E9-A1NA-11	0.029	0	0.077	0.129	0	0.198	0	0	0	0	0	0.014	0.12	0	0.015	0.243	0	0	0.157	0	0	0.019	0.280	0.060	1.053
TCGA-E9-A1ND-01	0.009	0	0	0.014	0	0.215	0.003	0.106	0.006	0	0	0.053	0.003	0.119	0.298	0.113	0.003	0	0.055	0	0	0.003	0.010	0.428	0.922
TCGA-E9-A1ND-11	0.012	0	0.023	0	0	0.062	0	0	0.015	0	0	0.02	0.013	0.274	0.013	0.47	0.008	0	0.088	0	0	0	0.000	0.458	0.899
TCGA-E9-A1NE-01	0.126	0	0	0.047	0	0.471	0	0.025	0	0	0.055	0.01	0.054	0.019	0.106	0.05	0	0	0.035	0	0.002	0	0.010	0.310	0.968
TCGA-E9-A1NF-01	0.055	0	0.015	0.012	0	0.126	0	0.006	0.026	0	0.026	0	0	0.395	0.059	0.258	0	0	0.022	0	0	0	0.000	0.507	0.864
TCGA-E9-A1NF-11	0.037	0	0.002	0	0	0.248	0	0.001	0.001	0	0.008	0.073	0.053	0	0.003	0.464	0	0	0.104	0	0.005	0	0.040	0.168	1.029
TCGA-E9-A1NG-01	0.035	0	0.003	0	0	0.202	0	0.004	0.012	0	0	0.032	0.02	0.225	0	0.38	0	0.041	0.044	0	0	0.001	0.010	0.430	0.906
TCGA-E9-A1NG-11	0.059	0	0.006	0	0	0.238	0	0	0.007	0	0	0.07	0.076	0	0.015	0.401	0	0.004	0.117	0	0.007	0	0.310	0.055	1.069
TCGA-E9-A1NH-01	0.117	0	0.036	0.063	0	0.172	0	0.018	0.043	0	0.018	0	0.017	0.128	0.049	0.232	0.053	0	0.05	0	0	0.003	0.270	0.062	1.056
TCGA-E9-A1NI-01	0.081	0	0.037	0	0	0.106	0	0.018	0.015	0	0.005	0	0	0.385	0.032	0.289	0	0	0.03	0	0	0.003	0.030	0.199	1.047
TCGA-E9-A1QZ-01	0.02	0.043	0	0.042	0	0.458	0.002	0	0.007	0	0.022	0.012	0.033	0.062	0.122	0.058	0.009	0	0.107	0	0.003	0	0.010	0.308	0.965
TCGA-E9-A1R0-01	0.155	0	0.074	0.084	0	0.225	0	0	0.024	0	0.035	0	0.012	0.069	0.036	0.168	0	0.016	0.104	0	0	0	0.130	0.093	1.038
TCGA-E9-A1R2-01	0.126	0	0.059	0.081	0	0.325	0	0.031	0.028	0	0.039	0	0.019	0	0.089	0.161	0	0.016	0.027	0	0	0	0.220	0.070	1.063
TCGA-E9-A1R3-01	0.028	0	0	0.009	0	0.253	0	0	0	0	0.033	0	0.023	0.153	0.006	0.267	0.069	0.127	0.034	0	0	0	0.040	0.166	1.028
TCGA-E9-A1R4-01	0.01	0	0	0.028	0	0.181	0	0.001	0.107	0	0.05	0.017	0.032	0.101	0.085	0.264	0.013	0	0.11	0	0.002	0	0.030	0.220	0.998
TCGA-E9-A1R5-01	0.109	0	0.028	0	0	0.288	0	0	0.012	0	0	0	0	0.269	0.05	0.179	0	0	0.065	0	0	0	0.240	0.067	1.073
TCGA-E9-A1R6-01	0.103	0	0.029	0	0	0.043	0	0.035	0.062	0	0	0.017	0	0.335	0.045	0.247	0	0	0.083	0	0	0	0.320	0.050	1.101
TCGA-E9-A1R7-01	0.105	0	0.168	0	0	0.316	0	0	0	0	0	0.051	0.017	0.035	0.032	0.178	0	0	0.098	0	0	0	0.980	-0.040	1.081
TCGA-E9-A1R7-11	0.033	0	0.043	0	0	0.245	0	0.01	0	0	0	0.061	0.096	0	0.008	0.41	0	0.008	0.069	0	0.017	0	0.090	0.105	1.048
TCGA-E9-A1RA-01	0.126	0	0.002	0.056	0	0.191	0	0.021	0.042	0	0.016	0	0	0.215	0.062	0.229	0	0	0.04	0	0	0	0.090	0.106	1.053
TCGA-E9-A1RB-01	0	0.005	0	0	0	0.199	0	0.035	0.026	0	0.056	0	0	0.533	0.001	0.075	0	0.008	0.063	0	0	0	0.010	0.276	1.017
TCGA-E9-A1RB-11	0.064	0	0.04	0	0.023	0.324	0	0	0	0	0.061	0	0.011	0.008	0	0.266	0	0.011	0.06	0	0	0.131	0.070	0.107	1.044
TCGA-E9-A1RC-01	0	0.001	0.023	0	0	0.244	0	0.058	0.018	0	0.043	0.006	0	0.517	0	0	0	0.05	0.039	0	0	0	0.150	0.089	1.112
TCGA-E9-A1RC-11	0.064	0	0.005	0	0	0.272	0	0	0.007	0	0.026	0.088	0.138	0	0.027	0.254	0	0	0.118	0	0	0	0.410	0.037	1.072
TCGA-E9-A1RD-01	0.079	0	0.073	0	0	0.127	0	0.009	0.008	0	0.016	0.014	0.054	0	0.055	0.425	0.036	0	0.106	0	0	0	0.620	0.013	1.082
TCGA-E9-A1RD-11	0.083	0	0	0.031	0	0.116	0	0	0.003	0	0.019	0.004	0.001	0.168	0.024	0.374	0	0	0.177	0	0	0	0.070	0.115	1.058
TCGA-E9-A1RE-01	0.113	0	0.072	0.041	0	0.22	0	0.014	0.026	0	0.018	0	0	0.229	0.04	0.105	0	0	0.123	0	0	0	0.370	0.042	1.066
TCGA-E9-A1RF-01	0.011	0	0	0.079	0	0.253	0	0.059	0	0	0	0.021	0.029	0.086	0.153	0.222	0.019	0	0.068	0	0	0	0.040	0.184	1.023
TCGA-E9-A1RF-11	0.097	0	0.003	0	0	0.23	0	0.003	0.002	0	0	0.077	0	0	0.006	0.47	0	0.003	0.102	0	0.006	0	0.220	0.070	1.071
TCGA-E9-A1RG-01	0.092	0	0.013	0.037	0	0.112	0	0.02	0.081	0	0.004	0.02	0.028	0.084	0.09	0.326	0.026	0	0.066	0	0	0.001	0.040	0.183	1.014
TCGA-E9-A1RH-01	0.044	0	0.023	0	0	0.468	0	0.004	0	0	0.023	0.01	0.039	0.215	0.094	0.026	0.02	0	0.034	0	0	0	0.410	0.033	1.091
TCGA-E9-A1RH-11	0.05	0	0.026	0	0	0.339	0	0	0	0	0.007	0.098	0.062	0	0.015	0.198	0	0	0.204	0	0	0	0.630	0.011	1.085
TCGA-E9-A1RI-01	0.088	0	0.06	0	0	0.249	0	0	0	0	0.036	0	0	0.166	0.05	0.283	0	0	0.068	0	0	0	0.090	0.104	1.046
TCGA-E9-A1RI-11	0.002	0	0.006	0	0	0.515	0	0.013	0	0	0.008	0.035	0.088	0	0.016	0.224	0	0	0.085	0	0.006	0	0.580	0.018	1.093
TCGA-E9-A226-01	0.063	0	0.031	0	0	0.231	0	0.052	0.003	0	0	0.03	0	0.294	0.05	0.186	0	0	0.058	0	0	0	0.410	0.035	1.091
TCGA-E9-A227-01	0.074	0	0.025	0.084	0	0.379	0.013	0	0	0	0.014	0.003	0.043	0	0.041	0.178	0.062	0.047	0.039	0	0	0	0.040	0.171	1.017
TCGA-E9-A228-01	0.001	0.002	0.001	0	0	0.217	0	0.002	0.001	0	0.077	0	0	0.552	0.014	0.071	0.008	0	0.054	0	0	0	0.010	0.366	0.966
TCGA-E9-A229-01	0.066	0	0.027	0.002	0	0.271	0	0.008	0	0	0.045	0	0.065	0.078	0.094	0.273	0	0.01	0.062	0	0	0	0.190	0.084	1.052
TCGA-E9-A22A-01	0.025	0	0	0	0	0.359	0	0	0.042	0	0	0.024	0.004	0.176	0.157	0.075	0.006	0	0.125	0	0	0.006	0.010	0.272	0.984
TCGA-E9-A22B-01	0.09	0	0.015	0.034	0	0.243	0	0.04	0.008	0	0.014	0	0	0.077	0.097	0.243	0.054	0	0.052	0	0	0.033	0.220	0.071	1.057
TCGA-E9-A22D-01	0.069	0	0.017	0.17	0	0.216	0.085	0.022	0.037	0	0.032	0.008	0.017	0.053	0.093	0.114	0.006	0	0.061	0	0	0	0.010	0.383	0.925

TCGA-E9-A22E-01	0.069	0	0.006	0.051	0	0.179	0	0.013	0.06	0	0.018	0	0	0.25	0.088	0.211	0	0	0.055	0	0	0	0.010	0.310	0.964
TCGA-E9-A22G-01	0.025	0	0.008	0	0	0.126	0	0.088	0.035	0	0.028	0	0.011	0.311	0.073	0.082	0	0.21	0	0.003	0	0	0.030	0.215	1.028
TCGA-E9-A22H-01	0.143	0	0.107	0.034	0	0.187	0	0.004	0.032	0	0.005	0.011	0.006	0.006	0.038	0.329	0.004	0.007	0.087	0	0	0	0.500	0.022	1.064
TCGA-E9-A243-01	0.055	0	0.054	0.072	0	0.31	0.023	0	0.005	0	0.007	0.004	0.006	0.098	0.126	0.192	0	0	0.04	0	0	0.009	0.060	0.133	1.038
TCGA-E9-A244-01	0.061	0	0.017	0.047	0	0.248	0.004	0.054	0.014	0	0.04	0	0	0.255	0.1	0.159	0	0	0	0	0	0	0.070	0.112	1.060
TCGA-E9-A245-01	0.055	0	0.293	0	0	0.093	0	0.036	0	0	0	0.018	0.052	0	0.033	0.356	0	0	0.062	0	0	0.002	0.410	0.035	1.057
TCGA-E9-A247-01	0.124	0	0.048	0.006	0	0.164	0	0.007	0.035	0	0.003	0.014	0	0.174	0.054	0.222	0.013	0.046	0.09	0	0	0	0.820	-0.014	1.089
TCGA-E9-A248-01	0.036	0	0.155	0.016	0	0.315	0.044	0	0	0	0.003	0.031	0.007	0.147	0.083	0.08	0	0	0.082	0	0	0	0.010	0.336	0.944
TCGA-E9-A249-01	0.107	0	0	0	0	0.183	0	0.066	0.043	0	0.055	0.001	0.007	0.209	0.084	0.189	0	0	0.056	0	0	0	0.430	0.029	1.081
TCGA-E9-A24A-01	0.045	0	0.006	0	0	0.186	0	0.001	0	0	0.029	0	0.07	0.025	0.07	0.535	0	0	0.033	0	0	0	0.040	0.161	1.045
TCGA-E9-A295-01	0.107	0	0.091	0.004	0	0.196	0	0.002	0.064	0	0	0.022	0.03	0.047	0.085	0.189	0.014	0	0.149	0	0	0	0.310	0.051	1.051
TCGA-E9-A2JS-01	0.043	0	0.001	0.023	0	0.18	0	0.031	0.044	0	0.025	0.003	0.012	0.109	0.113	0.27	0	0	0.146	0	0	0	0.370	0.042	1.075
TCGA-E9-A2JT-01	0.306	0.121	0.004	0.144	0	0.209	0.01	0.023	0.017	0	0.021	0	0.007	0.029	0.044	0.055	0	0.009	0.003	0	0	0	0.000	0.509	0.860
TCGA-E9-A3Q9-01	0.078	0	0.079	0.088	0	0.157	0	0.014	0	0	0.001	0.018	0.147	0	0.033	0.35	0	0	0.035	0	0	0	0.190	0.082	1.050
TCGA-E9-A3QA-01	0.051	0	0.024	0	0	0.218	0.01	0.04	0.061	0	0.026	0.007	0.027	0.194	0.171	0.039	0	0.106	0.025	0	0	0	0.000	0.467	0.885
TCGA-E9-A3X8-01	0.013	0	0.163	0.135	0	0.126	0	0.037	0.062	0	0.006	0.027	0.023	0.077	0.102	0.1	0.016	0	0.112	0	0	0	0.020	0.231	0.985
TCGA-E9-A54X-01	0.152	0	0.233	0	0	0.056	0	0.093	0	0	0	0.028	0	0.287	0	0.127	0	0	0.024	0	0	0	0.890	-0.022	1.097
TCGA-E9-A54Y-01	0.177	0	0	0.067	0	0.113	0	0.024	0.117	0	0	0	0.008	0.236	0.02	0.212	0	0	0.026	0	0	0	0.220	0.069	1.071
TCGA-E9-A5FK-01	0.112	0	0.018	0.187	0	0.288	0	0.024	0.096	0	0.018	0.026	0.04	0.023	0.075	0.068	0	0	0.025	0	0	0	0.000	0.445	0.896
TCGA-E9-A5FL-01	0	0.001	0	0.022	0	0.154	0	0	0.068	0	0.014	0.015	0.026	0	0.025	0.587	0.018	0.034	0.036	0	0	0	0.050	0.145	1.056
TCGA-E9-A5UO-01	0.179	0	0.197	0	0	0.033	0	0.025	0.012	0	0.013	0.018	0.01	0.153	0	0.303	0	0.011	0.047	0	0	0	0.060	0.134	1.031
TCGA-E9-A5UP-01	0	0.007	0.001	0	0	0.124	0	0.064	0.102	0	0.069	0	0.026	0.485	0	0	0	0.092	0.029	0	0	0	0.060	0.135	1.079
TCGA-E9-A6HE-01	0.003	0	0.025	0.111	0	0.15	0	0.061	0.002	0	0	0.045	0.036	0.076	0.017	0.385	0	0.021	0.057	0	0	0.009	0.070	0.112	1.050
TCGA-EW-A1IW-01	0.135	0	0.01	0.021	0	0.304	0	0.019	0	0	0.04	0	0.027	0.048	0.083	0.186	0.008	0	0.06	0	0	0	0.110	0.097	1.048
TCGA-EW-A1IX-01	0.121	0	0.024	0.001	0	0.22	0	0.019	0.034	0	0.014	0	0	0.153	0.005	0.303	0	0.021	0	0.082	0	0.003	0.310	0.055	1.074
TCGA-EW-A1IY-01	0.03	0	0	0	0	0.514	0	0.04	0	0	0.034	0	0.022	0.009	0.186	0.066	0.023	0.002	0.061	0	0.012	0	0.010	0.317	0.971
TCGA-EW-A1IZ-01	0.117	0	0.08	0.209	0	0.206	0	0.058	0.023	0	0	0.069	0.016	0.025	0.109	0.066	0.001	0	0.019	0	0.001	0	0.010	0.361	0.940
TCGA-EW-A1J1-01	0.071	0	0.068	0.014	0	0.298	0	0.037	0	0	0.025	0.001	0.006	0.119	0.092	0.222	0	0	0.048	0	0	0	0.040	0.162	1.019
TCGA-EW-A1J2-01	0.056	0	0.005	0.046	0	0.306	0	0	0	0	0.034	0	0.008	0.223	0.066	0.195	0.008	0.009	0.045	0	0	0	0.050	0.141	1.041
TCGA-EW-A1J3-01	0.071	0	0.024	0	0	0.339	0	0.022	0	0	0	0.009	0.007	0.026	0.101	0.317	0	0.012	0	0.068	0	0.004	0.430	0.029	1.084
TCGA-EW-A1J5-01	0.051	0	0.015	0	0	0.325	0.033	0.004	0.005	0	0.086	0	0.017	0.07	0.116	0.165	0	0	0.112	0	0	0	0.040	0.171	1.018
TCGA-EW-A1J6-01	0.137	0	0.04	0.004	0	0.308	0.022	0.056	0	0	0.048	0	0.022	0	0.099	0.245	0	0.007	0.014	0	0	0	0.040	0.181	1.013
TCGA-EW-A1OV-01	0.128	0	0.022	0.126	0	0.331	0.013	0.015	0.016	0	0.023	0	0.015	0.101	0.102	0.091	0	0	0.017	0	0	0	0.010	0.427	0.904
TCGA-EW-A1OW-01	0.105	0	0.071	0.021	0	0.17	0	0.04	0.039	0	0.039	0	0	0.267	0.076	0.143	0.017	0	0.011	0	0	0	0.040	0.159	1.028
TCGA-EW-A1OX-01	0.159	0	0.375	0	0	0.07	0	0.022	0.046	0	0.018	0.002	0	0.201	0	0.04	0	0	0.067	0	0	0	0.720	-0.000	1.071
TCGA-EW-A1OY-01	0.08	0	0.051	0.015	0	0.248	0	0.03	0.038	0	0.016	0	0.012	0.193	0.129	0.158	0	0	0.03	0	0	0	0.200	0.078	1.064
TCGA-EW-A1OZ-01	0.063	0	0.003	0.022	0	0.176	0	0.037	0.078	0	0.008	0	0.028	0.335	0.007	0.158	0	0	0.086	0	0	0	0.040	0.160	1.040
TCGA-EW-A1P0-01	0.074	0	0.046	0.035	0	0.285	0.004	0.028	0.028	0	0.022	0	0.006	0.188	0.064	0.196	0.006	0	0.017	0	0	0	0.180	0.084	1.058
TCGA-EW-A1P1-01	0.002	0	0	0.001	0	0.294	0	0.074	0	0	0	0.009	0.059	0.368	0.094	0.078	0.009	0	0.074	0	0	0	0.040	0.175	1.032
TCGA-EW-A1P3-01	0.104	0	0.059	0.027	0	0.253	0	0.038	0	0	0	0.004	0.004	0	0.108	0.318	0.007	0	0.078	0	0	0	0.450	0.024	1.075
TCGA-EW-A1P4-01	0.055	0	0	0.089	0	0.241	0.067	0.037	0	0	0.069	0.059	0.028	0.071	0.086	0.074	0.044	0.012	0.062	0	0	0.005	0.040	0.184	1.018
TCGA-EW-A1P5-01	0.064	0	0.009	0.022	0	0.129	0	0.043	0.033	0	0.013	0.015	0	0.267	0.04	0.25	0	0.048	0.066	0	0	0.002	0.010	0.311	0.964
TCGA-EW-A1P6-01	0.116	0	0.112	0.054	0	0.188	0	0.023	0	0	0.021	0	0.004	0.006	0.051	0.34	0	0	0.084	0	0	0	0.890	-0.022	1.081
TCGA-EW-A1P7-01	0.177	0.093	0.02	0.134	0	0.227	0.013	0.014	0.006	0	0.018	0	0.008	0.125	0.072	0.075	0	0.01	0.009	0	0	0	0.010	0.436	0.899
TCGA-EW-A1P8-01	0.012	0	0.005	0.033	0	0.17	0	0.034	0.03	0	0.014	0	0.103	0.092	0.055	0.338	0.105	0	0	0.01	0	0	0.020	0.239	0.996
TCGA-EW-A1PA-01	0.057	0	0.114	0	0	0.282	0	0.03	0	0	0.038	0	0	0.12	0.101	0.142	0	0	0.117	0	0	0	0.220	0.072	1.049
TCGA-EW-A1PB-01	0.05	0	0	0.078	0	0.134	0	0.059	0.019	0	0.043	0.009	0.007	0.039	0.329	0.164	0	0	0.069	0	0	0	0.000	0.446	0.915
TCGA-EW-A1PC-01	0.062	0	0	0.036	0	0.103	0	0.131	0.019	0	0	0.038	0.002	0.017	0.009	0.277	0	0.26	0.032	0	0	0.013	0.810	-0.013	1.107
TCGA-EW-A1PD-01	0.072	0	0.003	0	0	0.242	0	0.03	0.048	0	0.011	0.058	0	0.37	0.009	0.118	0	0	0.04	0	0	0	0.150	0.088	1.082
TCGA-EW-A1PE-01	0.121	0	0.064	0.028	0	0.199	0	0.036	0.013	0	0.046	0	0.018	0.083	0.088	0.251	0	0.011	0.032	0	0	0.009	0.070	0.117	1.035
TCGA-EW-A1PF-01	0.109	0	0.075	0	0	0.239	0	0	0.022	0	0.039	0	0	0.236	0.06	0.177	0	0	0.043	0	0	0	0.050	0.145	1.030
TCGA-EW-A1PG-01	0.067	0	0.059	0.032	0	0.299	0.001	0	0	0	0.039	0	0.034	0.088	0.02	0.199	0.003	0.097	0.052	0	0.009	0	0.320	0.050	1.058
TCGA-EW-A1PH-01	0.091	0	0.055	0.078	0	0.379	0.005	0.052	0	0	0.045	0	0.013	0.127	0.069	0.109	0	0.048	0	0	0	0	0.390	0.038	1.077
TCGA-EW-A2FR-01	0.052	0	0.054	0	0	0.082	0	0.027	0.042	0.003	0	0.014	0	0.397	0.062	0.198	0	0	0.071	0	0	0	0.010	0.286	0.991
TCGA-EW-A2FS-01	0.109	0	0.243	0.052	0	0.286	0	0.017	0.004	0	0.003	0.005	0.017	0.045	0.078	0.135	0.003	0	0.002	0	0	0	0.090	0.099	1.036
TCGA-EW-A2FV-01	0.011	0	0.014	0.037	0	0.393	0	0.009	0	0	0	0.026	0.048	0.087	0.106	0.151	0.004	0	0.114	0	0	0	0.040	0.181	1.017
TCGA-EW-A2FW-01	0.126	0	0.17	0	0.008	0.06	0	0.009	0.017	0	0.012	0	0	0.193	0.047	0.287	0	0	0.071	0	0	0	0.040	0.165	1.022

TCGA-EW-A3E8-01	0.051	0	0.027	0.071	0	0.33	0	0.002	0.034	0	0.022	0.004	0.054	0.095	0.128	0.034	0.009	0	0.138	0	0	0	0.010	0.319	0.955
TCGA-EW-A3U0-01	0.072	0	0.038	0.041	0	0.239	0.004	0.019	0.023	0	0.046	0.036	0.062	0.184	0.126	0.055	0.002	0	0.052	0	0	0	0.010	0.387	0.923
TCGA-EW-A423-01	0.063	0	0.028	0.039	0	0.22	0	0.015	0.075	0	0.014	0.011	0.009	0.094	0.054	0.304	0	0	0.044	0	0	0	0.060	0.134	1.035
TCGA-EW-A424-01	0.039	0	0.258	0.028	0	0.263	0	0.01	0.043	0	0	0.021	0.041	0.079	0	0.013	0	0	0.206	0	0	0	0.410	0.033	1.059
TCGA-EW-A6S9-01	0.087	0	0.089	0	0	0.118	0	0.031	0.032	0	0	0.018	0	0.299	0.073	0.179	0	0	0.075	0	0	0	0.200	0.079	1.068
TCGA-EW-A6SA-01	0.082	0	0.097	0	0	0.148	0	0.06	0.051	0	0	0	0	0.315	0.019	0.022	0	0	0.206	0	0	0	0.810	-0.013	1.100
TCGA-EW-A6SB-01	0.027	0	0.243	0.046	0	0.208	0	0.079	0.086	0	0	0.054	0.007	0.104	0.052	0.067	0.02	0	0.008	0	0	0	0.010	0.395	0.918
TCGA-EW-A6SC-01	0.069	0	0.06	0.01	0	0.227	0	0.018	0	0	0.011	0.002	0.024	0.057	0.042	0.272	0.074	0	0	0.11	0.023	0	0.090	0.100	1.043
TCGA-EW-A6SD-01	0.09	0	0.1	0.02	0	0.22	0	0.051	0.036	0	0.016	0.014	0.015	0.109	0.126	0.158	0.007	0	0.039	0	0	0	0.030	0.215	0.996
TCGA-GI-A2C8-01	0.064	0	0	0	0	0.112	0	0.02	0.019	0	0.037	0	0	0.334	0.034	0.327	0	0	0.053	0	0	0	0.020	0.244	1.017
TCGA-GI-A2C8-11	0.123	0	0	0	0	0.279	0	0	0	0	0.038	0.003	0.056	0	0.015	0.318	0	0.029	0.08	0	0.059	0	0.960	-0.032	1.091
TCGA-GI-A2C9-01	0.031	0	0	0	0	0.162	0	0.06	0.025	0	0	0.016	0.011	0.179	0.053	0.318	0.036	0.05	0.058	0	0	0	0.040	0.173	1.028
TCGA-GI-A2C9-11	0.182	0	0.063	0.079	0	0.296	0	0.024	0	0	0	0.011	0.068	0	0	0.2	0	0.04	0	0.035	0.003	0	0.450	0.024	1.073
TCGA-GM-A2D9-01	0.079	0	0.087	0	0	0.176	0	0.005	0.049	0	0	0.032	0.014	0.171	0.063	0.294	0	0	0.031	0	0	0	0.070	0.115	1.044
TCGA-GM-A2DA-01	0.071	0	0.058	0.165	0	0.365	0.016	0	0	0	0	0.017	0.039	0.046	0.134	0.007	0.008	0	0.081	0	0.004	0	0.040	0.185	1.027
TCGA-GM-A2DB-01	0.12	0	0.028	0.021	0	0.237	0	0.005	0.003	0	0.049	0	0	0.281	0.169	0.061	0	0	0.025	0	0	0	0.010	0.393	0.925
TCGA-GM-A2DC-01	0.143	0	0.037	0.075	0	0.165	0	0.047	0.037	0	0.013	0.054	0.022	0	0.071	0.213	0	0	0.123	0	0	0	0.950	-0.029	1.089
TCGA-GM-A2DD-01	0.038	0	0.003	0.091	0	0.401	0.031	0	0	0	0.044	0	0.019	0.075	0.103	0.133	0.012	0.027	0.024	0	0	0	0.040	0.191	1.021
TCGA-GM-A2DF-01	0.108	0	0.141	0.054	0	0.109	0	0.03	0.029	0	0	0.024	0	0.258	0.083	0.139	0	0	0.018	0	0.007	0	0.010	0.342	0.944
TCGA-GM-A2DH-01	0.146	0	0	0.011	0	0.253	0	0.067	0.034	0	0.043	0.039	0.038	0.121	0.088	0.057	0	0.011	0.086	0	0.007	0	0.010	0.271	0.974
TCGA-GM-A2DI-01	0.094	0	0.012	0.158	0	0.326	0.046	0	0	0	0.04	0	0.026	0.075	0.09	0.104	0	0.001	0.027	0	0	0	0.010	0.381	0.927
TCGA-GM-A2DK-01	0.096	0	0.022	0.051	0	0.114	0	0	0.026	0	0.006	0	0	0.357	0.036	0.263	0	0	0.028	0	0	0	0.020	0.236	1.017
TCGA-GM-A2DL-01	0.048	0	0.031	0	0	0.177	0	0.018	0.03	0	0.013	0.009	0.002	0.201	0.058	0.377	0.014	0.026	0.047	0	0	0	0.040	0.186	1.024
TCGA-GM-A2DM-01	0	0.005	0.001	0	0	0.38	0	0.015	0.072	0	0.014	0	0.051	0.293	0	0.153	0	0.033	0.021	0	0.012	0	0.410	0.033	1.100
TCGA-GM-A2DN-01	0.105	0	0.105	0.061	0	0.292	0	0	0	0	0.024	0	0.019	0.223	0.065	0.068	0	0.017	0.022	0	0	0	0.010	0.320	0.953
TCGA-GM-A2DO-01	0.212	0	0.027	0.153	0	0.295	0.015	0	0	0	0.025	0	0.007	0.122	0.084	0.047	0	0	0.013	0	0	0	0.000	0.499	0.866
TCGA-GM-A3NW-01	0.176	0	0.072	0.012	0	0.345	0	0	0.014	0	0	0	0.04	0.03	0.009	0.083	0	0.033	0.185	0	0	0	0.310	0.052	1.059
TCGA-GM-A3NY-01	0.085	0	0.118	0.023	0	0.28	0	0.05	0	0	0	0.068	0.019	0	0.119	0.175	0	0	0.064	0	0	0	0.610	0.014	1.078
TCGA-GM-A3XG-01	0.005	0	0	0.026	0	0.289	0	0.121	0	0	0.033	0.041	0	0.387	0.04	0.003	0	0	0.055	0	0	0	0.060	0.128	1.068
TCGA-GM-A3XL-01	0.034	0	0.102	0.12	0	0.147	0.049	0.035	0	0	0.05	0.018	0.014	0.063	0.128	0.193	0	0.01	0.038	0	0	0	0.010	0.307	0.958
TCGA-GM-A3XN-01	0.069	0	0.069	0.053	0	0.321	0	0.016	0	0	0.028	0	0.023	0.129	0.093	0.173	0	0	0.028	0	0	0	0.040	0.156	1.024
TCGA-GM-A4E0-01	0.088	0	0.017	0.105	0	0.299	0	0.013	0	0	0.042	0	0.023	0	0.072	0.303	0.007	0	0.03	0	0	0	0.040	0.149	1.030
TCGA-GM-A5PV-01	0.087	0	0.03	0.038	0	0.28	0	0.035	0.023	0	0	0.033	0.015	0.097	0.089	0.237	0.005	0	0.032	0	0	0	0.240	0.067	1.062
TCGA-GM-A5PX-01	0.108	0	0.02	0.079	0	0.315	0	0.02	0	0	0.011	0	0.026	0.05	0.024	0.226	0	0.005	0.115	0	0	0	0.240	0.067	1.056
TCGA-HN-A2NL-01	0.061	0	0.052	0.106	0	0.184	0	0.05	0.048	0	0	0.024	0	0.238	0.102	0.118	0	0	0.018	0	0	0	0.030	0.200	1.013
TCGA-HN-A2OB-01	0.098	0	0.058	0.05	0	0.26	0	0	0	0	0.019	0.011	0.023	0.029	0.025	0.227	0	0	0.2	0	0	0	0.350	0.044	1.060
TCGA-JL-A3YW-01	0.043	0	0.017	0.021	0	0.166	0	0.027	0.041	0	0.044	0	0	0.405	0.078	0.142	0	0	0.017	0	0	0	0.010	0.390	0.933
TCGA-JL-A3YX-01	0.032	0	0.229	0.069	0	0.134	0	0.028	0.027	0	0	0.028	0.039	0.05	0.005	0.1	0	0.006	0.252	0	0	0	0.200	0.078	1.042
TCGA-LD-A66U-01	0.012	0	0.057	0.208	0	0.216	0	0.03	0.112	0	0	0.003	0.047	0.055	0.081	0.105	0.002	0	0.069	0	0.003	0	0.040	0.182	1.018
TCGA-LD-A74U-01	0.059	0	0	0	0	0.071	0	0.008	0.034	0	0.012	0	0	0.465	0.025	0.224	0	0	0.099	0	0	0	0.000	0.477	0.888
TCGA-LD-A7W6-01	0.147	0	0.126	0.048	0	0.35	0	0.01	0.009	0	0.038	0	0.011	0	0.054	0.169	0.001	0.002	0.035	0	0	0	0.320	0.049	1.061
TCGA-LD-A9QF-01	0.045	0	0	0.064	0	0.125	0	0.046	0.079	0	0.011	0.001	0	0.451	0.057	0.109	0	0.001	0.011	0	0	0	0.000	0.743	0.692
TCGA-LL-A440-01	0.039	0	0	0.007	0	0.141	0	0.002	0.013	0	0.029	0	0.003	0.362	0.017	0.293	0.001	0	0.094	0	0	0	0.010	0.395	0.932
TCGA-LL-A441-01	0.36	0	0.001	0.028	0	0.253	0	0.022	0	0	0.046	0	0.021	0.176	0.058	0.024	0	0	0.013	0	0	0	0.000	0.561	0.831
TCGA-LL-A442-01	0.001	0	0.084	0	0	0.384	0	0.008	0.073	0	0.011	0	0.035	0	0	0.328	0	0.004	0.067	0	0	0.004	0.720	0.000	1.087
TCGA-LL-A50Y-01	0.075	0	0.02	0.069	0	0.157	0	0.005	0.038	0	0.019	0	0.002	0.146	0.024	0.33	0.047	0	0.069	0	0	0	0.030	0.206	1.007
TCGA-LL-A5YL-01	0.115	0	0.007	0.098	0	0.164	0	0.042	0.028	0	0	0.039	0.048	0	0.047	0.317	0	0	0.096	0	0	0	0.050	0.140	1.027
TCGA-LL-A5YM-01	0.002	0	0.095	0.012	0	0.013	0	0.006	0.033	0.016	0.021	0	0.007	0.436	0.051	0.194	0	0	0.114	0	0	0	0.000	0.500	0.869
TCGA-LL-A5YN-01	0.133	0	0	0.107	0	0.213	0	0.013	0.197	0	0	0.037	0	0.153	0.066	0.081	0	0	0	0	0	0.001	0.000	0.527	0.850
TCGA-LL-A5YO-01	0.115	0	0	0.088	0	0.177	0	0.023	0.105	0	0.016	0.031	0.036	0.2	0.132	0.048	0	0	0.029	0	0	0	0.010	0.256	0.989
TCGA-LL-A5YP-01	0.028	0	0.088	0.027	0	0.246	0	0.043	0	0	0.026	0	0.046	0.165	0.046	0.229	0.017	0.004	0.034	0	0	0	0.040	0.186	1.009
TCGA-LL-A6FP-01	0.003	0	0.05	0.064	0	0.242	0	0.023	0.067	0	0	0	0.08	0.153	0	0.049	0.043	0	0.223	0	0.003	0	0.450	0.024	1.075
TCGA-LL-A6FQ-01	0.038	0	0.008	0.016	0	0.081	0	0.004	0.028	0	0.014	0.001	0.071	0.392	0.022	0.299	0	0	0.026	0	0	0	0.000	0.669	0.747
TCGA-LL-A6FR-01	0.002	0.002	0.002	0	0	0.13	0	0.009	0.057	0	0.057	0	0	0.585	0	0.145	0	0	0.008	0	0	0	0.010	0.335	1.002
TCGA-LL-A73Y-01	0.092	0	0.016	0	0	0.227	0	0.015	0.033	0	0.023	0.002	0	0.45	0.016	0.063	0	0.053	0.008	0	0	0	0.000	0.539	0.842
TCGA-LL-A73Z-01	0.084	0	0.002	0.059	0	0.218	0	0.02	0.056	0	0	0.026	0.03	0.031	0.195	0.217	0	0	0.063	0	0	0	0.010	0.314	0.965
TCGA-LL-A740-01																									

	0.043	0	0	0	0	0.137	0.002	0.019	0.041	0	0.026	0	0.001	0.383	0.003	0.237	0.038	0.039	0.03	0	0	0	0.010	0.265	1.007
TCGA-LL-A7SZ-01	0.031	0	0.325	0.085	0	0.17	0.007	0.017	0.051	0	0.042	0	0.01	0.037	0.024	0.199	0	0	0.003	0	0	0	0.030	0.213	0.990
TCGA-LL-A7T0-01	0.028	0	0.129	0.017	0	0.087	0	0.032	0.039	0	0	0.024	0.008	0.359	0.076	0.143	0	0	0.06	0	0	0	0.020	0.245	1.000
TCGA-LL-A8F5-01	0.031	0	0.275	0	0	0.134	0.002	0.029	0.019	0.041	0	0.008	0	0.306	0.073	0.081	0	0	0	0	0	0	0.000	0.649	0.789
TCGA-LL-A9Q3-01	0.015	0	0.12	0.095	0	0.144	0	0	0.036	0	0	0.049	0.014	0.05	0.046	0.153	0	0.01	0.267	0	0	0	0.040	0.159	1.018
TCGA-LQ-A4E4-01	0.003	0	0.047	0	0	0.399	0	0.004	0.127	0	0	0	0.059	0.058	0	0.123	0	0	0.181	0	0	0	0.890	-0.021	1.100
TCGA-MS-A51U-01	0.095	0	0.077	0.047	0	0.396	0	0.013	0.003	0	0.047	0.004	0.053	0.037	0.081	0.103	0	0	0.039	0	0.004	0	0.220	0.070	1.060
TCGA-OK-A5Q2-01	0.207	0	0.01	0.083	0	0.401	0.004	0	0.008	0	0.032	0	0.014	0.044	0.046	0.116	0.014	0.006	0.015	0	0	0	0.020	0.239	0.996
TCGA-OL-A5D6-01	0.077	0	0.051	0.009	0	0.257	0	0	0	0	0.029	0	0.025	0.027	0.062	0.378	0.027	0	0.059	0	0	0	0.210	0.077	1.055
TCGA-OL-A5D7-01	0.039	0	0.035	0.096	0	0.312	0.025	0.033	0.099	0	0.059	0	0.031	0.084	0.129	0.034	0	0.016	0	0	0.008	0	0.030	0.213	1.016
TCGA-OL-A5D8-01	0.026	0	0.019	0.054	0	0.187	0	0.093	0.064	0	0.037	0.014	0.01	0.167	0.071	0.223	0	0	0.035	0	0	0	0.340	0.045	1.076
TCGA-OL-A5DA-01	0.093	0	0.074	0.107	0	0.244	0	0.061	0.068	0	0.016	0.001	0.013	0.024	0.078	0.155	0	0	0.065	0	0	0	0.310	0.054	1.062
TCGA-OL-A5RU-01	0.124	0	0.037	0.042	0.023	0.268	0	0	0.127	0	0.102	0	0.053	0.093	0.099	0.02	0.008	0	0	0	0.004	0	0.040	0.180	1.022
TCGA-OL-A5RV-01	0.123	0	0.121	0.029	0	0.317	0	0	0	0	0.021	0.001	0.012	0	0.027	0.19	0.006	0	0.153	0	0	0	0.410	0.034	1.058
TCGA-OL-A5RW-01	0.056	0	0.031	0.019	0	0.128	0	0.065	0.029	0	0	0.023	0.008	0.149	0.058	0.279	0	0.128	0.029	0	0	0	0.060	0.129	1.044
TCGA-OL-A5RX-01	0.2	0	0.091	0.081	0	0.319	0	0.024	0	0	0.047	0	0.024	0	0.04	0.132	0.023	0	0.018	0	0	0	0.210	0.076	1.056
TCGA-OL-A5RY-01	0.213	0	0.011	0.034	0	0.286	0.005	0.008	0.009	0	0.042	0	0.034	0.028	0.084	0.213	0.001	0	0.033	0	0	0	0.010	0.434	0.900
TCGA-OL-A5RZ-01	0.072	0	0.096	0.019	0	0.131	0	0.015	0	0	0.051	0	0.016	0.286	0.014	0.279	0	0	0.02	0	0	0	0.750	-0.006	1.114
TCGA-OL-A5S0-01	0.023	0	0	0	0	0.191	0	0.009	0.048	0	0.034	0	0	0.37	0.125	0.183	0	0	0.017	0	0	0	0.010	0.390	0.935
TCGA-OL-A66H-01	0.001	0	0.087	0	0	0.305	0	0	0.065	0	0.008	0	0.06	0.065	0.081	0.242	0.001	0	0.08	0	0.005	0	0.460	0.023	1.070
TCGA-OL-A66I-01	0	0	0.013	0.197	0	0.314	0.012	0	0.067	0	0	0.022	0.016	0.098	0.12	0.052	0.044	0.002	0.044	0	0	0	0.000	0.461	0.887
TCGA-OL-A66J-01	0.075	0	0.025	0.052	0	0.108	0	0.031	0.014	0	0.029	0.006	0	0.298	0.04	0.282	0	0	0.04	0	0	0	0.040	0.163	1.043
TCGA-OL-A66K-01	0.155	0	0.072	0.013	0	0.138	0	0.064	0.001	0	0	0.027	0	0.092	0.006	0.305	0	0.036	0.09	0	0	0	0.420	0.030	1.066
TCGA-OL-A66L-01	0.084	0	0.072	0.111	0	0.256	0	0.024	0.012	0	0	0.025	0.041	0.004	0.053	0.249	0	0.016	0.053	0	0	0	0.210	0.074	1.051
TCGA-OL-A66N-01	0.028	0	0.107	0.137	0	0.198	0	0	0.037	0	0	0.024	0.039	0.031	0.03	0.171	0.001	0	0.197	0	0	0	0.050	0.141	1.022
TCGA-OL-A66O-01	0	0	0.328	0.057	0	0.109	0	0.022	0.073	0	0	0.093	0.018	0.077	0.006	0.08	0	0	0.135	0	0	0.002	0.740	-0.004	1.069
TCGA-OL-A66P-01	0.018	0	0.156	0.201	0	0.094	0	0.03	0.055	0	0.012	0.052	0.026	0.092	0.057	0.156	0	0	0.051	0	0	0	0.010	0.418	0.908
TCGA-OL-A6VO-01	0.058	0	0	0.001	0	0.131	0	0.042	0.058	0	0.031	0	0	0.521	0.056	0.096	0	0	0	0	0.005	0	0.040	0.180	1.072
TCGA-OL-A6VQ-01	0.155	0	0.034	0.005	0	0.365	0.003	0	0	0	0.012	0.007	0.024	0	0.064	0.101	0.05	0.017	0.162	0	0	0	0.410	0.034	1.066
TCGA-OL-A6VR-01	0.156	0	0.178	0	0.014	0.104	0	0.052	0.007	0	0.015	0.009	0.007	0.238	0.016	0.154	0	0	0.051	0	0	0	0.040	0.159	1.018
TCGA-OL-A97C-01	0.024	0	0	0.151	0	0.117	0	0.043	0	0	0	0	0	0.391	0.088	0.176	0	0	0.012	0	0	0	0.010	0.282	0.998
TCGA-PE-A5DC-01	0.111	0	0.023	0.066	0	0.237	0	0	0.047	0	0.03	0.001	0	0.2	0.09	0.125	0	0	0.071	0	0	0	0.280	0.061	1.066
TCGA-PE-A5DD-01	0.116	0	0.064	0.141	0	0.328	0	0.011	0.041	0	0.032	0	0.026	0.038	0.052	0.068	0	0	0.082	0	0	0	0.220	0.069	1.066
TCGA-PE-A5DE-01	0.097	0	0.02	0.137	0	0.351	0.002	0.009	0.023	0	0.043	0.001	0.039	0.056	0.124	0.058	0.005	0	0.034	0	0	0	0.010	0.366	0.937
TCGA-PL-A8LV-01	0.112	0	0.11	0.091	0	0.157	0	0	0.042	0	0	0.04	0.049	0.155	0.147	0.049	0.008	0	0.033	0	0.006	0	0.020	0.232	0.993
TCGA-PL-A8LX-01	0.068	0	0.136	0	0	0.188	0	0	0.036	0	0	0.05	0.017	0	0.007	0.454	0	0	0.045	0	0	0	0.650	0.009	1.083
TCGA-PL-A8LY-01	0.09	0	0.084	0.013	0	0.248	0	0.056	0.037	0	0.024	0.031	0.03	0	0.012	0.328	0	0	0.046	0	0	0	0.320	0.048	1.060
TCGA-PL-A8LZ-01	0.035	0	0	0	0	0.308	0	0.106	0.031	0	0.009	0.022	0.014	0.06	0.2	0.105	0	0.061	0.048	0	0	0	0.010	0.373	0.939
TCGA-S3-A6ZF-01	0.155	0	0.044	0.049	0	0.269	0	0	0	0	0.021	0.002	0.013	0.057	0.057	0.244	0	0.023	0.066	0	0	0	0.190	0.082	1.047
TCGA-S3-A6ZG-01	0.143	0	0.111	0.024	0	0.178	0	0.012	0.01	0	0.001	0.024	0.008	0.09	0.027	0.244	0.01	0	0.118	0	0	0	0.420	0.031	1.059
TCGA-S3-A6ZH-01	0.077	0	0.106	0.083	0	0.133	0	0.027	0.047	0	0	0.028	0.038	0	0.045	0.176	0.014	0.105	0.12	0	0	0	0.350	0.043	1.052
TCGA-S3-AA0Z-01	0.094	0	0.07	0.067	0	0.147	0.003	0.104	0.018	0	0.053	0	0.012	0.173	0.081	0.176	0	0	0.002	0	0	0	0.050	0.142	1.029
TCGA-S3-AA10-01	0.029	0	0.049	0.025	0	0.24	0	0.149	0.006	0	0.027	0.047	0.031	0.147	0.212	0.037	0	0.002	0	0	0	0	0.000	0.487	0.874
TCGA-S3-AA11-01	0	0.002	0.018	0	0	0.373	0	0.048	0.097	0	0.07	0	0.004	0.307	0	0	0	0	0.082	0	0	0	0.370	0.040	1.099
TCGA-S3-AA12-01	0.095	0	0.15	0.052	0	0.171	0	0.009	0.047	0	0.039	0	0.029	0.038	0.031	0.269	0.005	0	0.063	0	0	0.001	0.910	-0.024	1.074
TCGA-S3-AA14-01	0.125	0	0.09	0.041	0	0.292	0	0.013	0	0	0.03	0	0.014	0	0.028	0.265	0	0.033	0.068	0	0	0	0.660	0.008	1.070
TCGA-S3-AA15-01	0.107	0.046	0.249	0.056	0	0.148	0.032	0	0	0	0.049	0	0.022	0.074	0.047	0.13	0	0	0.039	0	0	0	0.010	0.417	0.911
TCGA-S3-AA17-01	0.083	0	0.074	0.011	0	0.308	0.008	0	0.012	0	0.092	0	0.046	0.145	0.089	0.105	0	0.006	0.022	0	0	0	0.010	0.395	0.918
TCGA-UL-AAZ6-01	0.067	0	0.003	0	0	0.168	0	0	0.047	0	0	0.038	0	0.317	0.047	0.274	0	0	0.039	0	0	0	0.110	0.098	1.081
TCGA-UU-A93S-01	0.063	0	0.059	0	0	0.103	0	0.1	0.017	0	0.01	0.05	0	0.303	0.039	0.168	0	0.043	0.044	0	0	0.001	0.800	-0.012	1.109
TCGA-V7-A7HQ-01	0	0	0.205	0	0	0.21	0	0.005	0.078	0	0.018	0	0.026	0.15	0	0.164	0	0.028	0.116	0	0	0	0.070	0.116	1.026
TCGA-W8-A86G-01	0	0	0.05	0	0	0.297	0	0.02	0.083	0	0.067	0	0.047	0.127	0.044	0.01	0.017	0	0.238	0	0	0	0.410	0.034	1.074
TCGA-WT-AB41-01	0.074	0	0.044	0.161	0	0.17	0	0.05	0.092	0	0.001	0.002	0.01	0.069	0.034	0.224	0	0.045	0.024	0	0	0	0.010	0.426	0.904
TCGA-WT-AB44-01	0.038	0	0.099	0.029	0	0.181	0	0.058	0	0	0.012	0.017	0.006	0.112	0.018	0.348	0	0.041	0.039	0	0	0	0.370	0.041	1.070
TCGA-XX-A899-01	0.115	0.007	0	0.066	0	0.332	0.02	0	0	0	0.037	0	0.034	0.071	0.046	0.162	0	0.009	0.101	0	0	0	0.010	0.276	0.971
TCGA-XX-A89A-01	0.057	0	0.032	0.039	0	0.394	0	0	0	0	0.034	0	0.024	0.044	0.051	0.297	0.001	0.017	0.012	0	0	0	0.040	0.178	1.019
TCGA-Z7-A8R5-01	0.062	0	0	0.1	0	0.176	0	0.019	0	0	0	0	0.062	0.161	0.022	0.308	0	0	0.09	0	0	0	0.030	0.217	1.004

TCGA-Z7-A8R6-01	0.045	0	0.022	0.042	0	0.193	0	0.039	0.077	0	0.013	0.014	0.002	0.198	0.066	0.221	0.005	0	0.063	0	0	0	0.130	0.094	1.054
