# Supplementary material for: Analysis of Stemness and Prognosis of Subtypes in Breast Cancer Using the Transcriptome Sequencing Data
Source: J Oncol. 2022 Mar 9;2022:5694033. doi: 10.1155/2022/5694033 (PMC8926471; doi:10.1155/2022/5694033)
Supplement: Supplementary Materials — Figure legend S1. The relative abundance of immune cells in each sample based on the expression profile data of the sample was calculated by CIBERSORT. Table S1. Correlation analysis with mRNAsi and miRNAs. Table S2. Correlation analysis with mRNAsi and lncRNAs. Table S3. Correlation analysis with mRNAsi and mRNAs. [file 5694033.f1.zip › 5694033.f1/Table S1.pdf]

Table S1. Correlation analysis with mRNAsi and miRNAs

Features	Univ_beta	Univ_HR	Univ_95%CI_for_HR	Correlation	*P_value*	*P*_adj
hsa-mir-143	0.0000	1.0000	1.0000-1.0000	-0.3277	0.0000	0.0004
hsa-mir-221	0.0005	1.0005	1.0002-1.0007	-0.2829	0.0001	0.0034
