# Supplementary material for: Analysis of Stemness and Prognosis of Subtypes in Breast Cancer Using the Transcriptome Sequencing Data
Source: J Oncol. 2022 Mar 9;2022:5694033. doi: 10.1155/2022/5694033 (PMC8926471; doi:10.1155/2022/5694033)
Supplement: Supplementary Materials — Figure legend S1. The relative abundance of immune cells in each sample based on the expression profile data of the sample was calculated by CIBERSORT. Table S1. Correlation analysis with mRNAsi and miRNAs. Table S2. Correlation analysis with mRNAsi and lncRNAs. Table S3. Correlation analysis with mRNAsi and mRNAs. [file 5694033.f1.zip › 5694033.f1/Table S2.pdf]

Table S2. Correlation analysis with mRNAsi and lncRNAs

Features	Univ_beta	Univ_HR	Univ_95%CI_for_HR	Correlation	*P*_value	*P*_adj
AC006329.1	0.00130194	1.0013	1.00003-1.00257	0.5472092	1.10E-15	7.62E-13
LINC01638	0.0194901	1.01968	1.00241-1.03725	-0.483524699	4.11E-12	1.42E-09
AP001189.1	0.024824	1.02513	1.00235-1.04844	-0.463552974	3.89E-11	8.97E-09
AC087521.1	0.0632645	1.06531	1.02606-1.10606	-0.447119151	2.22E-10	3.84E-08
HOXD-AS2	0.0184783	1.01865	1.00529-1.03219	-0.430724274	1.16E-09	1.38E-07
AC017048.3	0.0508547	1.05217	1.02074-1.08457	-0.430397895	1.19E-09	1.38E-07
AC010457.1	0.0718071	1.07445	1.02137-1.13028	-0.426348312	1.77E-09	1.75E-07
AC126177.8	0.0829835	1.08652	1.01846-1.15914	-0.423436769	2.34E-09	2.02E-07
C17orf82	0.0130536	1.01314	1.00176-1.02465	-0.416953309	4.32E-09	3.32E-07
AC090337.1	0.0276567	1.02804	1.00733-1.04918	-0.411190273	7.37E-09	5.10E-07
TBX2-AS1	0.00441292	1.00442	1.0012-1.00765	-0.409985987	8.23E-09	5.18E-07
AC008742.1	0.0963217	1.10111	1.00735-1.2036	-0.404963849	1.30E-08	7.49E-07
LINC02202	0.0151223	1.01524	1.00463-1.02596	-0.400301139	1.97E-08	1.05E-06
LINC01711	0.0125428	1.01262	1.0049-1.0204	-0.397207313	2.59E-08	1.28E-06
AP007216.2	0.224837	1.25212	1.10821-1.41471	-0.395985261	2.88E-08	1.33E-06
AL031283.3	0.0616077	1.06355	1.02229-1.10647	-0.391338848	4.31E-08	1.86E-06
AC007998.3	0.0324562	1.03299	1.0116-1.05483	-0.387560857	5.95E-08	2.39E-06
AP000695.2	0.0156833	1.01581	1.00033-1.03152	-0.38705667	6.21E-08	2.39E-06
AC096708.3	0.0436756	1.04464	1.00837-1.08222	-0.376949118	1.44E-07	5.26E-06
MIR503HG	0.00246302	1.00247	1.00094-1.00399	-0.367792916	3.02E-07	1.05E-05
USP2-AS1	0.00385977	1.00387	1.00083-1.00691	0.358824831	6.10E-07	1.94E-05
AC027682.6	0.0160397	1.01617	1.00635-1.02609	-0.358683476	6.17E-07	1.94E-05
AL135960.1	0.0572516	1.05892	1.02207-1.0971	-0.357368019	6.83E-07	2.05E-05
AP001107.3	0.0721368	1.0748	1.01775-1.13506	-0.356593317	7.24E-07	2.09E-05
LINC02201	0.0247859	1.0251	1.00887-1.04158	-0.34895405	1.29E-06	3.57E-05
LINC01116	0.00122933	1.00123	1.00024-1.00222	-0.346786151	1.52E-06	4.03E-05
LINC00315	0.0430737	1.04401	1.0054-1.08412	-0.342626666	2.06E-06	5.27E-05
AC026904.3	0.124287	1.13234	1.02403-1.2521	-0.339240818	2.63E-06	6.50E-05
AC112715.1	0.0315765	1.03208	1.01123-1.05336	-0.334188558	3.77E-06	9.00E-05
LINC02599	0.0156409	1.01576	1.00612-1.0255	-0.331825098	4.46E-06	0.000102021
AC006441.4	0.0823852	1.08587	1.03616-1.13797	-0.331472258	4.57E-06	0.000102021
AC007389.5	0.0488871	1.0501	1.00195-1.10057	-0.330685059	4.83E-06	0.000104438
SNAI3-AS1	0.00611766	1.00614	1.0008-1.0115	-0.32456152	7.38E-06	0.000154737
SNHG7	0.000167436	1.00017	1.00001-1.00032	0.321552208	9.06E-06	0.000184354
AC091138.1	0.132683	1.14189	1.01167-1.28887	-0.318888308	1.08E-05	0.000214326
AC131956.2	0.170501	1.1859	1.01507-1.38548	-0.3172568	1.21E-05	0.00023241
BX322562.1	0.00763639	1.00767	1.00333-1.01202	-0.31652707	1.27E-05	0.000237396

AC084781.1	0.160947	1.17462	1.00052-1.37903	-0.315170622	1.39E-05	0.00025293
LINC01117	0.0223846	1.02264	1.00457-1.04103	-0.313668302	1.53E-05	0.000272152
AC008543.1	0.0116888	1.01176	1.00093-1.0227	0.308836685	2.10E-05	0.000363777
FAM225B	0.00498238	1.00499	1.00078-1.00922	-0.304211159	2.83E-05	0.000477595
HID1-AS1	0.0583442	1.06008	1.00235-1.12114	-0.293996821	5.36E-05	0.000882548
AP000812.1	0.059111	1.06089	1.02304-1.10014	-0.291660218	6.18E-05	0.000994178
LINC01788	0.0320386	1.03256	1.00398-1.06195	-0.291051044	6.41E-05	0.001008188
AC104260.1	0.234017	1.26367	1.11349-1.43409	-0.28890453	7.30E-05	0.001122243
AC126177.2	0.21315	1.23757	1.0128-1.51222	-0.28726398	8.05E-05	0.001211356
EPB41L4A-DT	0.0158611	1.01599	1.00561-1.02647	-0.283817846	9.88E-05	0.001454921
AC116563.1	0.185972	1.20439	1.07825-1.34529	-0.278906772	0.00013169	0.001862449
LINC00924	0.0127979	1.01288	1.00197-1.02391	-0.278605705	0.000134006	0.001862449
AC126177.4	0.121164	1.12881	1.0369-1.22887	-0.27849875	0.000134838	0.001862449
SMAD5-AS1	0.0405714	1.04141	1.01268-1.07095	-0.278190604	0.000137261	0.001862449
PICSAR	0.00110282	1.0011	1.00027-1.00194	-0.27529894	0.000162072	0.002156807
LINC02126	0.0224213	1.02267	1.00232-1.04344	-0.274511338	0.000169522	0.002213377
AL136298.1	0.0465619	1.04766	1.01836-1.07781	-0.268331283	0.000240067	0.003076414
LINC01423	0.0150389	1.01515	1.00171-1.02877	-0.267287354	0.000254392	0.003200713
EPB41L4A-AS1	0.000439844	1.00044	1-1.00088	0.264268259	0.000300418	0.003663038
AC021016.3	0.140374	1.1507	1.02577-1.29085	-0.26418904	0.000301724	0.003663038
AC022915.2	0.0496311	1.05088	1.0073-1.09636	-0.262283217	0.000334774	0.003986004
AC007546.1	0.0372991	1.038	1.00332-1.07388	-0.262006259	0.000339847	0.003986004
DNM3OS	0.000288821	1.00029	1.00002-1.00056	-0.259460713	0.000389932	0.004451906
MIR497HG	0.00592796	1.00595	1.00099-1.01093	-0.259341522	0.000392437	0.004451906
AP003351.1	0.00750816	1.00754	1.00288-1.01222	-0.258999699	0.000399703	0.004461207
AC106897.1	0.0202851	1.02049	1.00408-1.03717	-0.25704906	0.000443612	0.004872695
AC090371.1	0.208735	1.23212	1.07132-1.41705	-0.255481258	0.000482095	0.005188227
AC105094.2	0.0892996	1.09341	1.03549-1.15457	-0.255276879	0.000487333	0.005188227
AC022915.1	0.0513228	1.05266	1.01986-1.08652	-0.251878976	0.000582502	0.006107441
AC138207.5	0.00612919	1.00615	1.00002-1.01232	-0.250497971	0.00062587	0.006464208
AC005837.1	-0.043841	0.957106	0.917253-0.99869	0.249484723	0.000659563	0.006712024
AC010185.1	0.0297551	1.0302	1.00889-1.05196	-0.248739959	0.000685386	0.00687373
LINC00839	0.000602517	1.0006	1.00013-1.00107	0.246266899	0.000777969	0.007690776
LINC01013	0.0158974	1.01602	1.00057-1.03172	-0.244380673	0.000856175	0.008344695
AC009088.2	-0.593017	0.552657	0.306786-0.995581	0.241816048	0.000974127	0.009362444
AC096631.1	0.121973	1.12972	1.05875-1.20546	-0.241300744	0.000999557	0.009475253
AC147067.2	0.0655831	1.06778	1.01427-1.12412	-0.239707185	0.001082104	0.010119136
AC009063.3	0.0264402	1.02679	1.00973-1.04415	-0.239296356	0.001104376	0.010189709
LINC02511	0.0117717	1.01184	1.00384-1.01991	-0.237358744	0.001215194	0.011064659

AC091493.1	0.0377952	1.03852	1.01306-1.06462	-0.236958442	0.001239321	0.011137795
AC231981.1	-0.0527797	0.948589	0.901479-0.998161	0.235825187	0.001310024	0.011622266
AC069360.1	0.0189557	1.01914	1.00725-1.03117	-0.235035427	0.001361459	0.011925691
LYPLAL1-AS1	0.00279233	1.0028	1.00111-1.00448	-0.231304635	0.001630328	0.014102335
LINC01023	0.0101856	1.01024	1.00495-1.01555	-0.22919289	0.001803184	0.01540498
AL157402.2	0.00737866	1.00741	1.00118-1.01367	-0.227965619	0.001911159	0.016128315
AC091939.1	0.097489	1.1024	1.00367-1.21084	-0.22620431	0.002076423	0.017311862
AP001363.2	0.0790036	1.08221	1.0286-1.13861	-0.222179105	0.00250396	0.020497535
SLC12A5-AS1	0.0013385	1.00134	1.0005-1.00218	-0.222059889	0.002517761	0.020497535
AC114760.2	0.00230893	1.00231	1.00005-1.00457	-0.221407785	0.002594476	0.020600354
AP000904.1	0.0219972	1.02224	1.00914-1.03551	-0.221289775	0.002608584	0.020600354
AL358334.2	0.00503608	1.00505	1.00062-1.0095	-0.221197208	0.002619698	0.020600354
AL139246.1	0.0870608	1.09096	1.00129-1.18866	-0.220710555	0.00267884	0.020828732
DKFZp779M0652	0.0145749	1.01468	1.00077-1.02878	-0.219798187	0.002792988	0.021474975
AC078850.1	0.0148801	1.01499	1.00411-1.02599	-0.218906738	0.002908756	0.021981037
AL390208.1	0.0138114	1.01391	1.00195-1.02601	-0.218804294	0.002922334	0.021981037
AC015971.1	0.00838643	1.00842	1.00084-1.01607	-0.216440527	0.003252005	0.024197718
CACNA1G-AS1	0.0257168	1.02605	1.01021-1.04214	-0.21120557	0.004104638	0.030217124
LINC01150	0.0416632	1.04254	1.00251-1.08418	-0.210826653	0.004173542	0.030400958
AC005019.2	0.261132	1.2984	1.08251-1.55734	-0.209924242	0.004341848	0.030832666
AL049840.4	0.00368225	1.00369	1.00112-1.00627	-0.209809242	0.00436373	0.030832666
AC078955.1	0.0314962	1.032	1.00614-1.05852	-0.209794845	0.004366476	0.030832666
AP001993.1	0.0221451	1.02239	1.00778-1.03722	0.209392476	0.004443861	0.031062137
LINC01504	0.0198756	1.02007	1.00535-1.03502	-0.207412084	0.004842989	0.033513483
AC025040.1	0.0427094	1.04363	1.01401-1.07413	-0.206508636	0.005035528	0.034500845
AC007448.3	0.0144062	1.01451	1.00591-1.02318	-0.205510483	0.005256206	0.035553333
AL139420.2	0.217836	1.24338	1.04343-1.48166	-0.205352584	0.005291898	0.035553333
AC009054.2	0.0228954	1.02316	1.00455-1.04211	-0.204859401	0.005404781	0.03596258
C8orf87	0.0934899	1.098	1.01615-1.18644	-0.203137552	0.005815984	0.037660785
AC023886.1	0.00746747	1.0075	1.00208-1.01294	0.203067237	0.005833356	0.037660785
AL591767.1	0.0779658	1.08109	1.009-1.15832	-0.202944996	0.005863666	0.037660785
AL121899.1	0.0158837	1.01601	1.00597-1.02615	0.202711963	0.005921838	0.037660785
TEX41	0.00271067	1.00271	1.0008-1.00464	-0.202670995	0.005932118	0.037660785
AL365440.2	0.0359965	1.03665	1.0122-1.0617	-0.201227147	0.006304758	0.039662657
AP003973.2	0.113976	1.12073	1.00851-1.24542	-0.20035361	0.006540222	0.040773275
