# Supplementary material for: Analysis of Stemness and Prognosis of Subtypes in Breast Cancer Using the Transcriptome Sequencing Data
Source: J Oncol. 2022 Mar 9;2022:5694033. doi: 10.1155/2022/5694033 (PMC8926471; doi:10.1155/2022/5694033)
Supplement: Supplementary Materials — Figure legend S1. The relative abundance of immune cells in each sample based on the expression profile data of the sample was calculated by CIBERSORT. Table S1. Correlation analysis with mRNAsi and miRNAs. Table S2. Correlation analysis with mRNAsi and lncRNAs. Table S3. Correlation analysis with mRNAsi and mRNAs. [file 5694033.f1.zip › 5694033.f1/Table S3.pdf]

Table S3. Correlation analysis with mRNAsi and mRNAs

Features	Univ_beta	Univ_HR	Univ_95%CI_for_HR	Correlation	*P*_value	*P*_adj
COL1A1	9.85E-07	1	1-1	-0.658753139	3.83E-24	4.90E-21
SRPX2	0.000498931	1.0005	1.00006-1.00094	-0.6444283	7.38E-23	4.72E-20
PDGFRB	5.37E-05	1.00005	1.00001-1.00009	-0.624615303	3.44E-21	1.46E-18
TMEM204	0.000576913	1.00058	1.00006-1.0011	-0.59184573	1.12E-18	3.57E-16
HTRA1	3.73E-05	1.00004	1-1.00007	-0.586040357	2.91E-18	7.43E-16
P4HA3	0.00084545	1.00085	1.00035-1.00134	-0.579221925	8.73E-18	1.86E-15
CNRIP1	0.00153732	1.00154	1.00026-1.00282	-0.575611053	1.55E-17	2.82E-15
PODN	0.000296973	1.0003	1.00008-1.00051	-0.565788744	7.08E-17	1.13E-14
CERCAM	0.000139653	1.00014	1.00005-1.00022	-0.563859348	9.49E-17	1.35E-14
EXO1	-0.000363573	0.999636	0.999278-0.999995	0.557311538	2.53E-16	3.23E-14
PLXDC1	0.000277084	1.00028	1.00011-1.00045	-0.544021628	1.73E-15	2.01E-13
COPZ2	0.000396861	1.0004	1.00016-1.00063	-0.536041523	5.29E-15	5.63E-13
SSC5D	0.00032133	1.00032	1.00008-1.00056	-0.529218473	1.34E-14	1.32E-12
SCARF2	0.000499115	1.0005	1.00011-1.00089	-0.52539033	2.24E-14	2.05E-12
LHFPL6	0.000348409	1.00035	1.00002-1.00067	-0.523406646	2.92E-14	2.48E-12
CTSK	3.73E-05	1.00004	1.00001-1.00006	-0.520993767	4.01E-14	3.20E-12
TMEM119	0.000136831	1.00014	1.00001-1.00027	-0.518178824	5.79E-14	4.35E-12
RFX8	0.00593656	1.00595	1.0014-1.01053	-0.515902116	7.78E-14	5.52E-12
TGFB1	0.000144342	1.00014	1.00001-1.00028	-0.513277748	1.09E-13	7.33E-12
ADAM33	0.00181848	1.00182	1.0009-1.00274	-0.5128086	1.16E-13	7.39E-12
HSPA12B	0.00143895	1.00144	1.0006-1.00228	-0.50885016	1.91E-13	1.16E-11
MYL3	0.0076102	1.00764	1.00027-1.01506	-0.506949487	2.43E-13	1.41E-11
HSPB7	0.00137834	1.00138	1.00014-1.00262	-0.501987883	4.50E-13	2.50E-11
LOXL1	0.000214644	1.00021	1.00008-1.00034	-0.499798313	5.89E-13	3.14E-11
BMP1	0.000121816	1.00012	1.00004-1.00021	-0.499018408	6.48E-13	3.32E-11
IGFBP4	2.46E-05	1.00002	1-1.00005	-0.497543702	7.76E-13	3.82E-11
C1QTNF6	0.000173282	1.00017	1.00007-1.00027	-0.495352426	1.01E-12	4.79E-11
ADAMTS14	0.000952739	1.00095	1.00012-1.00179	-0.493222842	1.31E-12	5.97E-11
CORO2B	0.00258251	1.00259	1.00042-1.00475	-0.490881817	1.73E-12	7.62E-11
THY1	4.18E-05	1.00004	1.00002-1.00006	-0.490221587	1.87E-12	7.97E-11
PCDH12	0.000685705	1.00069	1.00011-1.00127	-0.489871554	1.95E-12	8.04E-11
USHBP1	0.00750236	1.00753	1.00281-1.01228	-0.485970812	3.09E-12	1.23E-10
MYC	2.80E-05	1.00003	1-1.00005	0.485525441	3.25E-12	1.26E-10
CLEC11A	0.000258395	1.00026	1.00006-1.00046	-0.483896302	3.94E-12	1.45E-10
EFEMP2	0.000147039	1.00015	1.00005-1.00024	-0.48382309	3.97E-12	1.45E-10
PTGIR	0.00248575	1.00249	1.00029-1.00469	-0.475494071	1.03E-11	3.66E-10
OLFML2B	5.97E-05	1.00006	1.00002-1.0001	-0.474116585	1.21E-11	4.17E-10

TMEM255B	0.00158773	1.00159	1.00026-1.00292	-0.470816105	1.75E-11	5.79E-10
EMILIN1	3.60E-05	1.00004	1.00001-1.00006	-0.470705708	1.77E-11	5.79E-10
DLL4	0.00089616	1.0009	1.00001-1.00178	-0.459392872	6.10E-11	1.95E-09
THSD1	0.00268963	1.00269	1.00036-1.00503	-0.456147081	8.63E-11	2.69E-09
TBX2	0.000412148	1.00041	1.0001-1.00073	-0.452643506	1.25E-10	3.80E-09
TGFB3	0.000248228	1.00025	1.00004-1.00046	-0.452115724	1.32E-10	3.93E-09
SNAI2	0.000184966	1.00018	1.00001-1.00036	-0.445041619	2.75E-10	7.99E-09
TIE1	0.00044531	1.00045	1.00003-1.00086	-0.444166185	3.01E-10	8.55E-09
GRID1	0.00392939	1.00394	1.00119-1.00669	-0.443469651	3.23E-10	8.98E-09
PEAR1	0.00139497	1.0014	1.00009-1.0027	-0.442854614	3.44E-10	9.36E-09
NLE1	0.000598319	1.0006	1.00011-1.00109	0.441590882	3.91E-10	1.04E-08
ELFN1	0.00201737	1.00202	1.00082-1.00323	-0.441232842	4.06E-10	1.06E-08
OLFML3	8.29E-05	1.00008	1.00002-1.00015	-0.437964837	5.64E-10	1.44E-08
LDB2	0.000751833	1.00075	1.00015-1.00136	-0.43461189	7.88E-10	1.97E-08
COL6A1	6.31E-06	1.00001	1-1.00001	-0.433643738	8.67E-10	2.13E-08
HEYL	0.0004735	1.00047	1.00015-1.0008	-0.433360808	8.92E-10	2.15E-08
KANK3	0.00341494	1.00342	1.00122-1.00563	-0.431857827	1.03E-09	2.45E-08
COL18A1	5.39E-05	1.00005	1.00003-1.00008	-0.430652716	1.16E-09	2.70E-08
ADAMTSL2	0.000995107	1.001	1.00021-1.00178	-0.430345121	1.20E-09	2.74E-08
SCG2	0.00183625	1.00184	1.00018-1.0035	-0.428587548	1.42E-09	3.19E-08
ESAM	0.000521805	1.00052	1.0001-1.00094	-0.426878839	1.68E-09	3.70E-08
GPC1	3.94E-05	1.00004	1-1.00008	-0.425399296	1.94E-09	4.20E-08
HIC1	0.000879526	1.00088	1.00047-1.00129	-0.424949301	2.02E-09	4.31E-08
FHOD1	0.000432132	1.00043	1.00015-1.00071	-0.424418517	2.13E-09	4.46E-08
AHCTF1	-0.000175528	0.999824	0.999691-0.999958	0.42367193	2.29E-09	4.72E-08
POPDC2	0.00190098	1.0019	1.00052-1.00329	-0.423411465	2.35E-09	4.76E-08
GJA4	0.00109254	1.00109	1.00036-1.00182	-0.421166417	2.90E-09	5.80E-08
SLC38A11	0.0111152	1.01118	1.00281-1.01961	-0.420391794	3.13E-09	6.15E-08
BLMH	0.00018572	1.00019	1-1.00037	0.419412376	3.43E-09	6.64E-08
CHPF	6.34E-05	1.00006	1.00002-1.0001	-0.418537265	3.72E-09	7.10E-08
SAMD14	0.00513943	1.00515	1.00065-1.00968	-0.417416785	4.14E-09	7.73E-08
GNG11	0.000482791	1.00048	1.0001-1.00087	-0.417319567	4.18E-09	7.73E-08
FOXS1	0.00497651	1.00499	1.00175-1.00824	-0.416779712	4.39E-09	8.02E-08
DPT	0.000252082	1.00025	1.00008-1.00042	-0.416389435	4.56E-09	8.20E-08
MMP19	0.000729982	1.00073	1.00023-1.00123	-0.413598686	5.91E-09	1.05E-07
COL14A1	7.29E-05	1.00007	1.00001-1.00014	-0.410363128	7.96E-09	1.39E-07
ARL9	0.000852782	1.00085	1.00027-1.00144	0.410136144	8.12E-09	1.39E-07
ACVRL1	0.000569011	1.00057	1.00023-1.00091	-0.410128327	8.13E-09	1.39E-07
CD99	6.35E-05	1.00006	1.00003-1.0001	-0.408076842	9.80E-09	1.65E-07
TLL2	0.00739762	1.00743	1.00033-1.01457	-0.405622448	1.22E-08	2.03E-07

COL6A2	3.34E-06	1	1-1.00001	-0.404734423	1.33E-08	2.17E-07
DCTPP1	0.000236961	1.00024	1.00006-1.00042	0.403837404	1.44E-08	2.33E-07
LCN1	0.0605235	1.06239	1.02524-1.10089	-0.403493777	1.48E-08	2.37E-07
DUSP1	6.27E-05	1.00006	1.00002-1.0001	-0.398824481	2.24E-08	3.54E-07
SMIM3	0.00052369	1.00052	1.00015-1.00089	-0.398700936	2.27E-08	3.54E-07
MAGEL2	0.00577405	1.00579	1.0007-1.01091	-0.398527421	2.30E-08	3.55E-07
SFRP2	1.83E-05	1.00002	1-1.00003	-0.398345628	2.34E-08	3.56E-07
COL5A3	6.60E-05	1.00007	1.00003-1.0001	-0.397362653	2.55E-08	3.84E-07
IGFBP6	0.000920228	1.00092	1.00049-1.00135	-0.388235396	5.62E-08	8.35E-07
MFAP4	0.000150968	1.00015	1.00004-1.00026	-0.387887857	5.79E-08	8.44E-07
SLC39A13	0.000260908	1.00026	1.00007-1.00045	-0.387835308	5.81E-08	8.44E-07
SEMA6B	0.000800878	1.0008	1.00007-1.00154	-0.386552462	6.48E-08	9.31E-07
PLPP7	0.00285579	1.00286	1.00069-1.00504	-0.386095538	6.74E-08	9.57E-07
JUN	5.26E-05	1.00005	1.00001-1.00009	-0.381408112	9.99E-08	1.40E-06
TMEM14B	-0.00028452	0.999716	0.999446-0.999985	0.379960969	1.13E-07	1.56E-06
GSC	0.00656342	1.00659	1.00014-1.01308	-0.379182751	1.20E-07	1.65E-06
HRC	0.0175505	1.01771	1.00816-1.02735	-0.378511928	1.27E-07	1.73E-06
STAB1	8.00E-05	1.00008	1.00003-1.00013	-0.378027021	1.32E-07	1.78E-06
RARRES2	7.97E-05	1.00008	1.00003-1.00013	-0.376701193	1.47E-07	1.96E-06
CD248	7.16E-05	1.00007	1.00003-1.00011	-0.372144267	2.13E-07	2.81E-06
CLDN5	0.000530285	1.00053	1.00026-1.0008	-0.371961976	2.17E-07	2.82E-06
SFRP4	6.92E-05	1.00007	1-1.00013	-0.370190273	2.50E-07	3.22E-06
C11orf96	0.000451982	1.00045	1.0002-1.00071	-0.369943655	2.55E-07	3.25E-06
SLC19A1	0.000338051	1.00034	1.00013-1.00055	0.369815005	2.57E-07	3.26E-06
COX4I2	0.00515455	1.00517	1.00146-1.00889	-0.368702525	2.81E-07	3.52E-06
VDAC3	4.62E-05	1.00005	1.00001-1.00009	0.367001632	3.22E-07	3.99E-06
ECSCR	0.00393403	1.00394	1.00148-1.00641	-0.366156835	3.44E-07	4.23E-06
CDCA4	0.000252336	1.00025	1.00001-1.0005	0.364858892	3.81E-07	4.64E-06
MS4A4E	0.0216975	1.02193	1.00612-1.038	-0.362414112	4.62E-07	5.55E-06
DYDC2	0.00238588	1.00239	1.00035-1.00443	0.362346045	4.64E-07	5.55E-06
ACKR3	0.000467405	1.00047	1.0001-1.00084	-0.361175908	5.09E-07	6.02E-06
ADCY4	0.00104326	1.00104	1.00009-1.00199	-0.360821431	5.23E-07	6.13E-06
CCR10	0.00844629	1.00848	1.003-1.01399	-0.359008801	6.02E-07	6.99E-06
MRPS15	8.62E-05	1.00009	1.00001-1.00016	0.356130223	7.50E-07	8.64E-06
IMPDH2	6.82E-05	1.00007	1-1.00013	0.355976026	7.59E-07	8.67E-06
GOLGA7	8.24E-05	1.00008	1.00001-1.00016	0.353585799	9.11E-07	1.03E-05
GAS6	8.55E-05	1.00009	1-1.00017	-0.349876356	1.20E-06	1.35E-05
TBCA	0.000121706	1.00012	1-1.00024	0.348518588	1.33E-06	1.48E-05
JTB	6.10E-05	1.00006	1-1.00012	0.348378114	1.35E-06	1.48E-05
P3H1	7.66E-05	1.00008	1.00002-1.00013	-0.348204846	1.36E-06	1.49E-05

RRP1	0.000124482	1.00012	1.00002-1.00023	0.347836498	1.40E-06	1.52E-05
COL16A1	4.21E-05	1.00004	1.00001-1.00007	-0.34745742	1.44E-06	1.55E-05
TMEM114	0.136721	1.14651	1.00964-1.30194	-0.347099629	1.48E-06	1.58E-05
PGF	0.000468615	1.00047	1.00023-1.0007	-0.346165462	1.59E-06	1.68E-05
NBL1	0.000100521	1.0001	1.00004-1.00017	-0.343401658	1.94E-06	2.04E-05
CAVIN3	0.000236524	1.00024	1.00001-1.00046	-0.342294342	2.11E-06	2.19E-05
SPNS2	0.000285791	1.00029	1.00004-1.00053	-0.341999367	2.15E-06	2.22E-05
PSMB4	1.91E-05	1.00002	1-1.00003	0.34169863	2.20E-06	2.25E-05
C16orf86	0.00804143	1.00807	1.004-1.01216	-0.341620824	2.21E-06	2.25E-05
TBXA2R	0.00242784	1.00243	1.00107-1.00379	-0.340308489	2.43E-06	2.45E-05
PDE1B	0.00117058	1.00117	1.0003-1.00204	-0.339563091	2.57E-06	2.57E-05
MAB21L1	0.00711249	1.00714	1.00288-1.01141	-0.338788964	2.72E-06	2.69E-05
HOXD8	0.00212254	1.00212	1.00039-1.00387	-0.338222803	2.83E-06	2.78E-05
ZDHHC1	0.00158936	1.00159	1.00045-1.00273	-0.337227141	3.04E-06	2.97E-05
PLAC9	0.000875413	1.00088	1.00046-1.00129	-0.336621835	3.17E-06	3.07E-05
ENTR1	0.000217601	1.00022	1.00002-1.00042	0.335349986	3.48E-06	3.34E-05
ENG	9.26E-05	1.00009	1.00001-1.00017	-0.333498905	3.96E-06	3.78E-05
ABRACL	0.000101587	1.0001	1-1.0002	0.33161632	4.52E-06	4.28E-05
PCOLCE	1.30E-05	1.00001	1-1.00002	-0.331149233	4.68E-06	4.36E-05
RRP9	0.000321681	1.00032	1.00005-1.0006	0.331146773	4.68E-06	4.36E-05
MSX1	0.00109644	1.0011	1.00022-1.00198	-0.329712735	5.17E-06	4.79E-05
GPIHBP1	0.0032282	1.00323	1.0016-1.00487	-0.32887574	5.48E-06	5.04E-05
MMP17	0.00153057	1.00153	1.0001-1.00297	-0.328233595	5.73E-06	5.20E-05
CTSG	0.00525537	1.00527	1.00188-1.00867	-0.328170068	5.75E-06	5.20E-05
HOXD9	0.00202985	1.00203	1.00048-1.00359	-0.328123421	5.77E-06	5.20E-05
RAPGEF3	0.00136107	1.00136	1.00054-1.00218	-0.328008832	5.82E-06	5.20E-05
KIFC3	0.00025902	1.00026	1.00008-1.00044	-0.327887052	5.87E-06	5.21E-05
PLEC	1.86E-05	1.00002	1-1.00004	-0.325369045	6.98E-06	6.15E-05
MRPL17	0.0001818	1.00018	1.00001-1.00036	0.324890229	7.21E-06	6.32E-05
MTMR4	-0.000227557	0.999772	0.999562-0.999983	0.323700691	7.83E-06	6.80E-05
NDUFV3	0.000246718	1.00025	1.00002-1.00047	0.32316475	8.12E-06	7.01E-05
C20orf27	0.000191053	1.00019	1.00004-1.00035	0.322869394	8.28E-06	7.10E-05
SNAPC4	0.000368099	1.00037	1.00001-1.00073	0.320366809	9.81E-06	8.36E-05
HIGD1B	0.0050173	1.00503	1.00206-1.00801	-0.320246524	9.89E-06	8.37E-05
SSSCA1	0.000450805	1.00045	1.00005-1.00085	0.319968989	1.01E-05	8.48E-05
ADRA1D	0.0205158	1.02073	1.00898-1.03262	-0.31939375	1.05E-05	8.75E-05
ADCYAP1R1	0.00157357	1.00157	1.00082-1.00233	-0.315666482	1.34E-05	0.000111536
EMP3	0.000200988	1.0002	1.00004-1.00036	-0.315355616	1.37E-05	0.000113124
SPON2	4.88E-05	1.00005	1.00002-1.00008	-0.314680374	1.43E-05	0.000117067
TSC22D3	8.38E-05	1.00008	1-1.00017	-0.314644253	1.44E-05	0.000117067

ARHGAP6	0.00192746	1.00193	1.00041-1.00345	-0.313771336	1.52E-05	0.000123224
PPIC	0.000231358	1.00023	1.00004-1.00042	-0.313126353	1.59E-05	0.000127759
LGALS1	1.37E-05	1.00001	1-1.00002	-0.312882125	1.62E-05	0.000128541
RCN3	4.93E-05	1.00005	1.00002-1.00008	-0.312843303	1.62E-05	0.000128541
SRPX	0.000281911	1.00028	1.0001-1.00046	-0.312590482	1.65E-05	0.000129727
GLIS1	0.00346855	1.00347	1.00157-1.00538	-0.31245291	1.66E-05	0.000129727
COX7A1	0.00149209	1.00149	1.00073-1.00226	-0.312421968	1.66E-05	0.000129727
MSC	0.000296849	1.0003	1.00009-1.0005	-0.312324473	1.68E-05	0.000129768
PRND	0.0019414	1.00194	1.00026-1.00363	-0.312072135	1.70E-05	0.000130924
SAP130	-0.000403166	0.999597	0.999202-0.999992	0.312005155	1.71E-05	0.000130924
RABAC1	0.000127344	1.00013	1.00004-1.00021	-0.310798568	1.85E-05	0.000140819
LRRC18	0.0199627	1.02016	1.002-1.03865	-0.310557292	1.88E-05	0.000142204
PYGM	0.00840412	1.00844	1.00195-1.01497	-0.308090436	2.21E-05	0.00016589
LACTB2	0.000581428	1.00058	1.00012-1.00104	0.307642552	2.27E-05	0.000169754
FAM162B	0.0114156	1.01148	1.00065-1.02243	-0.307545537	2.29E-05	0.000169826
RPUSD1	0.000278802	1.00028	1.00001-1.00055	0.307367914	2.31E-05	0.000170787
ACSM5	0.00354388	1.00355	1.00018-1.00693	-0.306759893	2.40E-05	0.000176577
ELOC	0.000211149	1.00021	1.00006-1.00036	0.304658246	2.75E-05	0.000200845
SDHAF4	0.00114961	1.00115	1.00029-1.00201	0.304118397	2.85E-05	0.00020669
TONSL	0.000183336	1.00018	1.00002-1.00035	0.303672309	2.93E-05	0.000211435
NTMT1	0.000560929	1.00056	1.00007-1.00105	0.302883968	3.08E-05	0.000221029
CHST13	0.00935324	1.0094	1.00376-1.01506	-0.302605922	3.13E-05	0.000223697
RAB11FIP4	0.000141164	1.00014	1-1.00028	0.300963633	3.48E-05	0.00024674
MRPL15	8.50E-05	1.00008	1-1.00017	0.300418948	3.60E-05	0.000253921
SGPP2	-0.0006712	0.999329	0.998686-0.999972	0.299911514	3.71E-05	0.000260691
RASGRP4	0.00443984	1.00445	1.00057-1.00834	-0.299785571	3.74E-05	0.000261321
FAM72C	0.00451573	1.00453	1.00126-1.0078	0.299476302	3.82E-05	0.000264981
SMIM19	0.000218517	1.00022	1.00002-1.00042	0.299098347	3.91E-05	0.00026985
CHST7	0.00519551	1.00521	1.00204-1.00839	-0.298275674	4.11E-05	0.000282532
RBMXL1	-0.000622276	0.999378	0.998819-0.999937	0.297303052	4.37E-05	0.000298538
TKT	2.28E-05	1.00002	1-1.00004	0.296088259	4.71E-05	0.000318638
CACNA1G	0.00138361	1.00138	1.00044-1.00233	-0.296041244	4.72E-05	0.000318638
LAMA2	0.000327774	1.00033	1.00006-1.00059	-0.295993574	4.74E-05	0.000318638
MRPS26	0.00019346	1.00019	1.00001-1.00038	0.295844583	4.78E-05	0.000319899
CPXM1	5.87E-05	1.00006	1.00003-1.00009	-0.29531647	4.94E-05	0.000328768
ATP6V0D2	0.00336351	1.00337	1.00176-1.00498	-0.294924452	5.06E-05	0.000335053
MMP28	0.000730176	1.00073	1.00033-1.00113	-0.294670247	5.14E-05	0.000338578
CRYBB1	0.014635	1.01474	1.00608-1.02348	-0.293955612	5.37E-05	0.000351945
RGS3	0.000253975	1.00025	1.00003-1.00047	-0.293614284	5.48E-05	0.000357548
NDUFAF2	0.000661947	1.00066	1.00008-1.00124	0.291934944	6.08E-05	0.000394127

NAGLU	0.000572247	1.00057	1.00021-1.00093	-0.291490203	6.24E-05	0.000402883
FOS	3.34E-05	1.00003	1.00001-1.00006	-0.29048131	6.64E-05	0.000426142
TMEM70	0.000410727	1.00041	1.00008-1.00074	0.289773928	6.93E-05	0.000442531
EIF4EBP1	5.94E-05	1.00006	1.00001-1.00011	0.288705615	7.39E-05	0.000469595
PLAU	4.48E-05	1.00004	1.00002-1.00007	-0.288389734	7.53E-05	0.000476222
SOD1	3.02E-05	1.00003	1-1.00006	0.287323331	8.02E-05	0.000505146
FOLR2	0.000181046	1.00018	1.00008-1.00029	-0.285808176	8.78E-05	0.000550203
ADRB3	0.0310242	1.03151	1.00722-1.05639	-0.285493412	8.95E-05	0.000557858
LYL1	0.00128606	1.00129	1.0002-1.00238	-0.285393647	9.00E-05	0.000558448
MAN1C1	0.000692546	1.00069	1.00011-1.00128	-0.285243595	9.08E-05	0.000560722
FST	0.000208614	1.00021	1.00003-1.00038	-0.283273907	0.000102037	0.000626941
LCN6	0.0621953	1.06417	1.01738-1.11311	-0.282313393	0.000107965	0.000660191
NTRK1	0.00746986	1.0075	1.00025-1.0148	-0.282230365	0.000108493	0.000660256
TALDO1	4.48E-05	1.00004	1.00001-1.00008	0.281986605	0.000110055	0.000664541
TSPAN4	0.000330477	1.00033	1.00012-1.00054	-0.2819584	0.000110237	0.000664541
CHML	-0.000193227	0.999807	0.999637-0.999977	0.281462428	0.000113485	0.000680911
PAMR1	0.000953032	1.00095	1.0002-1.00171	-0.281045683	0.000116284	0.000694441
RPL36A	0.120975	1.1286	1.03942-1.22543	0.280924239	0.000117111	0.00069613
CDC34	0.000212596	1.00021	1.00002-1.00041	0.276606687	0.000150374	0.000889713
B4GALT2	9.78E-05	1.0001	1.00001-1.00019	0.275957776	0.000156077	0.000919197
THBD	0.000243421	1.00024	1.00001-1.00048	-0.275766593	0.000157795	0.000925053
CFD	0.000289237	1.00029	1.00008-1.00049	-0.275618896	0.000159134	0.000928646
KLF16	0.000429417	1.00043	1.00007-1.00079	0.275373253	0.000161385	0.000937502
ASH2L	9.22E-05	1.00009	1.00001-1.00017	0.275250218	0.000162524	0.000939845
LTC4S	0.155163	1.16785	1.07808-1.26509	-0.274853451	0.000166247	0.000957046
TPMT	-0.000394888	0.999605	0.999217-0.999994	0.273245022	0.000182172	0.001044015
ZFP36	5.52E-05	1.00006	1.00003-1.00008	-0.272859744	0.000186192	0.00106229
LRRC70	0.0130606	1.01315	1.00312-1.02327	-0.272514746	0.000189862	0.001078413
CC2D2B	0.0288141	1.02923	1.00123-1.05802	-0.27239683	0.000191131	0.001080821
DCSTAMP	0.00306441	1.00307	1.00029-1.00585	-0.271927133	0.000196268	0.001104978
WISP2	7.85E-05	1.00008	1.00003-1.00012	-0.27162427	0.000199648	0.001119077
FAM92A	0.000336795	1.00034	1.00001-1.00067	0.270554491	0.000212025	0.001183266
IFI27L2	0.000508633	1.00051	1.0001-1.00092	-0.270150579	0.000216881	0.001205106
ZNF672	-0.000323314	0.999677	0.999396-0.999958	0.269780698	0.000221419	0.001224995
WRB	0.000282434	1.00028	1.00002-1.00055	0.269094209	0.000230077	0.001267405
RHOB	5.78E-05	1.00006	1-1.00011	-0.267228509	0.000255223	0.00139989
PBDC1	0.000375349	1.00038	1.00009-1.00066	0.265962625	0.000273715	0.001494904
SEMA7A	0.000535903	1.00054	1.00011-1.00096	-0.26578235	0.000276447	0.001503403
BRF2	0.000780007	1.00078	1.0001-1.00146	0.265341623	0.000283235	0.001533788
GLYCTK	0.00310965	1.00311	1.00058-1.00565	-0.264695325	0.000293469	0.001582502

CPA1	0.0311231	1.03161	1.00421-1.05977	-0.264524231	0.000296235	0.001590706
EGFLAM	0.000670079	1.00067	1.00004-1.0013	-0.264059714	0.000303867	0.001624864
CYC1	4.56E-05	1.00005	1.00001-1.00008	0.2636321	0.000311055	0.001656366
CRIP2	7.93E-05	1.00008	1.00001-1.00015	-0.263198941	0.000318496	0.001688953
ZSCAN5A	-0.00319234	0.996813	0.994029-0.999604	0.262688524	0.000327476	0.001727317
GATD1	0.000192888	1.00019	1.00005-1.00033	0.262634873	0.000328434	0.001727317
ZFPM1	0.00294183	1.00295	1.00124-1.00466	-0.262404514	0.000332574	0.001741926
PLPP2	9.52E-05	1.0001	1.00001-1.00018	0.261013258	0.000358629	0.001870726
CORO6	0.00352083	1.00353	1.00065-1.00641	-0.260610945	0.000366509	0.001904058
CMA1	0.0111105	1.01117	1.00269-1.01973	-0.259895324	0.000380923	0.00197093
TRMT61A	0.000339565	1.00034	1.00001-1.00067	0.258896493	0.000401922	0.002071194
ZNF706	0.000108874	1.00011	1-1.00021	0.257257708	0.000438711	0.002251698
INAFM1	0.000421001	1.00042	1.00018-1.00066	-0.256344797	0.000460533	0.002354245
CDC42EP5	0.000853319	1.00085	1.00035-1.00136	-0.256217084	0.000463665	0.002358707
AMIGO3	0.233593	1.26313	1.0729-1.48709	-0.256158938	0.000465097	0.002358707
GRASP	0.00134454	1.00135	1.00071-1.00199	-0.254214261	0.000515426	0.002603615
FOSB	9.69E-05	1.0001	1.00004-1.00016	-0.25412646	0.000517813	0.002605374
PHLDA3	0.000151681	1.00015	1.00004-1.00027	-0.253131225	0.000545593	0.002734381
OCSTAMP	0.0168393	1.01698	1.0069-1.02716	-0.252403353	0.000566774	0.002829442
EVA1B	0.000209609	1.00021	1.0001-1.00031	-0.251637301	0.000589885	0.002933357
MRPS21	4.92E-05	1.00005	1-1.0001	0.251168216	0.000604463	0.002994199
CRYGS	0.00286963	1.00287	1.00068-1.00507	-0.250019968	0.000641562	0.003165699
PLPBP	0.000157862	1.00016	1.00003-1.00028	0.249372834	0.000663384	0.00326079
POLR2E	0.000101195	1.0001	1.00001-1.00019	0.248341306	0.000699589	0.003425573
CAVIN2	0.000589069	1.00059	1.00006-1.00112	-0.248221725	0.000703902	0.003433535
ZNF688	0.00204379	1.00205	1.0007-1.00339	-0.248097383	0.000708412	0.003442398
SCFD2	0.000403719	1.0004	1.00001-1.0008	0.246556827	0.000766548	0.003710791
RPL8	2.81E-06	1	1-1.00001	0.246244649	0.000778852	0.003752054
SLC37A4	0.000414672	1.00041	1.00002-1.00081	0.246192011	0.000780944	0.003752054
RHOC	7.46E-05	1.00007	1.00002-1.00013	-0.245785185	0.000797291	0.003802389
SRRM3	0.00179244	1.00179	1.00089-1.00269	-0.245783197	0.000797371	0.003802389
ZNF891	0.000862488	1.00086	1.00001-1.00172	0.244997769	0.000829827	0.003942451
RRP8	0.000704631	1.0007	1.00005-1.00136	0.2446297	0.000845451	0.004001804
TGFBI	1.49E-05	1.00001	1.00001-1.00002	-0.24322735	0.000907491	0.004272058
GAP43	0.00385725	1.00386	1.0001-1.00765	-0.243189268	0.000909233	0.004272058
CD276	7.99E-05	1.00008	1.00001-1.00015	-0.243009629	0.00091749	0.004295064
HES6	0.000288062	1.00029	1.00001-1.00057	0.242096174	0.000960554	0.004480247
CYTL1	0.0024605	1.00246	1.00041-1.00452	-0.241659627	0.000981783	0.004562613
BOP1	6.69E-05	1.00007	1.00003-1.00011	0.241401146	0.000994555	0.004605222
CCL16	0.0619775	1.06394	1.02391-1.10553	-0.241298812	0.000999654	0.00461212

C8orf33	8.91E-05	1.00009	1-1.00017	0.241166229	0.001006295	0.00462606
GADD45B	0.000185298	1.00019	1.00006-1.00031	-0.240934294	0.00101801	0.004663144
LMF1	0.000791435	1.00079	1.00018-1.0014	-0.240470711	0.001041803	0.004755086
RAMP2	0.000537679	1.00054	1.00005-1.00103	-0.24021726	0.001055025	0.004798301
AGPAT4	0.000534602	1.00053	1.00018-1.00089	-0.240061124	0.001063248	0.004818548
CABP1	0.0175368	1.01769	1.00467-1.03088	-0.239231984	0.001107904	0.004986286
BAG4	0.00011371	1.00011	1.00001-1.00022	0.239229069	0.001108064	0.004986286
PDE2A	0.000724203	1.00072	1.00012-1.00133	-0.238628099	0.001141503	0.00511874
MACROD1	0.000379285	1.00038	1.00009-1.00067	0.238414395	0.001153615	0.005154966
FGF7	0.000139086	1.00014	1.00002-1.00026	-0.238329556	0.001158456	0.005158562
MEMO1	0.00120478	1.00121	1.00021-1.0022	0.238146859	0.001168945	0.005187192
LCNL1	0.00648902	1.00651	1.00128-1.01176	-0.236411253	0.001273012	0.005629443
PTGER1	0.0191861	1.01937	1.00805-1.03082	-0.234824005	0.001375538	0.006061855
NTHL1	0.000484359	1.00048	1.00009-1.00088	0.234720614	0.001382472	0.006071474
PXYLP1	-0.000769422	0.999231	0.998574-0.999888	0.234583823	0.001391694	0.006091045
CST3	1.41E-05	1.00001	1-1.00002	-0.234497728	0.001397528	0.006095701
IL1RL2	0.00137414	1.00138	1.00002-1.00274	0.234079686	0.001426171	0.006199479
RELCH	0.000239857	1.00024	1.00001-1.00047	0.233905961	0.001438232	0.006230712
PSMD4	2.94E-05	1.00003	1.00001-1.00005	0.233306856	0.001480542	0.006392338
PKP3	8.65E-05	1.00009	1-1.00017	0.232865193	0.001512458	0.006508151
OSBPL5	0.00040833	1.00041	1.00005-1.00077	-0.232523387	0.001537588	0.006594084
CACNA2D4	0.00172424	1.00173	1.00047-1.00298	-0.232317345	0.00155292	0.006637564
OR2L3	0.435593	1.54588	1.10954-2.15381	-0.231769197	0.001594391	0.006769048
OAF	0.000271805	1.00027	1.0001-1.00044	-0.231758331	0.001595224	0.006769048
HOMER1	-0.00112605	0.998875	0.997831-0.999919	0.231701644	0.001599572	0.006769048
NRP1	4.86E-05	1.00005	1.00001-1.00009	-0.230946778	0.001658512	0.006994449
TUBA1A	2.38E-05	1.00002	1.00001-1.00004	0.230880495	0.001663781	0.006994449
MRPL23	0.000276309	1.00028	1-1.00055	0.23054045	0.001691053	0.007085788
RASD1	0.000137578	1.00014	1.00002-1.00025	-0.230335083	0.00170772	0.007132243
RDH8	0.144724	1.15572	1.08337-1.2329	-0.229953716	0.001739069	0.007239514
TPPP3	0.000416573	1.00042	1.00004-1.0008	-0.229874627	0.001745636	0.007243256
DBX2	0.0239266	1.02422	1.00176-1.04717	-0.229801041	0.001751766	0.007245169
SSNA1	0.000153091	1.00015	1.00003-1.00028	0.229442837	0.001781887	0.007345974
3-Sep	-0.000204989	0.999795	0.999605-0.999985	0.228981461	0.00182138	0.00748464
ZDHHC15	0.00104476	1.00105	1.00033-1.00176	0.228897121	0.001828685	0.007490573
MMP9	2.18E-06	1	1月1日	-0.228715304	0.001844523	0.007514872
ZNF251	0.000291835	1.00029	1.00002-1.00057	0.228694115	0.001846377	0.007514872
ANKRD46	0.00040784	1.00041	1.00011-1.0007	0.228119969	0.001897263	0.007697466
ZNF16	0.000659662	1.00066	1.0001-1.00122	0.227872705	0.001919568	0.007763317
NCLN	0.000160082	1.00016	1.00003-1.00029	0.227753922	0.001930368	0.007769975

GRIA1	0.0249555	1.02527	1.01217-1.03854	-0.227720971	0.001933374	0.007769975
B4GALT7	0.000462564	1.00046	1.0002-1.00073	-0.227528306	0.001951034	0.007816369
LINGO3	0.00648921	1.00651	1.0011-1.01195	-0.22710144	0.001990686	0.007950304
FEZ1	0.000844278	1.00084	1.00034-1.00135	-0.226734841	0.002025324	0.008063441
TIMP1	1.02E-05	1.00001	1-1.00002	-0.226474155	0.002050288	0.008137477
INKA1	0.00159523	1.0016	1.00049-1.00271	-0.22612245	0.002084411	0.008247297
EFNB1	0.000201141	1.0002	1.00004-1.00036	-0.225475843	0.002148497	0.008461989
HGH1	0.000131344	1.00013	1.00005-1.00022	0.225385736	0.002157568	0.008461989
RGS10	0.00010047	1.0001	1-1.0002	0.225365593	0.002159601	0.008461989
TIMM13	0.000155576	1.00016	1.00004-1.00028	0.225310621	0.002165157	0.008461989
FKBP10	4.18E-05	1.00004	1.00001-1.00007	-0.224750729	0.00222249	0.008659581
THEM6	0.000143724	1.00014	1.00005-1.00023	0.224584404	0.002239786	0.008700446
CACNA1B	0.000395596	1.0004	1.00006-1.00074	0.224331468	0.002266323	0.008776851
MADCAM1	0.00716562	1.00719	1.00287-1.01153	-0.222927481	0.002418885	0.00933938
SERTM2	0.0307161	1.03119	1.00744-1.0555	-0.222458585	0.002471876	0.009515235
NFRKB	0.000362411	1.00036	1.00003-1.0007	0.222309332	0.002488964	0.009551403
SERPINE1	4.06E-05	1.00004	1.00002-1.00006	-0.222246235	0.00249622	0.009551403
SLC22A1	0.0103213	1.01037	1.00133-1.0195	-0.222031689	0.002521035	0.009617561
RNF126	0.000345298	1.00035	1.00005-1.00064	0.221772283	0.002551338	0.009704198
DAPK3	0.000267249	1.00027	1.00009-1.00044	-0.220871685	0.002659125	0.010078165
ADH1C	0.00311097	1.00312	1.00096-1.00528	-0.220820037	0.00266543	0.010078165
JOSD2	0.000607602	1.00061	1.00026-1.00096	-0.220007846	0.002766374	0.010428986
CSRNP1	0.000277681	1.00028	1.00001-1.00054	-0.219044034	0.002890648	0.010865434
PLXND1	8.61E-05	1.00009	1.00001-1.00016	-0.218963582	0.002901246	0.010873292
ZNF703	0.000332418	1.00033	1.00017-1.0005	-0.218092805	0.003018232	0.011278657
ACP5	1.74E-05	1.00002	1.00001-1.00003	-0.21783564	0.003053588	0.01137751
RGS4	0.000257515	1.00026	1.00002-1.0005	-0.21740873	0.00311311	0.011565565
VEGFD	0.00358228	1.00359	1.00092-1.00626	-0.215631525	0.00337233	0.012492283
ABCA3	0.000122536	1.00012	1-1.00024	0.215023152	0.003465447	0.012763254
OPN4	0.0322206	1.03275	1.00623-1.05996	-0.215023113	0.003465453	0.012763254
UBTD1	0.000877201	1.00088	1.00039-1.00136	-0.214198625	0.003595352	0.01320362
F3	0.000179102	1.00018	1.00002-1.00034	-0.214019028	0.003624223	0.013271511
HOXD1	0.018532	1.0187	1.00664-1.03092	-0.213859991	0.003649964	0.013292879
DUPD1	0.225486	1.25293	1.01826-1.54169	-0.213800198	0.003659685	0.013292879
FFAR3	0.0967684	1.10161	1.02509-1.18384	-0.213790505	0.003661263	0.013292879
VPS25	0.000241258	1.00024	1.00001-1.00048	0.213341412	0.003735048	0.013522355
PTGDS	3.17E-05	1.00003	1.00001-1.00005	-0.212548285	0.003868638	0.013956833
APBA2	0.000307618	1.00031	1.00003-1.00059	0.212500061	0.003876898	0.013956833
ATP6V0B	6.96E-05	1.00007	1.00002-1.00012	0.211876594	0.003985124	0.014233565
CCM2	0.000247312	1.00025	1.00006-1.00044	-0.211875663	0.003985288	0.014233565

PTDSS2	0.000352112	1.00035	1.00005-1.00066	0.211864897	0.00398718	0.014233565
KCNJ8	0.000709508	1.00071	1.00023-1.00119	-0.211749264	0.004007559	0.014266462
G0S2	0.000103551	1.0001	1-1.0002	-0.21050398	0.004233037	0.015021271
GPR34	0.000810873	1.00081	1.00016-1.00147	-0.210449817	0.004243098	0.015021271
MRPS28	0.000611602	1.00061	1.00003-1.00119	0.210019917	0.00432372	0.015264402
PFKL	8.71E-05	1.00009	1.00003-1.00014	0.209223258	0.004476772	0.015761196
GPAT4	6.71E-05	1.00007	1.00003-1.00011	0.208468363	0.004626274	0.016225601
LSM7	0.000217442	1.00022	1.00003-1.00041	0.20842959	0.004634072	0.016225601
TMEM178A	0.00633868	1.00636	1.00073-1.01202	-0.208355397	0.004649027	0.016233489
NUP50	-0.000222683	0.999777	0.999562-0.999992	0.208287211	0.00466281	0.016237252
DDHD2	8.43E-05	1.00008	1.00003-1.00014	0.208217621	0.004676914	0.016242111
FBXL6	0.000236639	1.00024	1.0001-1.00037	0.208121653	0.004696428	0.016265677
TSTD1	0.000195227	1.0002	1.00001-1.00038	0.207709957	0.004780971	0.016513732
NME1-NME2	0.00777781	1.00781	1.00297-1.01267	0.206296171	0.005081793	0.017505474
MYLK2	0.0151941	1.01531	1.00063-1.0302	0.2060124	0.005144179	0.017672743
LAMC3	0.000271481	1.00027	1.0001-1.00044	-0.20556045	0.005244956	0.017970654
EGLN1	-0.000218671	0.999781	0.999597-0.999966	0.205296491	0.00530463	0.018126515
ZBTB16	0.00284374	1.00285	1.00001-1.00569	-0.204909496	0.005393217	0.018380084
CRHBP	0.0131495	1.01324	1.00434-1.02221	-0.204721835	0.00543665	0.018432253
RECQL4	0.000164443	1.00016	1.00003-1.0003	0.204685561	0.005445081	0.018432253
GPX3	5.33E-05	1.00005	1-1.0001	-0.204656721	0.005451793	0.018432253
NPAS1	0.00486612	1.00488	1.00019-1.00958	-0.204373863	0.005518015	0.018606921
ENO3	0.00124365	1.00124	1.00027-1.00222	0.20338133	0.005756116	0.019358728
FBXL8	0.00271056	1.00271	1.00014-1.0053	-0.202886383	0.005878249	0.019717592
SLC37A2	0.00040042	1.0004	1.00016-1.00064	-0.202718073	0.005920306	0.019806679
FGF8	0.0125222	1.0126	1.0016-1.02373	0.202319569	0.006020957	0.020065991
PIGN	0.00024845	1.00025	1.00008-1.00042	0.202287127	0.006029218	0.020065991
TMEM165	0.000124901	1.00012	1.00003-1.00022	0.201906088	0.006127005	0.020321449
FSTL3	0.000467539	1.00047	1.00015-1.00078	-0.201864444	0.006137777	0.020321449
P2RX6	0.0080248	1.00806	1.00305-1.01309	-0.201731926	0.00617217	0.020382515
CASTOR1	0.0038917	1.0039	1.00014-1.00767	-0.200289326	0.006557856	0.021600359
EFHD1	3.50E-05	1.00003	1-1.00007	0.200124983	0.00660313	0.021693573
